# Arctic Soil C and N Cycling Are Linked With Microbial Adaptations During Drought

**DOI:** 10.1111/gcb.70502

**Published:** 2025-09-18

**Authors:** Theis Thomsen, Morten Dencker Schostag, Anders Priemé, Jonathan Donhauser

**Affiliations:** ^1^ Department of Biology University of Copenhagen Copenhagen Denmark; ^2^ Department of Biotechnology and Biomedicine Technical University of Denmark Lyngby Denmark; ^3^ Center for Volatile Interactions (VOLT) University of Copenhagen Copenhagen Denmark

**Keywords:** climate change, extracellular enzyme activities, greenhouse gas emissions, metatranscriptome, microbial C:N:P stoichiometry, microbial stress response, microcosm experiment

## Abstract

Climate change increases the frequency and intensity of drought events, yet the mechanisms of microbe‐mediated soil carbon (C) and nitrogen (N) cycling under drought are poorly understood. We conducted a microcosm experiment with a Greenlandic soil subjected to five levels of drought, reducing water content from 180% to 15% over the course of 3 weeks followed by rewetting, mimicking a natural drought event. We linked changes in microbial gene expression related to stress response as well as C and N cycling with greenhouse gas (GHG) emissions, extracellular enzyme activities, and soil C and N status. Maximum changes in gene expression occurred at intermediate levels of drought (80% water content), characterized by acclimation of microbial physiology to drought conditions, including production of osmolytes as well as cell wall and membrane modifications. This peak in gene expression changes marked a tipping point associated with a pronounced decline in microbial respiration as well as extracellular enzyme activities under more intense drought conditions. Interestingly, C‐cycling gene expression correlated with soil dissolved organic nitrogen (DON), NH_4_^+^, NO_3_^−^ and PO_4_^3−^ contents. Moreover, N‐cycling gene expression correlated with PO_4_^3−^ contents and with the activity of laccases. These findings highlight linkages between microbial C, N, and P cycling because of stoichiometric constraints under drought. 24 h after rewetting, we found a shift in microbial expression of C usage genes towards more labile compounds, and an increase in genes related to anabolic activity and signaling, but no signatures of stress responses, suggesting that the microbial community had overcome rewetting‐induced changes in osmotic pressure and allocated metabolic activity to growth. Overall, we show that microbial physiological drought responses and microbial resource usage related to C:N:P stoichiometry are key mechanisms of C and N cycling in the Arctic soil under drying and rewetting.

## Graphical abstract

To understand responses of Arctic microbial carbon and nitrogen cycling to climate change, we simulated a drought event in a laboratory experiment and tracked microbial gene expression and greenhouse gas emissions across increasing levels of drought. Maximum changes in gene expression, characterized by acclimation of microbial physiology to drought, occurred at intermediate drought levels. These physiological acclimations mark a tipping point where microbial communities enter a drought state with reduced activity. Crossing this tipping point is expected to be a crucial determinant of climate change responses of microbial carbon and nitrogen cycling in the future.

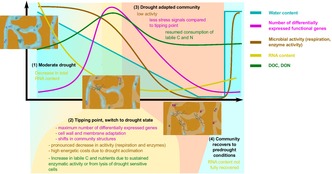

## 1 Introduction

Climate change leads to higher temperatures and alterations in the global water cycle. The Arctic is warming at a particularly high rate, and droughts will become more frequent and more intense in the future in this region (IPCC 2021). Microorganisms are important ecosystem engineers mediating soil carbon (C) and nutrient cycling as well as GHG balances. Water availability is a crucial factor controlling soil microbial activity and thus microbial feedback to climate change. Microbial activity typically decreases during drought; however, rewetting of dry soils triggers a pulse of respiration known as the Birch effect (Birch 1958). Consequently, the drying and rewetting of soils significantly affect ecosystem C fluxes, and the microbial mechanisms driving these processes remain poorly understood. Research in this field has primarily focused on arid soils, whereas very little is known about the effects of drought on soil microorganisms in the Arctic and their linkages with C and N cycling. This knowledge gap hampers our ability to project rates of microbial degradation of the enormous stock of organic C and N in Arctic soils under future climate (Palmtag et al. 2022).

The effect of water availability on microbial activity, including respiration, growth rates, and enzyme activities, has been studied extensively under both field and laboratory settings. Generally, microbial respiration and growth rates seem not to be limited by water availability at soil water contents > 25% of the soil water holding capacity (WHC) and decrease with decreasing water content, approaching zero below 10% (de Nijs et al. 2019; Leizeaga et al. 2021; Meisner et al. 2017). Despite stressful conditions under desiccation, metabolically active microbial cells have been reported under < 2% water content, however (Schulze‐Makuch et al. 2018). Extracellular enzymes function at lower water activity than microbial cells. Consequently, enzymatic degradation of soil organic matter may continue under desiccation even when a substantial part of microbial populations becomes dormant (Geisseler et al. 2011).

Although microbial activity consistently shows a marked decline under drought (Manzoni et al. 2012), microbial community structures may exhibit greater resistance. For instance, bacterial and fungal community structures did not differ between Mediterranean soils across a summer season with and without drought (Barnard et al. 2015). Sensitivity of both microbial activity and community structures to drying and rewetting has been shown to depend on the historical moisture regime (Blazewicz et al. 2014; Göransson et al. 2013; Meisner et al. 2015, 2017). Although changes in community structures correlate with enzyme activities and respiration (Barnard et al. 2015; Ochoa‐Hueso et al. 2018), the changes do not necessarily imply altered function. For instance, Canarini et al. (2021) reported increasingly divergent community structures with increasing duration of drought, whereas the amount and stoichiometry of microbial biomass as well as total enzyme activities showed resilience and converged toward pre‐drought conditions over time.

Drought affects microbial physiology by causing osmotic stress associated with damage to biomolecules, including cell walls, membranes, enzymes, and DNA (Lebre et al. 2017). Microbial mechanisms to cope with drought‐induced stress include changes in the architecture of structural compounds, repair of damaged biomolecules, or entering dormancy (Brown et al. 2000; Jones and Lennon 2010; Lebre et al. 2017). Moreover, microorganisms may increase their intracellular osmolarity by producing osmolytes, thus compensating for the increased osmolarity of the soil solution because of drought (Killham and Firestone 1984; Santos and Da Costa 2002). In addition to causing osmotic stress, drought limits microbial access to C and nutrients through diffusion, mass transport, and cellular movement as the soil pore space becomes disconnected, affecting microbial activity and C cycling (Manzoni et al. 2012). Such substrate limitation may be a stronger driver of microbial activity under desiccation than direct effects on microbial physiology (Manzoni et al. 2012). Because fungal hyphae can reach C sources and nutrients despite the water film in the soil pore space becoming disconnected under drought, fungi are thought to be more drought resistant than bacteria, and accordingly, the fungi:bacteria ratio has been found to increase under drought on the basis of both microbial biomass and DNA‐based quantification (Evans and Wallenstein 2012; Preece et al. 2019). Similarly, the bacterial Actinomycetota phylum, capable of filamentous growth, increased in abundance under desiccation (Bei et al. 2023). Moreover, the thick cell walls of gram‐positive bacteria confer drought resistance to gram‐positive taxa such as Firmicutes, and genes encoding their cell wall components increased in abundance and/or expression under drought (Bei et al. 2023; Hartmann et al. 2017; Veach and Zeglin 2020; Xu et al. 2018). Among the few studies that applied metagenome or metatranscriptome sequencing to assess microbial drought responses, some reported an increase in gene abundance and/or expression of genes encoding osmolytes (Bei et al. 2023; Malik et al. 2020; Roy Chowdhury et al. 2019; Xu et al. 2021) as well as genes related to general stress response (Bei et al. 2023; Malik et al. 2020) and sporulation (Bei et al. 2023; Hartmann et al. 2017). Moreover, C‐cycling genes, particularly those related to the degradation of labile C sources, have been reported to increase under drought, whereas N‐ and P‐cycling genes decreased (Bei et al. 2023; Hartmann et al. 2017), indicating changes in microbial resource usage in response to altered water availability. Previous metagenome‐ and metatranscriptome‐based studies were conducted in field‐based climate manipulation experiments with one or a few sampling time points and drought levels (Bei et al. 2023; Hartmann et al. 2017; Malik et al. 2020). However, a detailed understanding of how the intensity of drying affects microbial gene expression is lacking. Moreover, functional gene abundances and particularly gene expression have not been linked to microbial activity and GHG fluxes during drought, and Arctic soils represent a particularly understudied environment.

To understand Arctic C and N cycling under future drought events, in a microcosm experiment with a Greenlandic soil, we investigated community‐level microbial gene expression over a period of increasing drought followed by rewetting. We linked changes in gene expression with soil C and nutrient availability, extracellular enzyme activity as well as emissions of CO_2_, CH_4_, and N_2_O. We hypothesized that (i) the most intense drought (15% water content) causes the strongest change in microbial physiology and thus gene expression with a peak in the expression of stress response genes, (ii) the transcription of extracellular enzymes declines more rapidly than enzyme activities under drought, (iii) C and nutrient cycling rates decrease with decreasing water availability, and (iv) microbial activity is resilient to the drought event, with rates after rewetting returning to pre‐drought rates.

## 2 Materials and Methods

### 2.1 Soil Sampling

In August 2018, we sampled an Arctic tundra soil at Blæsedalen Valley on Disko Island, Greenland (N 69.2657, W 53.4706). The mean annual temperature is −3°C, and the annual range is −14°C–18°C, with a snow‐free period typically allowing plant growth from the beginning of June to the end of September. The mean annual precipitation is 410 mm. The site is located in the continuous permafrost zone, with the permafrost table at 2–3 m depth. The vegetation consists of a dry‐mesic shrub‐heath dominated by low shrubs (height < 10 cm), such as deciduous ( *Betula nana* and *Salix glauca* ) and evergreen (*Cassiope tetragona, Vaccinium vitis‐idaea*, and *Empetrum nigrum* ) plants, with a mixture of lichens covering the ground. A total of 5 kg of humic surface layer (0–5 cm) soil was sampled with a small shovel. The soil was sieved (4‐mm) and stored at 4°C until the beginning of the experiment in October 2018.

### 2.2 Drought Experiment

To simulate a summer drought, we conducted a drying experiment over 17 days with a subsequent rewetting event at room temperature (20°C). This temperature corresponds to the temperature at 2–5 cm soil depth during dry and warm weather events during July and August at the sampling site (Figure S1) and in dry years, soil water content at 5 cm depth can decrease dramatically over 2–3‐week periods during summer (Figure S2). The water content at the beginning of the experiment was 180% of the dry weight. We evenly spread 2 × 15 g of homogenized, field‐moist soil per replicate sample in open Petri dishes (1.2 cm in height, 5.4 cm in diameter). This resulted in ~1 cm soil depth, allowing for even desiccation of the soil, which was initiated following a 1‐week stabilization phase with no evaporation. Evaporation rates and water content at complete desiccation were determined in a pilot experiment on the basis of weight loss. We aimed for an even reduction in water content by 30% per time point (T) over the course of 3–4 days, until reaching maximum desiccation at 18% water content at T5. Water content was monitored gravimetrically every 1–2 days. To control evaporation rates, the Petri dishes were placed in a sterile box with a lid, which could be gradually opened or closed if evaporation rates were too high or too low. The box was placed in the laboratory at room temperature. The experiment was conducted with four replicates (two petri dishes per replicate), which were harvested destructively at each timepoint, pooling and homogenizing the two petri dishes for each replicate. After the end of the drought period, the remaining samples were rewetted to their initial water content and harvested 24 h later. For RNA isolation, 2 g per sample were subsampled and immediately stored at −80°C.

### 2.3 Measurement of Gas Fluxes

To quantify CO_2_, CH_4_, and N_2_O fluxes, a 5‐g subsample was placed in a 118‐mL glass bottle and sealed with a butyl rubber septum and a metal cap. 15 mL air was injected with a syringe to compensate for the subsequently extracted headspace volume. An initial 3‐mL headspace sample was taken with a syringe after 15 min, and thereafter, further samples were taken after 6, 24, and 30 h. All samples were transferred to 3‐mL Exetainer vials (Labco, Ceredigion, UK). The gas flux *f* was calculated as

$$f=\frac{c}{∆t}\times \frac{\;M\times P}{R\times T\times m\;}$$

where *c* is the gas concentration [ppm], *∆t* is the time [h], *M* is the molecular mass [g mol^−1^], *P* is the pressure [atm], *R* is the gas constant [0.8205 L atm K^−1^ mol^−1^], *T* is the temperature [K] and *m* is the mass of dry soil [g].

### 2.4 Measurement of Soil Physicochemical Parameters

To determine dissolved organic carbon (DOC), dissolved organic nitrogen (DON), ammonium (NH_4_^+^), nitrate (NO_3_^−^), and phosphate (PO_4_^3−^), 5 g of soil was extracted in 40 mL 0.1 M KCl in 50‐mL Falcon tubes on a shaker at 120 rpm for 10 min at 4°C. All extracts were filtered through N‐free Whatman GF‐D filters (Sigma‐Aldrich, St. Louis, MO, USA) and frozen at −18°C until analysis. DOC and DON concentrations were quantified with a TOC‐L (Shimadzu, Kyoto, Japan), and NH_4_^+^, NO_3_^−^ and PO_4_^3−^ concentrations were determined using a Fiastar 5000 (Foss, Hillerød, Denmark).

### 2.5 Extracellular Enzyme Activities

We quantified the activities of two extracellular enzymes involved in C‐cycling. Cellulase catalyzes the degradation of cellulose, and laccase oxidizes aromatic compounds that make up complex organic matter such as lignin. A soil slurry was prepared by shaking 5 g of soil in 45 mL of 0.9% NaCl in 50‐mL Falcon tubes for 1 h at 200 rpm at room temperature. Then, 10 mL of slurry was transferred to a 15‐mL Falcon tube and 2‐mm glass beads were added, followed by horizontal shaking at 220 rpm for 15 min. To determine laccase activities, 20 μL of soil slurry was incubated with 20 μL of 50 mM ABTS (2,2‐azino‐bis(3‐ethylbenzothiazoline‐6‐sulfonic acid)) in 160 μL of 100 mM sodium acetate buffer in 96‐well plates, shaking at 300 rpm at room temperature for 1 h. Autoclaved controls were included to account for background absorbance. Samples were centrifuged at 3500 rpm for 4 min, and absorbance was measured at 420 nm. For cellulase activities, 200 μL 2% Azo‐CM‐Cellulose (Megazyme, Bray, Ireland) substrate solution in 0.2 M sodium acetate (pH 4.5) was added to 200 μL extract in 1.5‐mL tubes and shaken for 24 h at 300 rpm at 37°C. Subsequently, the substrate was precipitated with 1mL of precipitation solution (0.5 M sodium acetate·3H_2_O and 0.026 M Zn‐acetate·2H_2_O in 76% ethanol, pH 5), vortexed, and incubated at room temperature for 10 min. After centrifugation at 1000 g for 10 min, the absorbance was measured from the supernatant at 590 nm. Enzyme activities were expressed as the rate of chromogenic substrate released per hour per g dry weight (dwt) soil (nmol g^−1^ dwt h^−1^).

### 2.6 RNA Isolation, cDNA Synthesis, and Sequencing

RNA was isolated from 1 g of soil per sample using the RNeasy PowerSoil Total RNA Kit (Qiagen, Hilden, Germany) according to the manufacturer's instructions. DNA was removed from the isolated RNA using the DNaseMax Kit (Qiagen, Hilden, Germany) with an extension of the DNase reaction to 1 h to ensure that all DNA was removed. PCR amplification using 16S rDNA‐specific primers (Meisner et al. 2021) of four randomly selected RNA samples was negative, as assessed by agarose gel electrophoresis, confirming that the RNA was free of DNA contamination. RNA concentrations were determined using the Qubit RNA HS Assay Kit (Invitrogen, Waltham, MA, US) according to the manufacturer's instructions. Moreover, we evaluated RNA quality using a bioanalyzer (Agilent, Santa Clara, CA, US) with the Agilent RNA 6000 Nano Kit according to the manufacturer's instructions.

Strand‐specific sequencing libraries were prepared using the NEBNext Ultra II Directional RNA Library Prep Kit for Illumina (New England Biolabs, Ipswich, MA, US) according to the manufacturer's instructions, with the modification of adding 2.5 μL of the i5 and i7 primers. Adapter ligation was performed with a five‐fold dilution of the RNA, and final PCR amplification was performed with six cycles. The quality of the cDNA libraries was verified on a bioanalyzer (Agilent, Santa Clara, CA, US) using the Agilent High Sensitivity DNA Kit according to the manufacturer's instructions. cDNA concentrations were determined with the Qubit DNA HS Assay Kit (Invitrogen, Waltham, MA, US). Subsequently, samples were pooled prior to sequencing with an amount of 10 ng cDNA per sample and brought to a final volume of 100 μL. Sequencing libraries with paired‐end 150 base pair reads were generated on an Illumina NextSeq 500 platform.

### 2.7 Processing of Metatranscriptomic Sequences

For taxonomic analyses on ribosomal RNA, raw paired‐end reads were quality‐filtered and Illumina adapters were removed using adapterremoval v2.1.3 (Lindgreen 2012) with default settings (read < 30 Phred score discarded, and minimum length was set to 30 bp). Given the substantial fraction of rRNA reads in a total RNA pool, we used the quality‐filtered and trimmed reads directly for taxonomic annotation by identifying the closest matching microorganisms on the basis of ribosomal small subunit (SSU) sequences in the SILVA database v111 (Quast et al. 2013). Taxonomic assignments were performed with *Metaxa2* v2.1.3 (Bengtsson‐Palme et al. 2015) with default settings (Reliability score cutoff = 80), focusing purely on the SSU. For analyses of functional genes, adapters and poly A tails were removed with cutadapt v1.18 (‐e 0.05; Martin 2011) and reads were quality‐filtered with trimmomatic v0.39 (TRAILING:20 SLIDINGWINDOW:4:15 AVGQUAL:20 MINLEN:40; (Bolger et al. 2014)). Reads were sorted into rRNA and mRNA sequences using sortmerna v4.2.0 (‐‐paired_in; Kopylova et al. 2012) with the SILVA v138 database (Quast et al. 2013). Assembly, creation of count tables, and annotation of contigs were conducted following the CoMW pipeline v1.0.0 (Anwar et al. 2019). mRNA reads were assembled into contigs with the script *assemble_reads.py* (‐s RF), using the Trinity assembler v2.13.2 (Grabherr et al. 2011) and the quality of the contigs was assessed with metaquast v5.0.2 (Mikheenko et al. 2016). An additional filtering step to remove rRNA contigs was carried out using *filter_ncRNA.py* (‐*e* 3), which uses Infernal (Nawrocki et al. 2009) to align against the RFam database (Kalvari et al. 2018). The reads were mapped to the contigs using the script map_reads_to_contigs.py, creating abundance tables for contigs across samples. Next, contigs were aligned against the CAZy database (Cantarel et al. 2008) to assess C‐cycling genes, the NCyc database (Tu et al. 2018) for N‐cycling genes, and the m5nr database (Wilke et al. 2012) for general functions using *align_contigs_to_database.py* (‐n F), which is based on the SWORD aligner (Vaser et al. 2016). The databases were installed as part of the CoMW suite. Since our reads were strand‐specific, we conducted this step on the basis of the three possible open reading frames in the forward direction. Annotations were filtered for an *e*‐value < 10^−5^ and only the best hit was retained for each contig using *parse_sword.py*. Then, *map_orthologs_to_count_table.py* was used to aggregate counts for contigs mapping to the same annotation, and *annotate_count_table.py* was used to create a table with hierarchical categories for each database. Moreover, we mapped the m5nr annotations to the SEED database (Overbeek et al. 2014) obtained via the MGRAST API (http://api.mg‐rast.org/m5nr/md5). Raw sequences were deposited in the European Nucleotide Archive under the accession numbers PRJEB90283.

### 2.8 Data Analysis

All data analysis was done in R (R Core Team 2022), and all plots were generated with the *ggplot* package (Wickham 2009). A markdown with code and output for all data analysis is available on figshare (https://doi.org/10.6084/m9.figshare.30085120). We assessed if soil properties, gas fluxes, RNA content, and enzyme activities changed across treatments using linear models with the *lm* function. Normality and homoskedasticity were evaluated using diagnostic plots, and data were log, square root, or cube root transformed to meet these assumptions if necessary. Post hoc tests were conducted using the function *HSD.test* in the *agricolae* package (de Mendiburu 2023). Changes in gene expression structures were assessed by principal coordinate analyses on relative abundances using the function *plot_ordination* in the *phyloseq* package (McMurdie and Holmes 2013) on the basis of the genes annotated in CAZy, NCyc, and SEED databases, respectively, aggregated at the lowest level for each database. In addition, we conducted these analyses with inferred absolute abundances by normalizing the relative abundances to the total RNA content per g dry weight soil, weighted by the fraction of mRNA reads, to which we refer as RNA‐normalized abundances, analogous to Söllinger et al. (2018). Statistical significance in differences in functional gene expression structures was evaluated by permutational multivariate analysis of variance (PERMANOVA) using the function *adonis2* in the *vegan* package (Oksanen et al. 2022) with 9999 permutations. Furthermore, we determined relationships between functional gene expression structures and soil properties as well as microbial activities using the *envfit* function in *vegan* with 9999 permutations. Differential gene expression under drought and rewetting treatments relative to the control was assessed with the *DESeq* function in the *DESeq 2* package (Love et al. 2014) using Wald tests. To this end, contigs were aggregated within categories for each database (CAZy families, SEED level 4 categories, NCyc genes). Log2fold changes were shrunk using the apeglm algorithm (Zhu et al. 2019). Since we used *p*‐values corrected for multiple testing and to reduce computational resources, we only included SEED categories that were of interest in the DESeq analysis. These were the level 1 categories “Respiration”, “Protein Metabolism”, “RNA Metabolism”, “DNA Metabolism”, “Stress Response”, “Membrane Transport”, “Amino Acids and Derivatives”, “Motility and Chemotaxis”, “Regulation and Cell signaling”, “Nucleosides and Nucleotides”, “Cell Wall and Capsule”, “Phosphorus Metabolism”, “Cell Division and Cell Cycle,” and “Dormancy and Sporulation”. *DESeq* takes a matrix of integer counts as input and calculates a size factor to account for different sequencing depths across samples, which is then included in the model as an offset. To run *DESeq* on RNA‐normalized abundances, we calculated the size factors with the *estimateSizeFactors* function and normalized them to RNA contents as described above. We then ran the *DESeq* function with these pre‐calculated size factors weighted by RNA content. Finally, we assessed the relationship between changes in gene expression on the basis of relative abundances and soil properties as well as microbial activities. To this end, we used the *dream* function from the *variancePartition* package (Hoffman and Roussos 2021; Hoffman and Schadt 2016), which accommodates the over‐dispersion typical for RNA‐seq count data and at the same time allows for the inclusion of a random effect. Thus, including treatment as a random effect, we accounted for the non‐independence of replicates. Prior to the analysis, we filtered gene categories to have > 0.1 counts per million in at least five samples and then used *calcNormFactors* from *edgeR* (Robinson et al. 2010) to account for different sequencing depths across samples. As for DESeq, we only included categories of interest for SEED to reduce the multiple testing burden.

## 3 Results

### 3.1 Soil and Microbial Properties Under Drying and Rewetting

**FIGURE 1:**
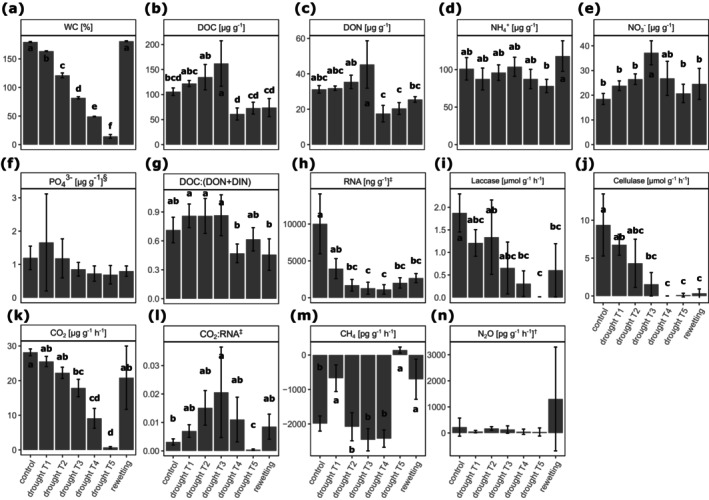
(a) Water content, (b) dissolved organic carbon, (c) dissolved organic nitrogen, (d) ammonium, (e) nitrate, (f) phosphate, (g) ratio of microbe‐available C:N, (h) RNA content, (i) laccase activity, (j) cellulase activity, (k) CO_2_ fluxes, (l) CO_2_:RNA ratio, (m) CH_4_ fluxes and (n) N_2_O fluxes over 17 days of drought followed by rewetting. Error bars represent standard deviations. Differences between treatments were assessed by linear models followed by a Tukey HSD test. Different letters indicate significant differences (*p* < 0.05). Variables were transformed to meet normality and homoskedasticity assumptions if necessary. §, square root transformation; ‡, log transformation; †, cubic root transformation.

We were able to tightly control water content across replicates and time points, which gradually decreased from 180% moisture field conditions to 18% at the end of the drought period (Figure 1a). DOC was higher at drought T3 compared to the control and lower at drought T4, T5, and under rewetting (Figure 1b). DON showed a similar pattern, but the differences between drought T3 and control, as well as between rewetting and control, were not significant (Figure 1c). NH_4_^+^ and PO_4_^3−^ did not show a clear pattern across the drought experiment (Figure 1d,f). NO_3_^−^ increased up to drought T3, where it was significantly higher than under control conditions, and then returned to control levels under further drought treatments and rewetting (Figure 1e). Furthermore, we calculated the ratio of microbe‐available C:N, as DOC:(DON+DIN), where DIN (dissolved inorganic nitrogen) is NH_4_^+^ and NO_3_^−^. The DOC:(DON+DIN) ratio was slightly lower at drought T4 and rewetting compared to drought T1–T3 (Figure 1g). RNA concentrations strongly decreased up to drought T4 and slightly increased again under drought T5 and rewetting but did not return to control levels (Figure 1h). Both laccase and cellulase activities decreased gradually with drought treatments, reaching zero under drought T4 and T5 (Figure 1i,j). Laccase activities partially recovered under rewetting, whereas cellulase activities did not. CO_2_ emissions followed the pattern of water content, reaching almost zero under drought T5 and returning to control levels under rewetting (Figure 1k). The ratio of CO_2_ to RNA increased up to drought T3, where it was significantly higher than in the control, decreased below control levels at drought T5, and increased again under rewetting (Figure 1l). Methane fluxes were negative under all treatments except drought T5 and were higher (less negative) at drought T1, T5, and rewetting compared to the other treatments (Figure 1m). N_2_O emissions did not change across the experiment, possibly because values were close to the detection limit (Figure 1n).

### 3.2 Description of the Metatranscriptome Dataset and Alphadiversity

We obtained a total of 209,178,347 high‐quality paired‐end reads (7,470,655 ± 4,177,785 per sample) of which 19,565,986 were classified as mRNA (698,785 ± 40,401 per sample; Table S1). From the mRNA reads, we assembled 10,678 contigs > 1000 base pairs (bp). The longest contig was 8205 bp, and the N50 was 660. We were able to map 44% ± 14% of all mRNA reads per sample on a contig. 94,284 reads (3626 ± 1828 per sample) mapped on contigs that could be annotated with the SEED database, 397,395 reads (15,284 ± 6746 per sample) on contigs annotated with the CAZy database, and 123,656 reads (4756 ± 2127 per sample) on contigs annotated with the NCyc database. The observed richness of functional genes annotated with the three databases did not change at any of the treatments (Figure S3). The number of prokaryotic taxa slightly decreased at drought T3, whereas the number of eukaryotic taxa slightly decreased at drought T4.

### 3.3 Functional Gene Expression Structures and Taxonomic Community Structures

**FIGURE 2:**
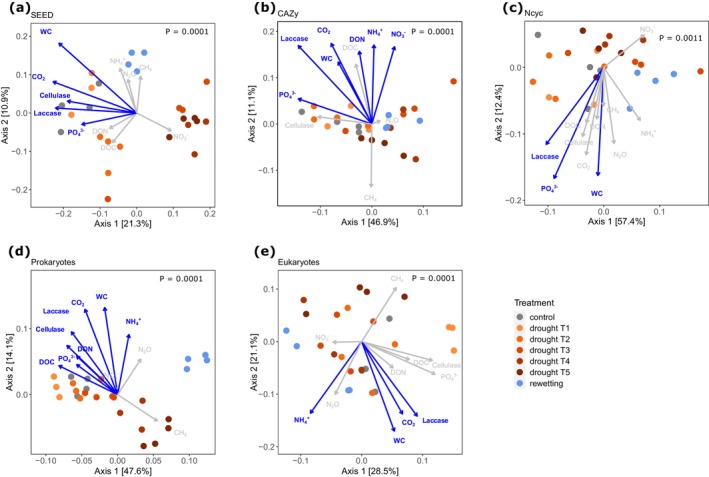
Functional gene expression structures for genes annotated with SEED (a), CAZy (b), and NCyc (c) databases and community structures of potentially active Prokaryotes (d) and Eukaryotes (e). Principal coordinate analysis on the basis of Bray–Curtis dissimilarities of relative abundances. Numbers in brackets show the percentage of variation explained by each axis. Vectors represent correlations of edaphic and microbial parameters with the ordination axes. Significance was assessed by a permutational test with 9999 permutations. Significant variables are shown in blue. *p*‐values indicate significance of differences in functional gene structures across treatments, assessed by permutational multivariate analysis of variance with 9999 permutations.

Functional gene expression structures showed the most distinct clustering across treatments for genes annotated with SEED. The first and second PCoA axes explained 21.3% and 10.9% of the variation, respectively. SEED gene expression structures shifted gradually with decreasing water content, with distinct clusters for drought T2, drought T3, T4, and T5, and rewetting, respectively (Figure 2a). Under rewetting, gene expression structures returned towards control conditions but did not fully converge. Shifts in expression structures of SEED genes were correlated with water and PO_4_^3−^ content, with cellulase and laccase activities, and with CO_2_ emissions (Figure 2a). Expression structures of CAZy‐annotated genes also gradually shifted with drought (Figure 2b) but showed more overlap between treatments compared to SEED‐annotated genes. The first two PCoA axes explained 46.9% and 11.1% of the variation, respectively. Moreover, rewetted soils did not as clearly return towards control conditions for C‐cycling genes as for SEED genes. Changes in expression structures of CAZy genes were correlated with water content, with CO_2_ emissions, with PO_4_^3−^, DON, NH_4_^+^, and NO_3_^−^ contents, and with laccase activities (Figure 2b). N‐cycling genes showed distinct structures across drought treatments, with a somewhat gradual succession with increasing drought but no clear return towards control under rewetting (Figure 2c). The first two PCoA axes explained 57.4% and 11.4% of the variation, respectively. The expression of N‐cycling genes was correlated with water content and PO_4_^3−^ contents as well as laccase activity (Figure 2c).

Because total RNA contents strongly decreased in all drought treatments and did not recover under rewetting, we also normalized the relative abundances of genes annotated with each database to the total amount of RNA, weighted by the fraction of reads that were classified as mRNA. Normalized gene expression structures for all databases followed the pattern of RNA content across treatments, with a shift away from control for all drought treatments that was partially reversed under rewetting (Figure S4).

Community structures of potentially active Prokaryotes showed moderate shifts up to drought T3 that became more pronounced at drought T4 and T5 and did not recover under rewetting (Figure 2d). Potentially active eukaryotic community structures showed significant shifts that exhibited a less clear pattern, however (Figure 2e). For Prokaryotes, the first two PCoA axes explained 47.6% and 14.1% of the variation, respectively; for Eukaryotes, the first two axes explained 28.5% and 21.1% of the variation.

### 3.4 Differential Expression in Functional Genes and Taxa

**FIGURE 3:**
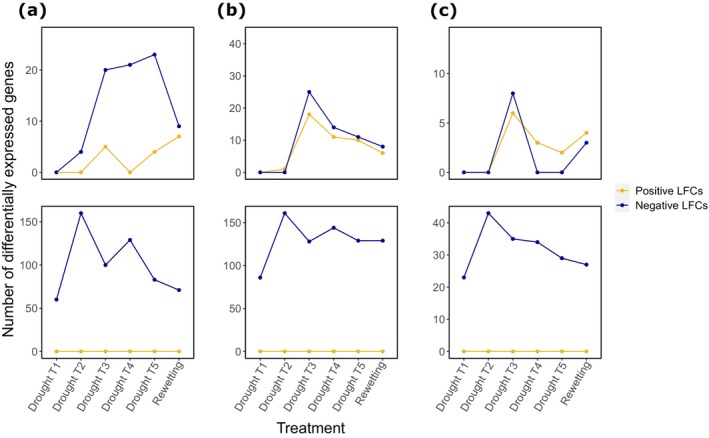
Number of differentially expressed genes relative to the control. Genes annotated with SEED (a), CAZy (b), and NCyc (c) databases on the basis of relative abundances (top row) and abundances normalized by the total RNA content weighted by the fraction of mRNA reads. Only genes with a significant log2‐fold change (LFC, *p*_adj_. < 0.05) are shown.

Next, we assessed differentially expressed genes across drought treatments relative to the control. Most SEED genes decreased in expression across the drought treatments. At drought T1, no genes were significantly differentially expressed, and at drought T2, four genes decreased in expression (Figure 3a). From drought T2–T5, 21–27 genes were differentially expressed, of which the majority decreased relative to the control. Under rewetting, the number of differentially expressed genes returned to 16, of which seven were increased. For both CAZy and NCyc genes, almost no differentially expressed genes were found at drought T1 and T2, whereas a peak in the number of differentially expressed genes was found at drought T3 (Figure 3b,c). For genes annotated with both databases, the number of increased compared to decreased genes was similar across all treatments. For NCyc genes, the number of differentially expressed genes increased under rewetting compared to drought T4 and T5. When we normalized relative abundances to total RNA content weighted by the fraction of mRNA reads, we only found downregulated genes for all databases. Interestingly, opposed to relative abundances, we found a considerable number of differentially expressed genes already at drought T1 and a peak at drought T2. These findings indicate that, particularly under drought T1 and T2, differential expression on the basis of absolute abundances was mostly driven by a decrease in total mRNA. Opposed to functional genes, the number of phyla with differential potential activity increased with increasing drought intensity from drought T3, with an even stronger increase under rewetting for both Prokaryotes and Eukaryotes (Figure S5). When normalizing to total rRNA content (RNA content weighted by the fraction of rRNA reads), the number of phyla with differential potential activity increased until drought T2 and then remained constant for all further treatments for both Prokaryotes and Eukaryotes. As for functional genes, all significant changes were negative.

**FIGURE 4:**
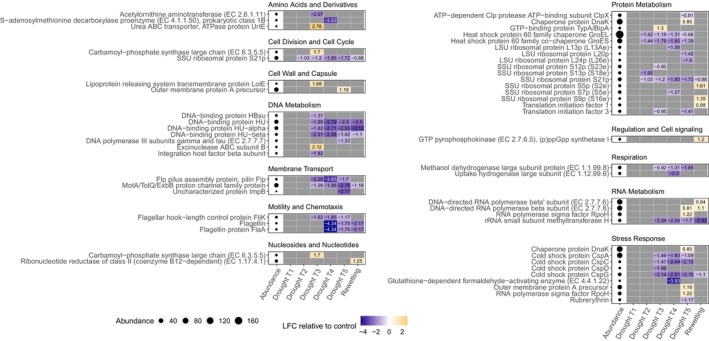
Differentially expressed genes annotated with the SEED database. Log2‐fold changes (LFC) relative to the control are shown for genes of which the abundance significantly differed from the control (*p*_adj_. < 0.05) in at least one treatment. Grey cells indicate treatments where gene expression was not different from the control. Abundance indicates read counts in the entire dataset, normalized with DESeq2.

**FIGURE 5:**
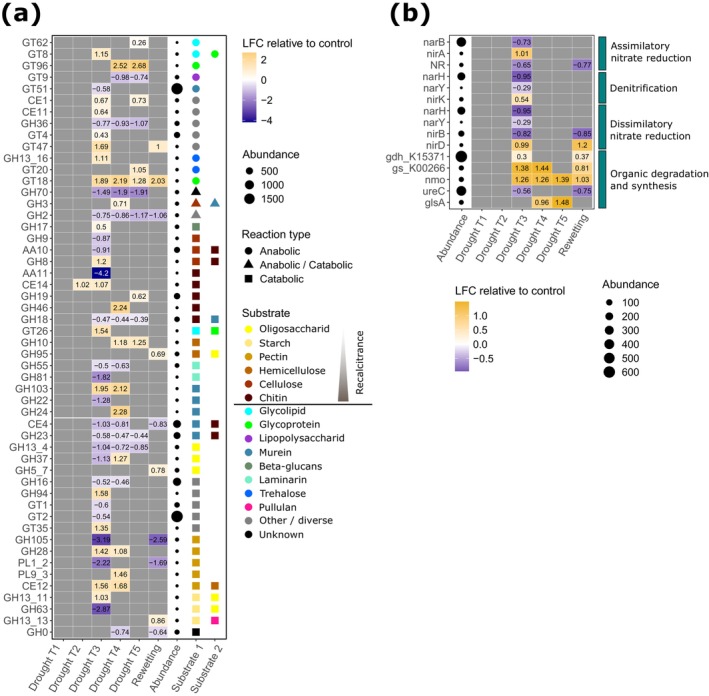
Differentially expressed genes annotated with CAZy (a) and NCyc (b) databases. Log2‐fold changes (LFC) relative to the control are shown for genes of which the abundance significantly differed from the control (*p*_adj_. < 0.05) in at least one treatment. Gray cells indicate treatments where gene expression was not different from the control. Abundance indicates read counts in the entire dataset, normalized with DESeq2. Substrate categories for CAZy families were assigned manually and sorted by recalcitrance if applicable. Two categories were assigned to account for families that act on more than one substrate category. “Other/diverse” represent families that either act on a diverse range of substrates or for which the substrate does not fit any of these categories. CBM families were omitted. N‐cycling genes that belong to more than one category are shown multiple times.

Among SEED genes, transcripts for ribosomal proteins and translation initiation factors (category protein metabolism), transcripts for several DNA‐binding proteins (category DNA metabolism) as well as transcripts for genes involved in respiration decreased from drought T2 to T5, indicating reduced biosynthetic activity as drought progressed (Figure 4). Some transcripts for ribosomal proteins were conversely enriched under rewetting, suggesting the metabolic activities were resumed. Genes involved in motility decreased from drought T3 and did not recover under rewetting. Within the category stress response, cold shock genes decreased under drought. Moreover, the heat shock proteins *GroEL* and *GroES* decreased from drought T2–T5. Upregulated stress response genes were only found at the time point with the strongest drought T5. These genes were chaperone protein *DnaK*, outer membrane protein A precursor, and RNA polymerase sigma factor *RpoH*. In addition, within amino acids and derivatives, the urea ABC transporter *UrtE* was enriched at drought T3, where DON and NO_3_^−^ peaked. Among CAZy genes, for most C substrates, we found both increasing and decreasing CAZy families acting on them under drought (Figure 5a). An exception was enzyme families that degrade hemicellulose (glycoside hydrolases [GH] 10 and 95), which increased at drought T3 and T4 and rewetting, respectively. On the basis of relative abundances, we did not find any differentially expressed CAZy families involved in lignin degradation, despite changes in laccase activity. However, we did find a decrease in the lignin‐degrading CAZy families AA1 and AA2 under all drought treatments as well as under rewetting when normalizing to RNA content. Moreover, on the basis of relative abundances, we found an increase in families that act on glycolipids and glycoproteins (glycosyltransferases [GT] 8, 9, 26, and 62) at drought T3, T4, and T5. In addition, we observed an increase in CAZy families involved in the synthesis of trehalose (GH13_16 and GT20) at drought T3 and T5, respectively.

For N cycling genes, at drought T3, we found an increase in *nirA* (assimilatory nitrate reduction), *nirK* (denitrification), *nirD* (dissimilatory nitrate reduction) as well as in *gdh*, *gs*, and *nmo* (organic degradation and synthesis) (Figure 5b). Conversely, we found a decrease in *narB* and *NR* (assimilatory nitrate reduction), in *narH* and *narY* (denitrification; dissimilatory nitrate reduction), in *nirB* (dissimilatory nitrate reduction), and in *ureC* (organic degradation and synthesis). At drought T4 and T5, we found an increase in *gs* (T4 only), *nmo*, and *glsA* (organic degradation and synthesis). Changes in the expression of N‐cycling genes under rewetting showed an intermediate pattern between the changes at T3 compared to T4 and T5, indicating partial resilience.

For Prokaryotes, we found an increase in the relative abundance of potentially active Actinomycetota and Candidate division TM7 from drought T3 (Figure S6). Conversely, the relative abundance of potentially active Nitrospirae and Gemmatimonadetes decreased with increasing drought. At drought T4, in addition, Euryoarchaeota strongly increased in relative abundance. For Eukaryotes, we found an increase in the relative abundance of Dinoflagellata, Apicomplexa, and Filastera under drought. Under rewetting, we found a decline in the abundance of Euglenozoa, Kathablepharidae, Hacrobia, and Arthropoda. Moreover, we found an increase in Streptophyta, basal Fungi, and Metamonada.

### 3.5 Relationship Between Functional Gene Expression, Edaphic Properties, and Microbial Activities

Next, we assessed how functional gene expression was related to soil properties, enzyme activities, and greenhouse gas fluxes. For SEED genes, the most significant relationships were found with water content, indicating that the drought treatment had more direct effects on the metatranscriptome than indirect effects via changes in C and nutrient availability (Table 1). For instance, we observed an increase in the expression of several genes involved in specific processes within amino acid and protein metabolism with decreasing water content (2‐amino‐3‐ketobutyrate coenzyme A ligase, Indolepyruvate ferredoxin oxidoreductase, Aspartate ammonia‐lyase, Arginyl‐tRNA synthetase, putative periplasmic protein kinase *ArgK*). Conversely, the expression of genes involved in protein degradation (ATP‐dependent Clp protease) and ribosomal proteins decreased with decreasing water content. Moreover, the expression of a gene related to flagellar motility (Flagellar hook‐associated protein 3) increased with decreasing water content. Interestingly, the expression of several SEED genes was correlated with laccase activity. These included a negative correlation for Argininosuccinate lyase, Chemotaxis protein *CheC*, and the Cyclic beta‐1,2‐glucan synthase (involved in osmotic stress), and a positive correlation for DNA‐binding protein HU‐alpha. Furthermore, Cell division protein *MraZ* was correlated with RNA content, and tRNA uridine 5‐carboxymethylaminomethyl modification enzyme *GidA* was correlated with nitrate content.

**TABLE 1:** Relationships between relative abundances of functional gene transcripts and edaphic properties as well as microbial activities.

SEED									
Category (Level 1)	Gene	Water content	NO_3_^−^	PO_4_^3−^	RNA content	Laccase	Cellulase	CO_2_	CH_4_
Amino acids and derivatives	2‐Amino‐3‐ketobutyrate coenzyme A ligase (EC 2.3.1.29)	0.0021 ↘							
	Indolepyruvate ferredoxin oxidoreductase, alpha and beta subunits	0.025 ↘							
	Aspartate ammonia‐lyase (EC 4.3.1.1)	0.025 ↘							
	Argininosuccinate lyase (EC 4.3.2.1)					0.018 ↘			
	Iron binding protein IscA for iron–sulfur cluster assembly	0.030 ↘							
Cell wall and capsule	Lipid‐A‐disaccharide synthase (EC 2.4.1.182)	0.027 ↘							
DNA metabolism	DNA‐binding protein HU‐alpha					0.031 ↗			
Membrane transport	High‐affinity leucine‐specific transport system, periplasmic binding protein LivK (TC 3.A.1.4.1)	0.034 ↗							
	High‐affinity branched‐chain amino acid transport system permease protein LivH (TC 3.A.1.4.1)	0.040 ↘							
Motility and chemotaxis	Flagellar hook‐associated protein 3	0.021 ↘							
	Chemotaxis protein CheC—inhibitor of MCP methylation					0.018 ↘			
Protein metabolism	Arginyl‐tRNA synthetase (EC 6.1.1.19)	0.025 ↘							
	putative periplasmic protein kinase ArgK and related GTPases of the G3E family	0.029 ↘							
	ATP‐dependent Clp protease ATP‐binding subunit ClpX	0.029↗							
	LSU ribosomal protein L13p (L13Ae)	0.034 ↗							
RNA metabolism	tRNA uridine 5‐carboxymethylaminomethyl modification enzyme GidA		0.043 ↗						
	Cell division protein MraZ				0.00098 ↗				
Stress response (osmotic stress)	Cyclic beta‐1,2‐glucan synthase (EC 2.4.1.‐)					0.018 ↘			

CAZy									
CAZy family	Substrate	Water content	NO_3_^−^	PO_4_^3−^	RNA content	Laccase	Cellulase	CO_2_	CH_4_
GT20	Trehalose (synthesis)	< 0.0001 ↘							
GH121	Glycoprotein		0.024 ↗						
GH13_10	Trehalose (synthesis)			0.0020 ↘					
GT62	Glycolipid					0.039 ↘		0.034 ↘	
PL9_3	Pectin					0.039 ↘			
GT18	Glycoprotein						0.013 ↘		
GT96	Glycoprotein							0.034 ↘	

Ncyc									
Category	Gene	Water content	NO_3_^−^	PO_4_^3−^	RNA content	Laccase	Cellulase	CO_2_	CH_4_
Organic degradation and synthesis	nmo						0.028 ↘		
Organic degradation and synthesis	glsA							0.00090 ↘	
Others	pmoB								0.013 ↘

*Note:* Numbers represent *p*‐values, arrows indicate if the relationship was positive (↗) or negative (↘). Only significant relationships (*p*_adj_. < 0.05) are shown. Linear mixed models were fitted with treatment as a random factor, with functional gene abundances as the dependent variable and edaphic properties and microbial activities as independent variables. A separate model was fit for each independent variable.

Among C‐cycling genes, the abundance of GT20 transcripts, involved in synthesizing the osmolyte trehalose, increased with decreasing water content (Table 1). The abundance of GH13_10 transcripts, also involved in trehalose synthesis, conversely increased with decreasing phosphate content. Expression of GT62 (glycolipid metabolism) and PL9_3 (pectin degradation) correlated negatively with laccase activity, and expression of GT18 (murein metabolism) correlated negatively with cellulase activity. The abundance of families involved in glycolipid and glycoprotein metabolism (GT62 and GT96) was moreover negatively correlated with CO_2_ emissions.

For N‐cycling genes, expression of the *nmo* gene (nitronate monooxygenase, organic degradation, and synthesis) showed a negative relationship with cellulase activity. Expression of the *glsA* (glutaminase, organic degradation, and synthesis) gene was negatively correlated with CO_2_ emissions, and expression of the *pmoB* gene (particulate monooxygenase) was negatively correlated with CH_4_ emissions, that is, positively correlated with CH_4_ uptake rates (Table 1).

## 4 Discussion

### 4.1 Changes in Microbial Activity and Soil Chemistry Under Drought

In contrast to respiration, RNA content exhibited a pronounced decrease from 180% soil water content in the control to 120% water content at drought T2 and then remained at low levels throughout the remaining treatments. Thus, respiration increased relative to RNA content up to drought T3. Although RNA content may not be representative of total biomass, this potentially indicates that an increasing fraction of C‐resources becomes allocated to energy generation relative to the production of biomolecules under drought, consistent with increased maintenance costs under changing environmental conditions and stressful cues (Donhauser et al. 2020; Malik et al. 2020; van Bodegom 2007).

**FIGURE 6:**
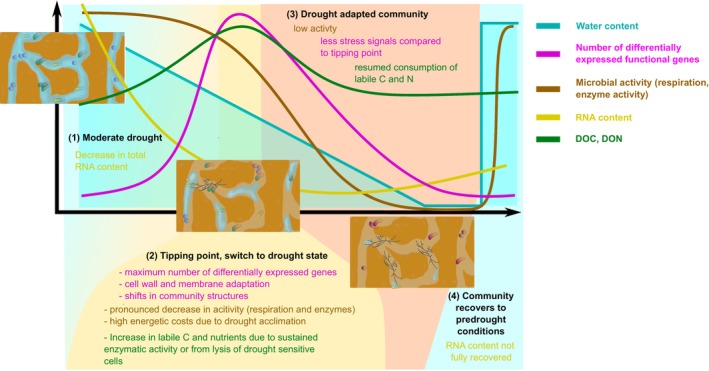
Summary of microbial drying and rewetting responses. (1) Under moderate drought, RNA content decreases, but other functions are not affected. (2) At intermediate drought, where the water film in soil pores becomes disconnected, microbial communities show maximum changes in gene expression patterns. This coincides with the onset of a pronounced decrease in microbial activity and indicates a transition of the community to the drought state involving changes in community structures and drought acclimation. (3) During a stronger drought, where almost no liquid water is left, community structures become very distinct from pre‐drought communities and overall RNA content is low but less genes are differentially expressed. This suggests that the community is now dominated by drought‐adapted taxa such as filamentous Actinobacteria, sustaining low level activity. (4) Under rewetting, most parameters show resilience and converge towards pre‐drought conditions. RNA content does not recover, however.

Previous studies documented an accumulation of soluble C and N compounds during drought (Deng et al. 2021; Schaeffer et al. 2017). Similarly, we found an increase in DOC, DON, and NO_3_^−^ until drought T3. This can be explained by reduced consumption because of reduced accessibility as the soil water film becomes disconnected and a less active microbial community, while enzymes continue to function, consistent with RNA content decreasing at less intense drought compared to enzyme activities. Moreover, it is likely that labile C and N compounds were released from dead drought‐sensitive microorganisms, contributing to the observed increase in DOC, DON, and NO_3_^−^ (Figure 6). We hypothesized that the transcription of extracellular enzymes declines more rapidly than enzyme activities under drought. In line with our hypothesis, on the basis of gene counts normalized by mRNA content, we found a decrease in the transcription of extracellular enzymes acting on lignin and cellulose (e.g., CAZy families AA1, AA2, GH8, and GH9) at milder drought treatments compared to the activity of these enzymes. This corroborates that enzymes are less drought‐sensitive than microbial cells and continue to function when their production and general microbial activity are reduced (Lawrence et al. 2009). Moreover, microbial compounds may have been released from the lysis of drought‐sensitive microorganisms (Bottner 1985; Turner et al. 2003). In contrast to a previous study under field conditions (Schaeffer et al. 2017), DOC and DON contents returned to control levels as drought progressed, further suggesting resumed microbial consumption and/or less input from lysed cells. In line with this notion, under the two strongest drought treatments, we found an increase in the abundance of actinomycetotal transcripts, which are capable of filamentous growth and therefore depend less on the transport of resources through a connected water film (Figure 6).

Soil methane fluxes were negative throughout treatments, but interestingly, soil methane uptake was lower at drought T1 and particularly T5 as well as rewetting, which was correlated with increased relative expression of *pmo* (methanotrophy). As the production of methane mostly occurs under anoxic conditions, drought is expected to lead to decreased methane production as the soil becomes more oxic (Wang et al. 2023). Rates of methane diffusion in the soil increase as soil water content decreases, which should lead to enhanced methane uptake rates. However, it is likely that this effect is partly offset by lowered methanotrophic activity as the lowered soil water content increases the restraints on cellular activity. Our findings indicate that methane fluxes are more driven by the drought sensitivity of methanotrophs than methanogens.

### 4.2 Global Patterns in Gene Expression Under Drought

Our first hypothesis stated that the most intense drought causes the strongest change in microbial gene expression. In contrast to this hypothesis, for C‐ and N‐cycling genes, we found the strongest changes in gene expression at intermediate drought treatments (T3), corresponding to 80% water content, whereas milder drought treatments barely affected gene expression, and stronger drought treatments reversed gene expression patterns towards the moist control (Figure 6). Likewise, for SEED genes, a maximum change in relative gene expression was reached at drought T3 without further changes under stronger drought. These findings indicate a tipping point at which water begins to affect microbial physiology that coincides with declining respiration and cellulase activity. For respiration, this tipping point marked the start of a pronounced decrease that approaches zero under complete desiccation, in line with a sigmoidal response to water availability observed previously for respiration and microbial growth rates (de Nijs et al. 2019; Leizeaga et al. 2021; Meisner et al. 2017). Conversely, for gene expression, the tipping point marks a point of maximum change, suggesting the transition of the microbial community to an alternative metabolic state under drought.

The observed richness of functional genes and taxonomic groups remained surprisingly stable across drought treatments. At room temperature, RNA has a half‐life of several days, and thus relic RNA may have contributed to the stability of gene richness (Schostag et al. 2020). The strong decrease in RNA content and the pronounced changes in the composition of the expressed genes and taxonomic groups suggest, however, that the contribution of non‐degraded RNA was minor.

### 4.3 Stress Response Under Drought

Stress response genes, except for cold shock genes, were differentially expressed only at the strongest drought. In agreement with previous studies (Bei et al. 2023; Malik et al. 2020; Roy Chowdhury et al. 2019; Xu et al. 2021), already at intermediate drought (T3), we observed a relative increase in the expression of genes for the synthesis of the osmolyte trehalose, serving to counteract osmotic imbalances because of drought. Moreover, from drought T3 on, we found an increase in the expression of several CAZymes for the synthesis and degradation of glycolipids and glycoproteins as well as for the degradation of murein. Glycolipids and glycoproteins are membrane compounds, and their increased expression may contribute to maintaining membrane stability under osmotic stress (Mykytczuk et al. 2007) similar to previous observations under heat stress (Donhauser et al. 2021). Moreover, an increase in the expression of CAZymes for the degradation of these compounds suggests that microorganisms feed on cell compounds from dead microorganisms. For the cell wall compound murein, we found an increase in degrading but not in synthesizing CAZymes under drought. Although modifications of cell walls have been reported as a drought adaptation (Bei et al. 2023; Hartmann et al. 2017), the lack of increased gene expression for murein synthesis in our study indicates that surviving cells feed on cell walls from dead cells (Donhauser et al. 2021).

In accordance with a trade‐off between acquisition of complex resources and stress response (Malik et al. 2020), we found a negative correlation between exoenzyme activities and Cyclic beta‐1,2‐glucan synthase (osmotic stress response) as well as CAZymes acting on glycolipids and proteins. As opposed to previous studies (Hartmann et al. 2017; Malik et al. 2020), we did not observe an increase in the expression of genes associated with spore formation. Although spore formation may only occur under longer periods of drought than in our experiment, this finding, together with a similar number of active taxa across all treatments, suggests that the microbial community in the Arctic soil studied here was relatively resistant to drought. One explanation is that Arctic soils are frequently subjected to freeze–thaw cycles, inducing changes in water availability and osmolarity as well as triggering similar microbial response mechanisms compared to drought (Schimel et al. 2007). This is also reflected in the high resilience of microbial activity, that is, the CO_2_ production rates returning to near pre‐drought levels following rewetting.

### 4.4 Stoichiometry Effects on C, N, and P Cycling Under Drought

Interestingly, the expression structures of C‐cycling genes correlated more strongly with DON, NH_4_^+^, NO_3_^−^, and PO_4_^3−^ than with DOC, and the expression structures of N‐cycling genes correlated more strongly with PO_4_^3−^ than with N compounds. The C:N:P stoichiometry of microbial biomass is subject to narrow constraints coupling the assimilation of one element tightly with the availability of the other two (Xu et al. 2013). Phosphate availability is a key control of microbial growth because of the high P content of RNA and ribosomes (Elser et al. 2000). Thus, microbial use of C sources may be more constrained by N and P availability than by the C source itself. Accordingly, we did not observe a consistent shift in the expression of CAZymes toward more labile or more recalcitrant compounds during the drought, indicating that C‐source quality did not affect microbial drought responses. At drought T4, T5, and rewetting, we observed a decrease in the DOC/(DON+DIN) ratio. This could be a consequence of increased use of C for energy generation to sustain drought acclimation, which would be less affected by stoichiometric constraints than biomass production, consistent with the peak in the respiration:RNA ratio at the preceding time point (drought T3). Increased energetic demand is in accordance with the pronounced metabolic transition toward drought adaptation at intermediate drought levels, as outlined previously.

Coupling between microbial C and N cycling was further evidenced by a correlation between N‐cycling gene expression structures and laccase activities. This could be explained by the N‐mining hypothesis, stating that under N limitation, microorganisms degrade recalcitrant organic compounds such as lignin despite high energetic costs to obtain N from lignin‐shielded proteins (Moorhead and Sinsabaugh 2006). Accordingly, N addition has been shown to decrease the decomposition of recalcitrant organic matter as well as the activity of lignocellolytic enzymes such as laccase and phenol oxidase (Craine et al. 2007; Osono and Takeda 2001; Rinkes et al. 2016; Sinsabaugh et al. 2005). In line with reduced N demand, we found a decrease in the expression of N assimilation genes such as *gs*, *gdh*, and *glsA* normalized to total mRNA content across all drought treatments, driven by the decrease in total RNA content. An increase in these genes at drought T3, T4, and T5 and rewetting on a relative basis could indicate a shift in metabolism towards maintenance and drought acclimation with increased protein synthesis to sustain, for instance, the production of osmolytes as well as remodeling of membranes and cell walls. Moreover, at drought T3, where microbe‐available N compounds were highest, we found a relative decrease in urease, further supporting decreased microbial N demand. Overall, consistent with N limitation in Arctic soils (Xu et al. 2013), the links between the expression of N‐cycling genes and laccase activity support a role of laccase in N mining, highlighting the interplay between drought responses and stoichiometric demands.

### 4.5 Rewetting Responses

Upon rewetting, CO_2_ fluxes returned to control levels (Figure 6). Previous studies reported a burst of respiration upon rewetting that exceeded CO_2_ emissions under constantly moist conditions (de Nijs et al. 2019; Göransson et al. 2013; Meisner et al. 2015). The peak in respiration usually occurs between a few hours and 2 days after rewetting (Blazewicz et al. 2014; de Nijs et al. 2019; Göransson et al. 2013; Meisner et al. 2015); thus, after 24 h in this study, respiration rates may already have declined again. After a severe drought, respiration rates have been found to increase immediately upon rewetting, whereas the onset of microbial growth was subject to a lag phase, resulting in a temporarily reduced C use efficiency (Meisner et al. 2015). High respiration relative to biomass production, that is, low C use efficiency, has been attributed to microbial energy demands to adapt their metabolism to sudden changes in osmotic pressure and to resuscitate from dormancy (Blazewicz et al. 2014). Moreover, the presence of a lag phase has been shown to depend on the intensity of the drought (Meisner et al. 2017). The threshold at which desiccation intensity triggers such a lag phase may correspond to the transition to an alternative metabolic state under drought, as observed at T3 in our soil. Thus, under rewetting, growth would be delayed because of the transition from the drought metabolic state on the basis of catabolism to anabolic metabolism. Similar to drying, rewetting involves pronounced changes in osmotic pressure and can trigger a stress response (Schimel 2018). Twenty‐four hours after rewetting, we did not observe an increase in the expression of stress response genes, likely because transcriptomic responses to changes in osmotic pressure occurred earlier upon rewetting and had faded at the time of sampling. Moreover, global gene expression on the basis of SEED subsystems showed considerable resilience, indicating that the metabolic state of the microbial community was largely recovered 24 h after rewetting. It should be noted that taxonomic community structures diverged even more under rewetting than under drought, indicating that the resilience of microbial functions can be attributed to functional redundancy. Among SEED genes, we found a relative increase in the expression of ribosomal proteins, RNA polymerase, as well as in N assimilation genes compared to pre‐drought conditions, indicating a shift in microbial metabolism towards increased anabolic activity and growth.

As opposed to respiration, RNA contents had not recovered to control levels under rewetting. This suggests high mass‐specific respiration, possibly owing to metabolic adaptations to ramp up growth. The observed increase in the expression of GTP pyrophosphokinase genes after rewetting, which produces the second messenger ppGpp, regulating cellular responses upon changes in environmental conditions, supports such adaptive processes.

Among C‐cycling genes, we found an increase in the expression of genes degrading labile C substrates such as oligosaccharides and starch after rewetting, whereas the activities of laccase and cellulase, degrading recalcitrant organic matter, did not recover after rewetting. Labile C sources might become available owing to lysis of cells upon sudden changes in soil water content and thus osmotic pressure, explaining the shift of microbial C usage towards labile compounds. In addition, C substrates may accumulate under drought as microorganisms become inactive, whereas enzymes continue to function. Indeed, we found an increase in DOC and DON concentrations under intermediate drought, which, however, were reduced below control levels under high drought intensity. Thus, increased accumulation of labile C under drought did not fuel the rewetting response in our study. Overall, C‐ and N‐cycling genes showed little resilience 24 h after the end of drought, stressing the importance of substrate chemistry and resource availability under drying and rewetting for microbial physiology and thus biogeochemical cycling.

### 4.6 Effects of Drought Versus Time

In our experiment, we compared the drought treatments at different time points to the pre‐drought control. Thus, in addition to drought effects, microbial communities and therefore their functions may have experienced succession over time (Bang‐Andreasen et al. 2020). During drought T1 and T2, few significant changes both in gene expression and activity occurred, and hence succession may have contributed to the subtle changes observed. The pronounced changes in gene expression and activity observed from drought T3 onwards, conversely, can mainly be attributed to drought, whereas succession is expected to play a minor role. Previous studies have shown that at the time scales of our experiment, the effect of time was much smaller than the effect of drought for DNA‐based microbial community structures (Cordero et al. 2023) and much smaller than the effect of other similarly strong disturbances such as heat for RNA‐based microbial community structures (Donhauser et al. 2020; Jurburg et al. 2017). Similarly, in support of drought effects in our study, microbial activities such as respiration and growth rates did not change with time in constantly moist soils, but showed pronounced changes under drying and rewetting (Göransson et al. 2013). Moreover, succession of community structures with time at the RNA level typically occurs more linearly (Bang‐Andreasen et al. 2020; Jurburg et al. 2017), indicating that the pronounced non‐linear responses from drought T3 onwards are the result of drought.

## 5 Conclusion

In summary, for the first time, we evaluated microbial gene expression together with GHG fluxes, enzyme activities as well as soil C and N status across multiple levels of drought in an Arctic soil. This enabled us to precisely relate microbial drought sensitivity to soil water content. Our study highlights that gene expression changed the most at intermediate drought levels, indicating a tipping point in microbial physiology. Our findings suggest that the pronounced metabolic shifts occurring at this tipping point require substantial amounts of energy and cause microorganisms to respire soil C rather than incorporating it into biomass, potentially leading to soil C loss under drought. Thus, crossing the tipping point may be more relevant for the fate of soil C than the amplitude of a drought event. Although microbial activity was not resistant to drought, it showed high resilience, probably because a large fraction of the microorganisms survived and because of functional redundancy. Moreover, our study points out that microbial physiology as well as C, N, and P cycling under drying and rewetting are intertwined, stressing the importance of incorporating microbial mechanisms into biogeochemical models to improve predictions of soil C, N, and P cycling under future climate.

## Author Contributions

**Theis Thomsen:** conceptualization, formal analysis, investigation. **Morten Dencker Schostag:** supervision, writing – review and editing. **Anders Priemé:** conceptualization, funding acquisition, supervision, writing – review and editing. **Jonathan Donhauser:** conceptualization, formal analysis, visualization, writing – original draft.

## Conflicts of Interest

The authors declare no conflicts of interest.

## Supporting information


**Figure S1:** Temperature at 5 cm soil depth at the sampling site measured in situ with four different HOBO Temperature data loggers from 2012 to 2017.
**Figure S2:** Soil water content at 5 cm soil depth at the sampling site measured in situ with HOBO Soil Moisture data logger during the dry summer of 2019.
**Figure S3:** Observed richness for functional genes and rRNA for prokaryotes and eukaryotes. Error bars represent standard deviations. Significance was assessed by linear models followed by a Tukey HSD test.
**Figure S4:** Functional gene structures for genes annotated with SEED, CAZy and NCyc databases normalized to total mRNA content. Principal coordinate analysis on the basis of Bray–Curtis dissimilarities. Numbers in brackets show the percentage of variation explained by each axis. Vectors represent correlations of edaphic and microbial parameters with functional gene structures. Significance was assessed by a permutational test with 9999 permutations. Significant variables are shown in blue. *p*‐values indicate significance of differences in functional gene structures across treatments, assessed by permutational multivariate analysis of variance with 9999 permutations.
**Figure S5:** Number of differentially expressed phyla relative to the control. Prokaryotic and eukaryotic rRNA genes on the basis of relative abundances or abundances normalized by total mRNA contents with a significant log2‐fold change (LFC, *p*_adj_. < 0.05) are shown.
**Figure S6:** Differentially abundant potentially active prokaryotic and eukaryotic phyla. Log2‐fold changes (LFC) relative to the control are shown for phyla of which the abundance significantly differed from the control (*p*_adj_. < 0.05) in at least one treatment. Grey cells indicate treatments where abundance was not different from the control. Abundance indicates read counts in the entire dataset, normalized with Deseq2.

**Table S1:** Number of reads across the steps of the bioinformatic pipeline for processing functional genes.

**Data S1:** gcb70502‐sup‐0003‐Supinfo1.html.

**Data S2:** gcb70502‐sup‐0004‐Supinfo2.html.

## Data Availability

Raw sequences were deposited in the European Nucleotide Archive under the accession number PRJEB90283. All other data as well as code for statistical analyses are available on figshare (https://doi.org/10.6084/m9.figshare.30085120).

## References

[gcb70502-bib-0001] Anwar, M. Z. , A. Lanzen , T. Bang‐Andreasen , and C. S. Jacobsen . 2019. “To Assemble or Not to Resemble—A Validated Comparative Metatranscriptomics Workflow (CoMW).” GigaScience 8, no. 8: 1–10. 10.1093/gigascience/giz096. PMC6667343 31363751

[gcb70502-bib-0002] Bang‐Andreasen, T. , M. Z. Anwar , A. Lanzén , et al. 2020. “Total RNA Sequencing Reveals Multilevel Microbial Community Changes and Functional Responses to Wood Ash Application in Agricultural and Forest Soil.” FEMS Microbiology Ecology 96, no. 3: fiaa016. 10.1093/femsec/fiaa016. 32009159 PMC7028008

[gcb70502-bib-0003] Barnard, R. L. , C. A. Osborne , and M. K. Firestone . 2015. “Changing Precipitation Pattern Alters Soil Microbial Community Response to Wet‐Up Under a Mediterranean‐Type Climate.” ISME Journal 9: 946–957. 10.1038/ismej.2014.192. 25314319 PMC4817701

[gcb70502-bib-0004] Bei, Q. , T. Reitz , B. Schnabel , et al. 2023. “Extreme Summers Impact Cropland and Grassland Soil Microbiomes.” ISME Journal 17, no. 10: 1589–1600. 10.1038/s41396-023-01470-5. 37419993 PMC10504347

[gcb70502-bib-0005] Bengtsson‐Palme, J. , M. Hartmann , K. M. Eriksson , et al. 2015. “metaxa2: Improved Identification and Taxonomic Classification of Small and Large Subunit rRNA in Metagenomic Data.” Molecular Ecology Resources 15, no. 6: 1403–1414. 10.1111/1755-0998.12399. 25732605

[gcb70502-bib-0006] Birch, H. F. 1958. “The Effect of Soil Drying on Humus Decomposition and Nitrogen Availability.” Plant and Soil 10, no. 1: 9–31. 10.1007/BF01343734.

[gcb70502-bib-0007] Blazewicz, S. J. , E. Schwartz , M. K. Firestone , and M. K. Firestone1 . 2014. “Growth and Death of Bacteria and Fungi Underlie Rainfall‐Induced Carbon Dioxide Pulses From Seasonally Dried Soil.” Ecology 95, no. 5: 1162–1172. 25000748 10.1890/13-1031.1

[gcb70502-bib-0008] Bolger, A. M. , M. Lohse , and B. Usadel . 2014. “Trimmomatic: A Flexible Trimmer for Illumina Sequence Data.” Bioinformatics 30, no. 15: 2114–2120. 10.1093/bioinformatics/btu170. 24695404 PMC4103590

[gcb70502-bib-0009] Bottner, P. 1985. “Response of Microbial Biomass to Alternate Moist and Dry Conditions in a Soil Incubated With 14C‐ and 15N‐Labelled Plant Material.” Soil Biology and Biochemistry 17, no. 3: 329–337. 10.1016/0038-0717(85)90070-7.

[gcb70502-bib-0010] Brown, G. R. , I. C. Sutcliffe , D. Bendell , and S. P. Cummings . 2000. “The Modification of the Membrane of *Oceanomonas baumannii* T When Subjected to Both Osmotic and Organic Solvent Stress.” FEMS Microbiology Letters 189, no. 2: 149–154. 10.1111/J.1574-6968.2000.TB09221.X. 10930729

[gcb70502-bib-0011] Canarini, A. , H. Schmidt , L. Fuchslueger , et al. 2021. “Ecological Memory of Recurrent Drought Modifies Soil Processes via Changes in Soil Microbial Community.” Nature Communications 12, no. 1: 1–14. 10.1038/s41467-021-25675-4. PMC8421443 34489463

[gcb70502-bib-0012] Cantarel, B. L. I. , P. M. Coutinho , C. Rancurel , T. Bernard , V. Lombard , and B. Henrissat . 2008. “The Carbohydrate‐Active Enzymes Database (CAZy): An Expert Resource for Glycogenomics.” Nucleic Acids Research 37: D233–D238. 10.1093/nar/gkn663. 18838391 PMC2686590

[gcb70502-bib-0013] Cordero, I. , A. Leizeaga , L. C. Hicks , J. Rousk , and R. D. Bardgett . 2023. “High Intensity Perturbations Induce an Abrupt Shift in Soil Microbial State.” ISME Journal 17, no. 12: 2190–2199. 10.1038/s41396-023-01512-y. 37814127 PMC10690886

[gcb70502-bib-0014] Craine, J. M. , C. Morrow , and N. Fierer . 2007. “Microbial Nitrogen Limitation Increases Decomposition.” Ecology 88, no. 8: 2105–2113. 10.1890/06-1847.1. 17824441

[gcb70502-bib-0015] de Mendiburu, F. 2023. “agricolae: Statistical Procedures for Agricultural Research.” https://CRAN.R‐project.org/package=agricolae.

[gcb70502-bib-0016] de Nijs, E. A. , L. C. Hicks , A. Leizeaga , A. Tietema , and J. Rousk . 2019. “Soil Microbial Moisture Dependences and Responses to Drying–Rewetting: The Legacy of 18 Years Drought.” Global Change Biology 25, no. 3: 1005–1015. 10.1111/GCB.14508. 30387912

[gcb70502-bib-0017] Deng, L. , C. Peng , D.‐G. Kim , et al. 2021. “Drought Effects on Soil Carbon and Nitrogen Dynamics in Global Natural Ecosystems.” Earth‐Science Reviews 214: 103501. 10.1016/j.earscirev.2020.103501.

[gcb70502-bib-0018] Donhauser, J. , P. A. Niklaus , J. Rousk , C. Larose , and B. Frey . 2020. “Temperatures Beyond the Community Optimum Promote the Dominance of Heat‐Adapted, Fast Growing and Stress Resistant Bacteria in Alpine Soils.” Soil Biology and Biochemistry 148: 107873. 10.1016/j.soilbio.2020.107873.

[gcb70502-bib-0019] Donhauser, J. , W. Qi , B. Bergk‐Pinto , and B. Frey . 2021. “High Temperatures Enhance the Microbial Genetic Potential to Recycle C and N From Necromass in High‐Mountain Soils.” Global Change Biology 27, no. 7: 1365–1386. 10.1111/gcb.15492. 33336444

[gcb70502-bib-0020] Elser, J. J. , R. W. Sterner , E. Gorokhova , et al. 2000. “Biological Stoichiometry From Genes to Ecosystems.” Ecology Letters 3, no. 6: 540–550. 10.1111/j.1461-0248.2000.00185.x.

[gcb70502-bib-0021] Evans, S. E. , and M. D. Wallenstein . 2012. “Soil Microbial Community Response to Drying and Rewetting Stress: Does Historical Precipitation Regime Matter?” Biogeochemistry 109: 101–116. 10.1007/s10533-011-9638-3.

[gcb70502-bib-0022] Geisseler, D. , W. R. Horwath , and K. M. Scow . 2011. “Soil Moisture and Plant Residue Addition Interact in Their Effect on Extracellular Enzyme Activity.” Pedobiologia 54, no. 2: 71–78. 10.1016/j.pedobi.2010.10.001.

[gcb70502-bib-0023] Göransson, H. , D. L. Godbold , D. L. Jones , and J. Rousk . 2013. “Bacterial Growth and Respiration Responses Upon Rewetting Dry Forest Soils: Impact of Drought‐Legacy.” Soil Biology and Biochemistry 57: 477–486. 10.1016/J.SOILBIO.2012.08.031.

[gcb70502-bib-0024] Grabherr, M. G. , B. J. Haas , M. Yassour , et al. 2011. “Full‐Length Transcriptome Assembly From RNA‐Seq Data Without a Reference Genome.” Nature Biotechnology 29, no. 7: 644–652. 10.1038/nbt.1883. PMC3571712 21572440

[gcb70502-bib-0025] Hartmann, M. , I. Brunner , F. Hagedorn , et al. 2017. “A Decade of Irrigation Transforms the Soil Microbiome of a Semi‐Arid Pine Forest.” Molecular Ecology 26, no. 4: 1190–1206. 10.1111/MEC.13995. 28028891

[gcb70502-bib-0026] Hoffman, G. E. , and P. Roussos . 2021. “Dream: Powerful Differential Expression Analysis for Repeated Measures Designs.” Bioinformatics 37, no. 2: 192–201. 10.1093/BIOINFORMATICS/BTAA687. 32730587 PMC8055218

[gcb70502-bib-0027] Hoffman, G. E. , and E. E. Schadt . 2016. “variancePartition: Interpreting Drivers of Variation in Complex Gene Expression Studies.” BMC Bioinformatics 17: 483. 10.1186/s12859-016-1323-z. 27884101 PMC5123296

[gcb70502-bib-0028] IPCC . 2021. “Summary for Policymakers.” In Climate Change 2021: The Physical Science Basis. Contribution of Working Group I to the Sixth Assessment Report of the Intergovernmental Panel on Climate Change, 3–32. Cambridge University Press. 10.1017/9781009157896.001.

[gcb70502-bib-0029] Jones, S. E. , and J. T. Lennon . 2010. “Dormancy Contributes to the Maintenance of Microbial Diversity.” Proceedings of the National Academy of Sciences of the United States of America 107, no. 13: 5881–5886. 10.1073/PNAS.0912765107. 20231463 PMC2851880

[gcb70502-bib-0030] Jurburg, S. D. , I. Nunes , J. C. Stegen , et al. 2017. “Autogenic Succession and Deterministic Recovery Following Disturbance in Soil Bacterial Communities.” Scientific Reports 7: 45691. 10.1038/srep45691. 28383027 PMC5382530

[gcb70502-bib-0031] Kalvari, I. , E. P. Nawrocki , J. Argasinska , et al. 2018. “Non‐Coding RNA Analysis Using the Rfam Database.” Current Protocols in Bioinformatics 62, no. 1: e51. 10.1002/cpbi.51. 29927072 PMC6754622

[gcb70502-bib-0032] Killham, K. , and M. K. Firestone . 1984. “Salt Stress Control of Intracellular Solutes in Streptomycetes Indigenous to Saline Soils.” Applied and Environmental Microbiology 47, no. 2: 301–306. 10.1128/AEM.47.2.301-306.1984. 16346472 PMC239664

[gcb70502-bib-0033] Kopylova, E. , L. Noé , and H. Touzet . 2012. “SortMeRNA: Fast and Accurate Filtering of Ribosomal RNAs in Metatranscriptomic Data.” Bioinformatics 28, no. 24: 3211–3217. 10.1093/bioinformatics/bts611. 23071270

[gcb70502-bib-0034] Lawrence, C. R. , J. C. Neff , and J. P. Schimel . 2009. “Does Adding Microbial Mechanisms of Decomposition Improve Soil Organic Matter Models? A Comparison of Four Models Using Data From a Pulsed Rewetting Experiment.” Soil Biology and Biochemistry 41, no. 9: 1923–1934. 10.1016/J.SOILBIO.2009.06.016.

[gcb70502-bib-0035] Lebre, P. H. , P. De Maayer , and D. A. Cowan . 2017. “Xerotolerant Bacteria: Surviving Through a Dry Spell.” Nature Reviews Microbiology 15, no. 5: 285–296. 10.1038/nrmicro.2017.16. 28316329

[gcb70502-bib-0036] Leizeaga, A. , L. C. Hicks , L. Manoharan , C. V. Hawkes , and J. Rousk . 2021. “Drought Legacy Affects Microbial Community Trait Distributions Related to Moisture Along a Savannah Grassland Precipitation Gradient.” Journal of Ecology 109, no. 9: 3195–3210. 10.1111/1365-2745.13550.

[gcb70502-bib-0037] Lindgreen, S. 2012. “AdapterRemoval: Easy Cleaning of Next‐Generation Sequencing Reads.” BMC Research Notes 5, no. 1: 337. 10.1186/1756-0500-5-337. 22748135 PMC3532080

[gcb70502-bib-0038] Love, M. I. , W. Huber , and S. Anders . 2014. “Moderated Estimation of Fold Change and Dispersion for RNA‐Seq Data With DESeq2.” Genome Biology 15, no. 12: 550. 10.1186/s13059-014-0550-8. 25516281 PMC4302049

[gcb70502-bib-0039] Malik, A. A. , T. Swenson , C. Weihe , et al. 2020. “Drought and Plant Litter Chemistry Alter Microbial Gene Expression and Metabolite Production.” ISME Journal 14, no. 9: 9. 10.1038/s41396-020-0683-6. PMC7608424 32444813

[gcb70502-bib-0040] Manzoni, S. , J. P. Schimel , and A. Porporato . 2012. “Responses of Soil Microbial Communities to Water Stress: Results From a Meta‐Analysis.” Ecology 93, no. 4: 930–938. 10.1890/11-0026.1. 22690643

[gcb70502-bib-0041] Martin, M. 2011. “CUTADAPT Removes Adapter Sequences From High‐Throughput Sequencing Reads.” EMBnet.Journal 17: 10–12. 10.14806/ej.17.1.200.

[gcb70502-bib-0042] McMurdie, P. J. , and S. Holmes . 2013. “Phyloseq: An R Package for Reproducible Interactive Analysis and Graphics of Microbiome Census Data.” PLoS One 8, no. 4: e61217. 10.1371/journal.pone.0061217. 23630581 PMC3632530

[gcb70502-bib-0043] Meisner, A. , A. Leizeaga , J. Rousk , and E. Bååth . 2017. “Partial Drying Accelerates Bacterial Growth Recovery to Rewetting.” Soil Biology and Biochemistry 112: 269–276. 10.1016/J.SOILBIO.2017.05.016.

[gcb70502-bib-0044] Meisner, A. , J. Rousk , and E. Bååth . 2015. “Prolonged Drought Changes the Bacterial Growth Response to Rewetting.” Soil Biology and Biochemistry 88: 314–322. 10.1016/J.SOILBIO.2015.06.002.

[gcb70502-bib-0045] Meisner, A. , B. L. Snoek , J. Nesme , et al. 2021. “Soil Microbial Legacies Differ Following Drying‐Rewetting and Freezing‐Thawing Cycles.” ISME Journal 15, no. 4: 1207–1221. 10.1038/s41396-020-00844-3. 33408369 PMC8115648

[gcb70502-bib-0046] Mikheenko, A. , V. Saveliev , and A. Gurevich . 2016. “MetaQUAST: Evaluation of Metagenome Assemblies.” Bioinformatics 32, no. 7: 1088–1090. 10.1093/bioinformatics/btv697. 26614127

[gcb70502-bib-0047] Moorhead, D. L. , and R. L. Sinsabaugh . 2006. “A Theoretical Model of Litter Decay and Microbial Interaction.” Ecological Monographs 76, no. 2: 151–174. 10.1890/0012-9615(2006)076[0151:ATMOLD]2.0.CO;2.

[gcb70502-bib-0048] Mykytczuk, N. C. S. , J. T. Trevors , L. G. Leduc , and G. D. Ferroni . 2007. “Fluorescence Polarization in Studies of Bacterial Cytoplasmic Membrane Fluidity Under Environmental Stress.” Progress in Biophysics and Molecular Biology 95, no. 1: 60–82. 10.1016/j.pbiomolbio.2007.05.001. 17628643

[gcb70502-bib-0049] Nawrocki, E. P. , D. L. Kolbe , and S. R. Eddy . 2009. “Infernal 1.0: Inference of RNA Alignments.” Bioinformatics 25, no. 10: 1335–1337. 10.1093/bioinformatics/btp157. 19307242 PMC2732312

[gcb70502-bib-0050] Ochoa‐Hueso, R. , S. L. Collins , M. Delgado‐Baquerizo , et al. 2018. “Drought Consistently Alters the Composition of Soil Fungal and Bacterial Communities in Grasslands From Two Continents.” Global Change Biology 24, no. 7: 2818–2827. 10.1111/GCB.14113. 29505170

[gcb70502-bib-0051] Oksanen, J. , G. L. Simpson , F. G. Blanchet , et al. 2022. “vegan: Community Ecology Package.” https://CRAN.R‐project.org/package=vegan.

[gcb70502-bib-0052] Osono, T. , and H. Takeda . 2001. “Effects of Organic Chemical Quality and Mineral Nitrogen Addition on Lignin and Holocellulose Decomposition of Beech Leaf Litter by *Xylaria* sp.” European Journal of Soil Biology 37, no. 1: 17–23. 10.1016/S1164-5563(01)01066-4.

[gcb70502-bib-0053] Overbeek, R. , R. Olson , G. D. Pusch , et al. 2014. “The SEED and the Rapid Annotation of Microbial Genomes Using Subsystems Technology (RAST).” Nucleic Acids Research 42, no. D1: 206–214. 10.1093/nar/gkt1226. PMC3965101 24293654

[gcb70502-bib-0054] Palmtag, J. , J. Obu , P. Kuhry , et al. 2022. “A High Spatial Resolution Soil Carbon and Nitrogen Dataset for the Northern Permafrost Region Based on Circumpolar Land Cover Upscaling.” Earth System Science Data 14, no. 9: 4095–4110. 10.5194/essd-14-4095-2022.

[gcb70502-bib-0055] Preece, C. , E. Verbruggen , L. Liu , J. T. Weedon , and J. Peñuelas . 2019. “Effects of Past and Current Drought on the Composition and Diversity of Soil Microbial Communities.” Soil Biology and Biochemistry 131: 28–39. 10.1016/J.SOILBIO.2018.12.022.

[gcb70502-bib-0056] Quast, C. , E. Pruesse , P. Yilmaz , et al. 2013. “The SILVA Ribosomal RNA Gene Database Project: Improved Data Processing and Web‐Based Tools.” Nucleic Acids Research 41, no. Database issue: D590–D596. 10.1093/nar/gks1219. 23193283 PMC3531112

[gcb70502-bib-0057] R Core Team . 2022. R: A Language and Environment for Statistical Computing. R Foundation for Statistical Computing. https://www.R‐project.org/.

[gcb70502-bib-0058] Rinkes, Z. L. , I. Bertrand , B. A. Z. Amin , A. S. Grandy , K. Wickings , and M. N. Weintraub . 2016. “Nitrogen Alters Microbial Enzyme Dynamics but Not Lignin Chemistry During Maize Decomposition.” Biogeochemistry 128, no. 1: 171–186. 10.1007/s10533-016-0201-0.

[gcb70502-bib-0059] Robinson, M. D. , D. J. McCarthy , and G. K. Smyth . 2010. “edgeR: A Bioconductor Package for Differential Expression Analysis of Digital Gene Expression Data.” Bioinformatics 26, no. 1: 139–140. 10.1093/bioinformatics/btp616. 19910308 PMC2796818

[gcb70502-bib-0060] Roy Chowdhury, T. , J.‐Y. Lee , E. M. Bottos , et al. 2019. “Metaphenomic Responses of a Native Prairie Soil Microbiome to Moisture Perturbations.” MSystems 4, no. 4: e00061‐19. 10.1128/msystems.00061-19. 31186334 PMC6561317

[gcb70502-bib-0061] Santos, H. , and M. S. Da Costa . 2002. “Compatible Solutes of Organisms That Live in Hot Saline Environments.” Environmental Microbiology 4, no. 9: 501–509. 10.1046/J.1462-2920.2002.00335.X. 12220406

[gcb70502-bib-0062] Schaeffer, S. M. , P. M. Homyak , C. M. Boot , D. Roux‐Michollet , and J. P. Schimel . 2017. “Soil Carbon and Nitrogen Dynamics Throughout the Summer Drought in a California Annual Grassland.” Soil Biology and Biochemistry 115: 54–62. 10.1016/J.SOILBIO.2017.08.009.

[gcb70502-bib-0063] Schimel, J. P. 2018. “Life in Dry Soils: Effects of Drought on Soil Microbial Communities and Processes.” Annual Review of Ecology, Evolution, and Systematics 12: 409–432. 10.1146/annurev-ecolsys-110617.

[gcb70502-bib-0064] Schimel, J. P. , T. C. Balser , and M. Wallenstein . 2007. “Microbial Stress‐Response Physiology and Its Implications for Ecosystem Function.” Ecology 88, no. 6: 1386–1394. 10.1890/06-0219. 17601131

[gcb70502-bib-0065] Schostag, M. D. , C. N. Albers , C. S. Jacobsen , and A. Priemé . 2020. “Low Turnover of Soil Bacterial rRNA at Low Temperatures.” Frontiers in Microbiology 11: 962. 10.3389/fmicb.2020.00962. 32523564 PMC7261852

[gcb70502-bib-0066] Schulze‐Makuch, D. , D. Wagner , S. P. Kounaves , et al. 2018. “Transitory Microbial Habitat in the Hyperarid Atacama Desert.” Proceedings of the National Academy of Sciences of the United States of America 115, no. 11: 2670–2675. 10.1073/PNAS.1714341115/SUPPL_FILE/PNAS.1714341115.SAPP.PDF. 29483268 PMC5856521

[gcb70502-bib-0067] Sinsabaugh, R. L. , M. E. Gallo , C. Lauber , M. P. Waldrop , and D. R. Zak . 2005. “Extracellular Enzyme Activities and Soil Organic Matter Dynamics for Northern Hardwood Forests Receiving Simulated Nitrogen Deposition.” Biogeochemistry 75, no. 2: 201–215. 10.1007/s10533-004-7112-1.

[gcb70502-bib-0068] Söllinger, A. , A. T. Tveit , M. Poulsen , et al. 2018. “Holistic Assessment of Rumen Microbiome Dynamics Through Quantitative Metatranscriptomics Reveals Multifunctional Redundancy During Key Steps of Anaerobic Feed Degradation.” MSystems 3, no. 4: e00038‐18. 10.1128/mSystems.00038-18. 30116788 PMC6081794

[gcb70502-bib-0069] Tu, Q. , L. Lin , L. Cheng , Y. Deng , and Z. He . 2018. “NCycDB: A Curated Integrative Database for Fast and Accurate Metagenomic Profiling of Nitrogen Cycling Genes.” Bioinformatics 35, no. 6: 1040–1048. 10.1093/bioinformatics/bty741. 30165481

[gcb70502-bib-0070] Turner, B. L. , J. P. Driessen , P. M. Haygarth , and I. D. Mckelvie . 2003. “Potential Contribution of Lysed Bacterial Cells to Phosphorus Solubilisation in Two Rewetted Australian Pasture Soils.” Soil Biology and Biochemistry 35, no. 1: 187–189. 10.1016/S0038-0717(02)00244-4.

[gcb70502-bib-0071] van Bodegom, P. 2007. “Microbial Maintenance: A Critical Review on Its Quantification.” Microbial Ecology 53, no. 4: 513–523. 10.1007/s00248-006-9049-5. 17333428 PMC1915598

[gcb70502-bib-0072] Vaser, R. , D. Pavlović , and M. Šikić . 2016. “SWORD ‐ A Highly Efficient Protein Database Search.” Bioinformatics 32, no. 17: i680–i684. 10.1093/bioinformatics/btw445. 27587689

[gcb70502-bib-0073] Veach, A. M. , and L. H. Zeglin . 2020. “Historical Drought Affects Microbial Population Dynamics and Activity During Soil Drying and Re‐Wet.” Microbial Ecology 79, no. 3: 662–674. 10.1007/s00248-019-01432-5. 31482287

[gcb70502-bib-0074] Wang, H. , G. Jurasinski , J. Täumer , et al. 2023. “Linking Transcriptional Dynamics of Peat Microbiomes to Methane Fluxes During a Summer Drought in Two Rewetted Fens.” Environmental Science & Technology 57, no. 12: 5089–5101. 10.1021/acs.est.2c07461. 36926875

[gcb70502-bib-0075] Wickham, H. 2009. ggplot2: Elegant Graphics for Data Analysis. Springer Publishing Company, Incorporated.

[gcb70502-bib-0076] Wilke, A. , T. Harrison , J. Wilkening , et al. 2012. “The M5nr: A Novel Non‐Redundant Database Containing Protein Sequences and Annotations From Multiple Sources and Associated Tools.” BMC Bioinformatics 13, no. 1: 141. 10.1186/1471-2105-13-141. 22720753 PMC3410781

[gcb70502-bib-0077] Xu, L. , Z. Dong , D. Chiniquy , et al. 2021. “Genome‐Resolved Metagenomics Reveals Role of Iron Metabolism in Drought‐Induced Rhizosphere Microbiome Dynamics.” Nature Communications 12, no. 1: 3209. 10.1038/s41467-021-23553-7. PMC8163885 34050180

[gcb70502-bib-0078] Xu, L. , D. Naylor , Z. Dong , et al. 2018. “Drought Delays Development of the Sorghum Root Microbiome and Enriches for Monoderm Bacteria.” Proceedings of the National Academy of Sciences of the United States of America 115, no. 18: E4284–E4293. 10.1073/pnas.1717308115. 29666229 PMC5939072

[gcb70502-bib-0079] Xu, X. , P. E. Thornton , and W. M. Post . 2013. “A Global Analysis of Soil Microbial Biomass Carbon, Nitrogen and Phosphorus in Terrestrial Ecosystems.” Global Ecology and Biogeography 22, no. 6: 737–749. 10.1111/geb.12029.

[gcb70502-bib-0080] Zhu, A. , J. G. Ibrahim , and M. I. Love . 2019. “Heavy‐Tailed Prior Distributions for Sequence Count Data: Removing the Noise and Preserving Large Differences.” Bioinformatics 35, no. 12: 2084–2092. 10.1093/bioinformatics/bty895. 30395178 PMC6581436

