# Supplementary material for: Arctic Soil C and N Cycling Are Linked With Microbial Adaptations During Drought
Source: Glob Chang Biol. 2025 Sep 18;31(9):e70502. doi: 10.1111/gcb.70502 (PMC12445406; doi:10.1111/gcb.70502)
Supplement: Supplementary file 1 — Figure S1: Temperature at 5 cm soil depth at the sampling site measured in situ with four different HOBO Temperature data loggers from 2012 to 2017. Figure S2: Soil water content at 5 cm soil depth at the sampling site measured in situ with HOBO Soil Moisture data logger during the dry summer of 2019. Figure S3: Observed richness for functional genes and rRNA for prokaryotes and eukaryotes. Error bars represent standard deviations. Significance was assessed by linear models followed by a Tukey HSD test. Figure S4: Functional gene structures for genes annotated with SEED, CAZy and NCyc databases normalized to total mRNA content. Principal coordinate analysis on the basis of Bray–Curtis dissimilarities. Numbers in brackets show the percentage of variation explained by each axis. Vectors represent correlations of edaphic and microbial parameters with functional gene structures. Significance was assessed by a permutational test with 9999 permutations. Significant variables are shown in blue. p‐values indicate significance of differences in functional gene structures across treatments, assessed by permutational multivariate analysis of variance with 9999 permutations. Figure S5: Number of differentially expressed phyla relative to the control. Prokaryotic and eukaryotic rRNA genes on the basis of relative abundances or abundances normalized by total mRNA contents with a significant log2‐fold change (LFC, p adj. < 0.05) are shown. Figure S6: Differentially abundant potentially active prokaryotic and eukaryotic phyla. Log2‐fold changes (LFC) relative to the control are shown for phyla of which the abundance significantly differed from the control (p adj. < 0.05) in at least one treatment. Grey cells indicate treatments where abundance was not different from the control. Abundance indicates read counts in the entire dataset, normalized with Deseq2. [file GCB-31-e70502-s001.docx]

**Supplementary figures and tables**


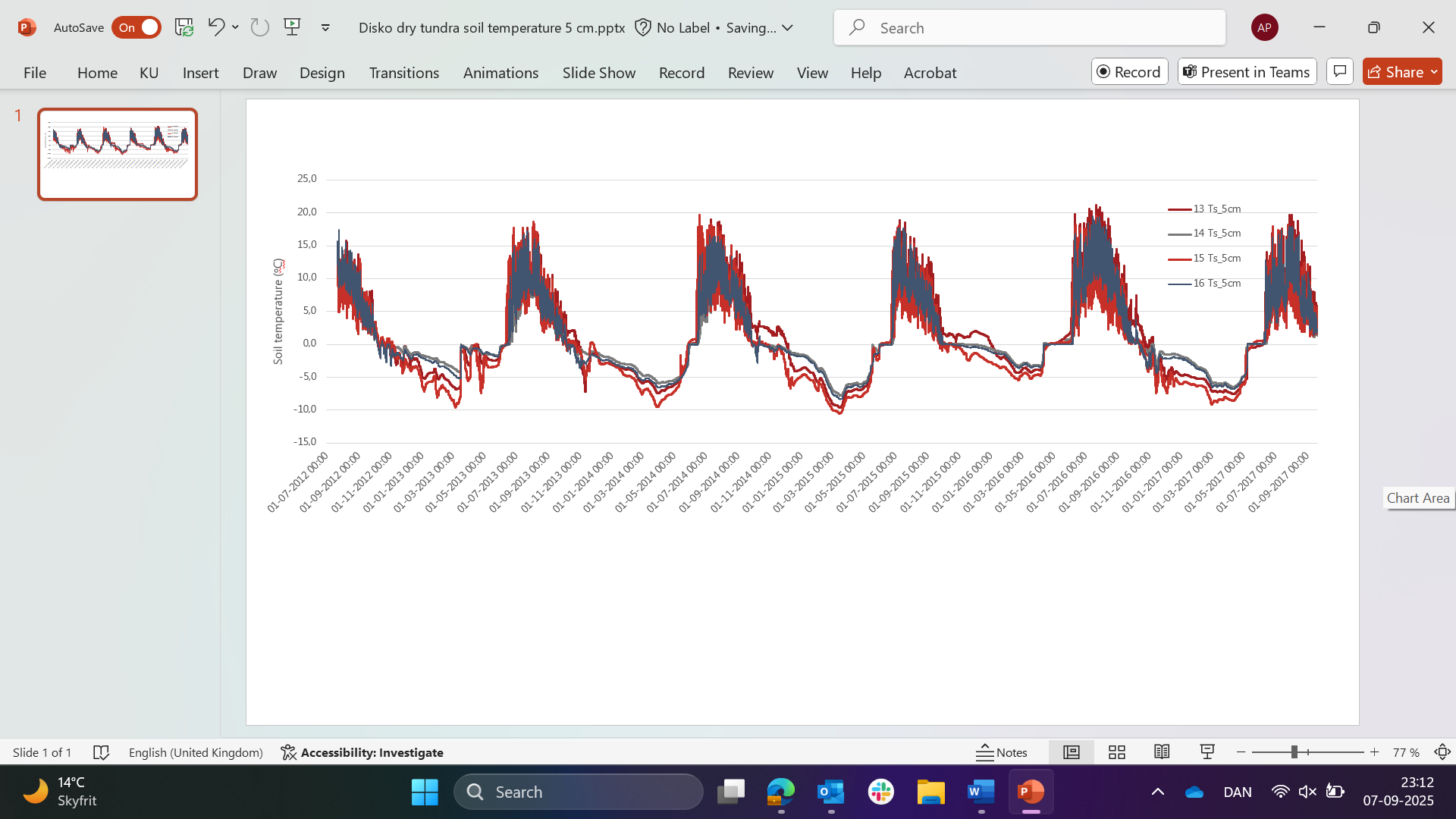


**Figure S1** Temperature at 5 cm soil depth at the sampling site measured in situ with four different HOBO Temperature data loggers from 2012 to 2017.


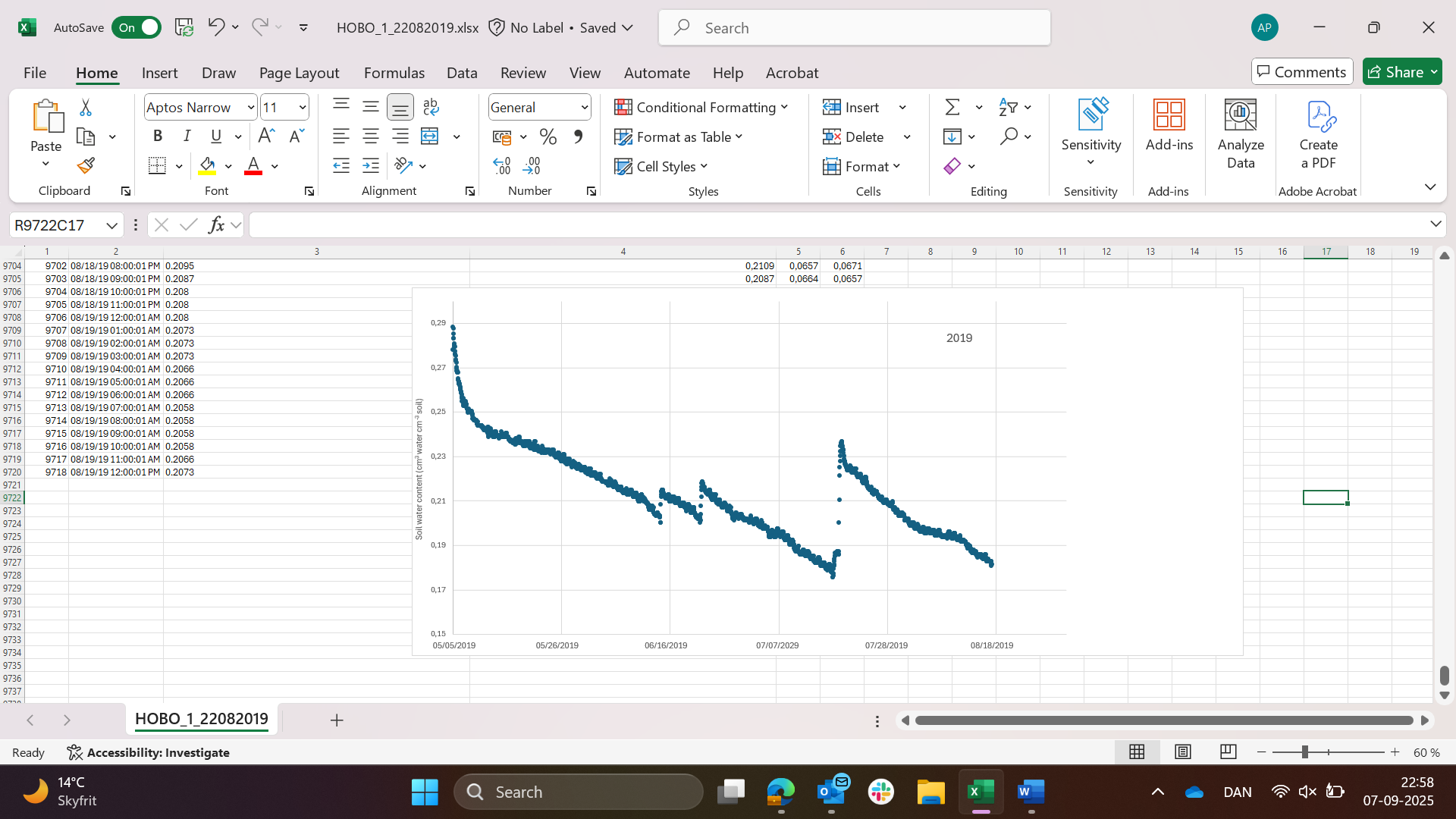


**Figure S2** Soil water content at 5 cm soil depth at the sampling site measured in situ with HOBO Soil Moisture data logger during the dry summer of 2019.


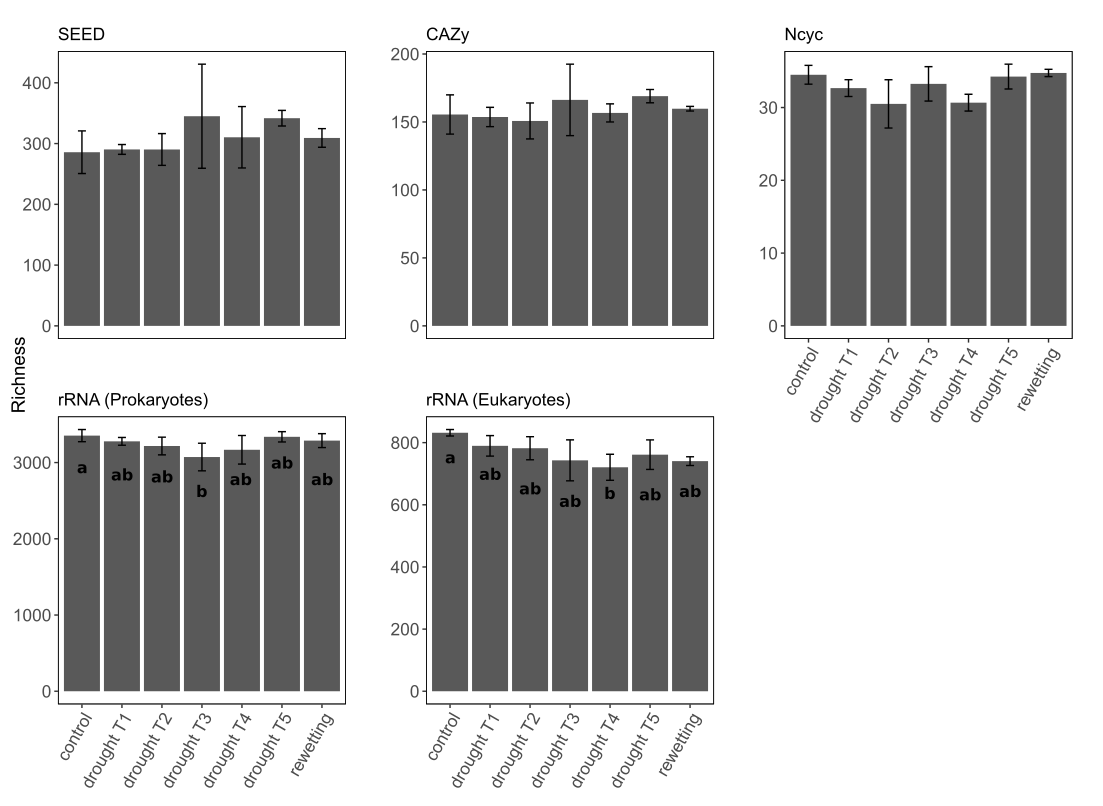


**Figure S3** Observed richness for functional genes and rRNA for Prokaryotes and Eukaryotes. Error bars represent standard deviations. Significance was assessed by linear models followed by a Tukey HSD test.


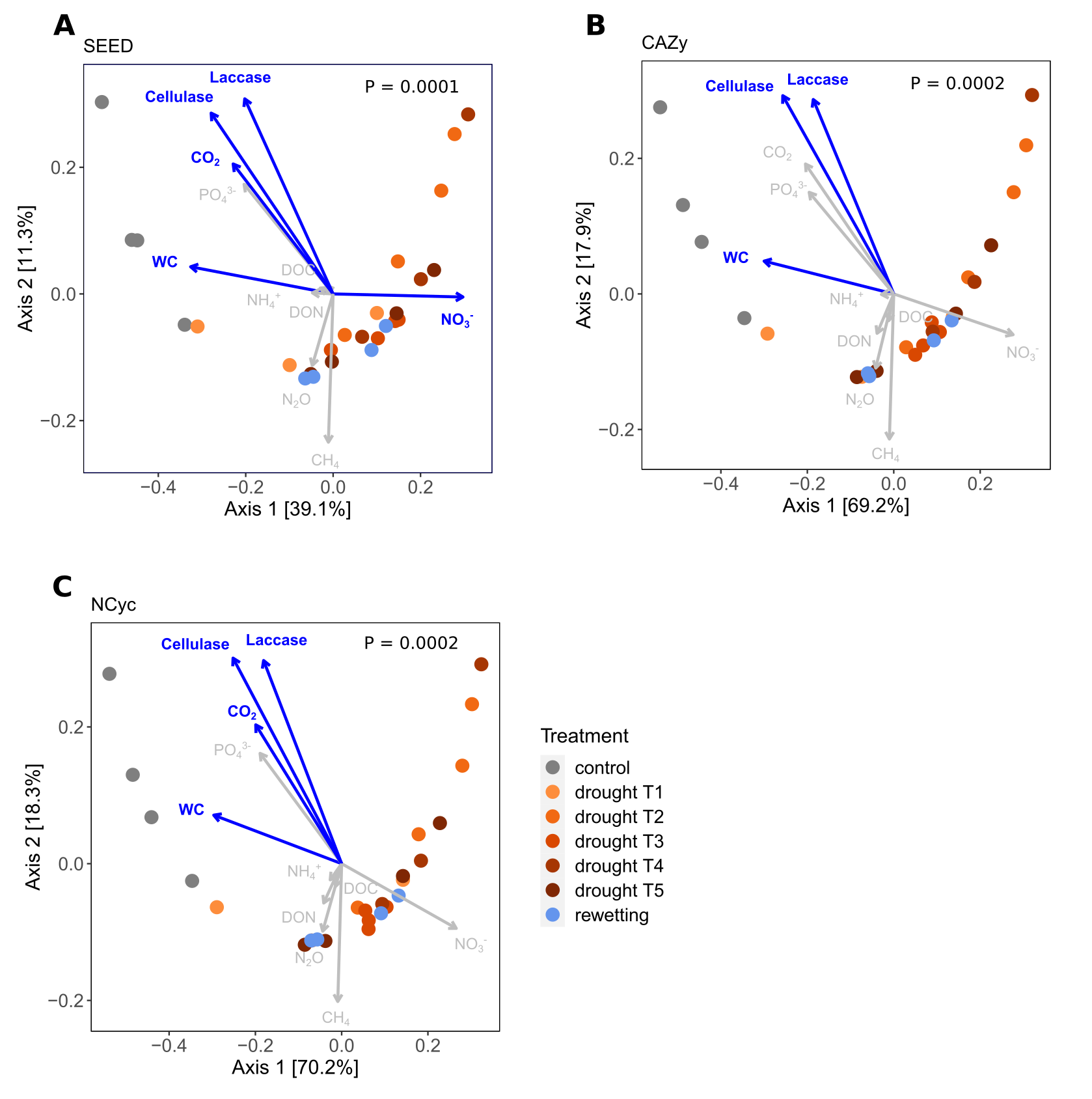


**Figure S4** Functional gene structures for genes annotated with SEED, CAZy and NCyc databases normalized to total mRNA content. Principal coordinate analysis based on Bray-Curtis dissimilarities. Numbers in brackets show the percentage of variation explained by each axis. Vectors represent correlations of edaphic and microbial parameters with functional gene structures. Significance was assessed by a permutational test with 9999 permutations. Significant variables are shown in blue. P-values indicate significance of differences in functional gene structures across treatments, assessed by permutational multivariate analysis of variance with 9999 permutations.


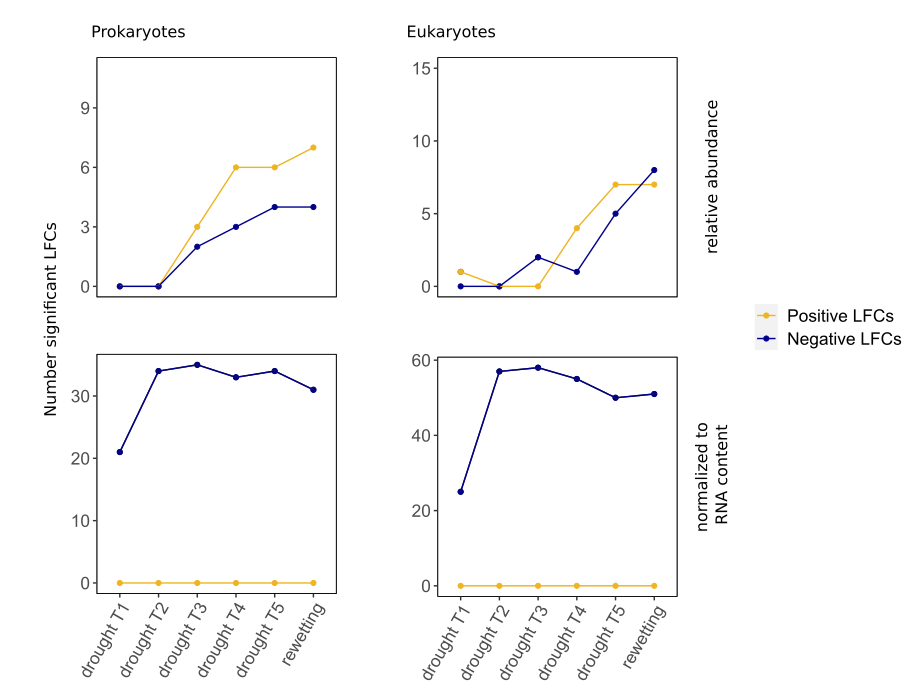


**Figure S5** Number of differentially expressed phyla relative to the control. Prokaryotic and eukaryotic rRNA genes based on relative abundances or abundances normalized by total mRNA contents with a significant log2-fold change (LFC, padj. <0.05) are shown.


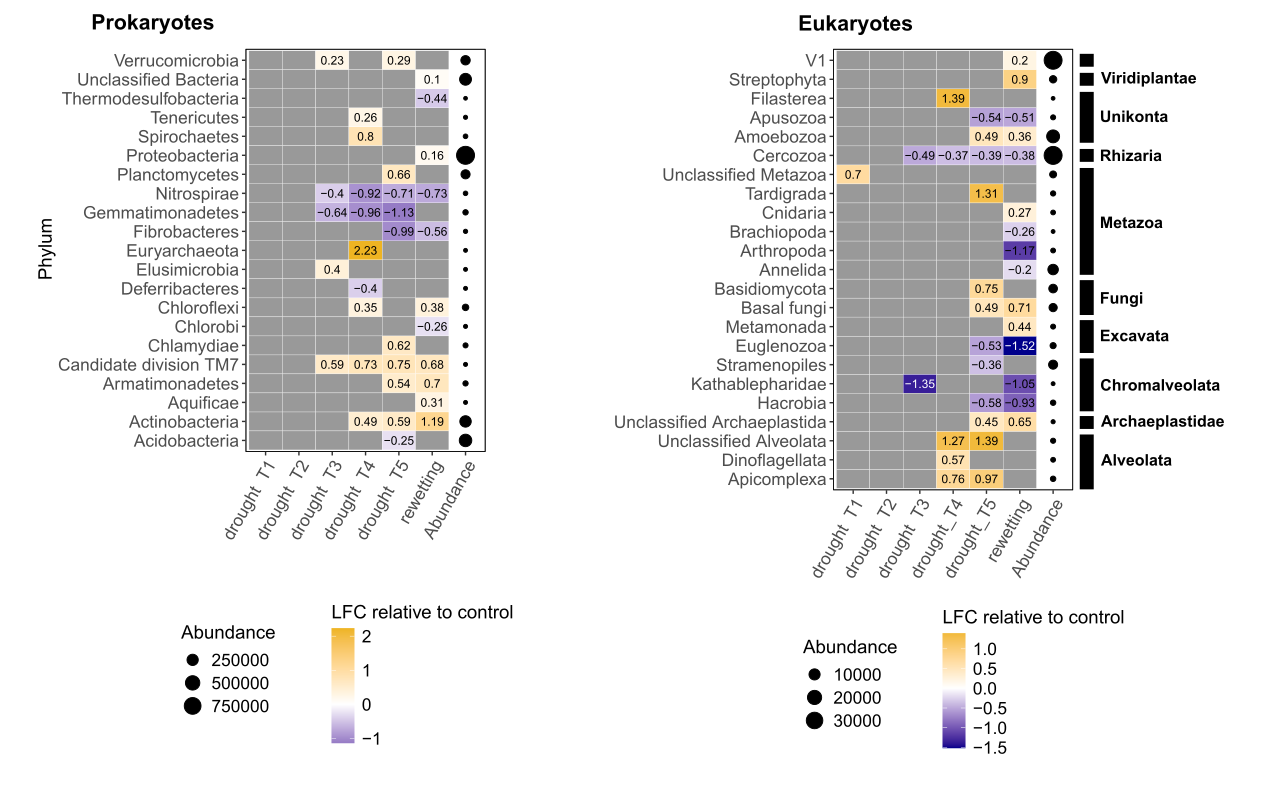


**Figure S6** Differentially abundant potentially active prokaryotic and eukaryotic phyla. Log2-fold changes (LFC) relative to the control are shown for phyla of which the abundance significantly differed from the control (P_adj._ <0.05) in at least one treatment. Grey cells indicate treatments where abundance was not different from the control. Abundance indicates read counts in the entire dataset, normalized with Deseq2.
