# Supplementary material for: Arctic Soil C and N Cycling Are Linked With Microbial Adaptations During Drought
Source: Glob Chang Biol. 2025 Sep 18;31(9):e70502. doi: 10.1111/gcb.70502 (PMC12445406; doi:10.1111/gcb.70502)
Supplement: Supplementary file 2 — Table S1: Number of reads across the steps of the bioinformatic pipeline for processing functional genes. [file GCB-31-e70502-s003.docx]

**Table S1** Number of reads across the steps of the bioinformatic pipeline for processing functional genes.

| Sample | Number reads raw data (forward) | Number reads after QC (forward) | % reads after QC | Number  reads mRNA (forward) | % reads  mrna | Number mapped reads (forward) | Number mapped reads (reverse) | Number mapped reads (mean) | % mapped reads (mean) | Number reads annotated SEED | Number reads annotated CAZy | Number reads annotated Ncyc |
| --- | --- | --- | --- | --- | --- | --- | --- | --- | --- | --- | --- | --- |
| 1 | 10 806 379 | 10 340 843 | 96% | 815 894 | 7.89% | 485 876 | 485 632 | 485 754 | 60% | 3021 | 19169 | 5426 |
| 2 | 6 376 351 | 6 177 829 | 97% | 447 873 | 7.25% | 252 090 | 251 957 | 252 024 | 56% | 1843 | 9623 | 2812 |
| 3 | 11 285 310 | 10 951 345 | 97% | 865 236 | 7.90% | 447 851 | 447 253 | 447 552 | 52% | 3963 | 18904 | 5502 |
| 4 | 18 969 751 | 18 521 379 | 98% | 1 322 109 | 7.14% | 662 214 | 661 428 | 661 821 | 50% | 7722 | 34353 | 10742 |
| 5 | 7 013 692 | 6 423 094 | 92% | 504 867 | 7.86% | 256 893 | 256 697 | 256 795 | 51% | 2124 | 13193 | 3716 |
| 6 | 3 | 3 | 100% | 1 | 33.33% | - | - | - | 0% |  |  |  |
| 7 | 9 106 503 | 8 881 939 | 98% | 560 147 | 6.31% | 297 806 | 297 322 | 297 564 | 53% | 3360 | 12793 | 4079 |
| 8 | 11 911 846 | 11 564 815 | 97% | 720 600 | 6.23% | 380 591 | 380 046 | 380 319 | 53% | 4808 | 16310 | 5085 |
| 9 | 5 440 239 | 3 342 530 | 61% | 269 550 | 8.06% | 139 455 | 139 346 | 139 401 | 52% | 1139 | 7807 | 2114 |
| 10 | 5 446 205 | 4 356 202 | 80% | 358 178 | 8.22% | 167 103 | 167 031 | 167 067 | 47% | 1580 | 7874 | 2286 |
| 11 | 9 330 903 | 9 114 614 | 98% | 652 225 | 7.16% | 411 765 | 411 067 | 411 416 | 63% | 2579 | 16705 | 4387 |
| 12 | 11 776 633 | 10 928 572 | 93% | 838 936 | 7.68% | 464 836 | 464 513 | 464 675 | 55% | 3321 | 20607 | 6040 |
| 13 | 6 758 477 | 6 516 472 | 96% | 893 930 | 13.72% | 271 866 | 271 391 | 271 629 | 30% | 3983 | 14724 | 4941 |
| 14 | 4 191 943 | 2 478 158 | 59% | 206 846 | 8.35% | 114 750 | 114 751 | 114 751 | 55% | 856 | 4217 | 1255 |
| 15 | 5 172 212 | 5 002 736 | 97% | 776 266 | 15.52% | 201 692 | 201 480 | 201 586 | 26% | 3334 | 10855 | 3731 |
| 16 | 3 749 685 | 3 460 971 | 92% | 1 944 704 | 56.19% | 129 488 | 129 703 | 129 596 | 7% | 7290 | 16403 | 6676 |
| 17 | 2 633 823 | 2 543 225 | 97% | 389 306 | 15.31% | 111 452 | 111 328 | 111 390 | 29% | 1845 | 6200 | 2116 |
| 18 | 6 687 675 | 6 470 397 | 97% | 733 020 | 11.33% | 325 670 | 325 267 | 325 469 | 44% | 2861 | 12703 | 4092 |
| 19 | 5 330 | 4 921 | 92% | 464 | 9.43% | 196 | 197 | 197 | 42% |  |  |  |
| 20 | 2 538 959 | 2 390 840 | 94% | 231 725 | 9.69% | 110 540 | 110 623 | 110 582 | 48% | 932 | 3998 | 1265 |
| 21 | 11 112 031 | 10 912 485 | 98% | 1 112 533 | 10.20% | 488 015 | 487 715 | 487 865 | 44% | 5320 | 24771 | 7374 |
| 22 | 11 570 982 | 11 246 641 | 97% | 1 039 878 | 9.25% | 457 509 | 457 124 | 457 317 | 44% | 4625 | 24206 | 7140 |
| 23 | 9 289 248 | 9 082 439 | 98% | 899 414 | 9.90% | 428 711 | 428 326 | 428 519 | 48% | 3413 | 15096 | 4153 |
| 24 | 9 584 035 | 9 297 449 | 97% | 903 754 | 9.72% | 424 812 | 424 167 | 424 490 | 47% | 3521 | 19212 | 5397 |
| 25 | 10 123 921 | 9 861 543 | 97% | 768 793 | 7.80% | 342 653 | 342 684 | 342 669 | 45% | 5832 | 17896 | 6605 |
| 26 | 9 142 499 | 8 856 129 | 97% | 681 815 | 7.70% | 318 820 | 318 695 | 318 758 | 47% | 4346 | 15054 | 5044 |
| 27 | 10 858 395 | 10 656 211 | 98% | 770 179 | 7.23% | 393 402 | 393 062 | 393 232 | 51% | 4888 | 17772 | 6142 |
| 28 | 10 031 338 | 9 794 565 | 98% | 857 743 | 8.76% | 313 748 | 313 674 | 313 711 | 37% | 5778 | 16950 | 5536 |
| Total | 220 914 368 | 209 178 347 |  | 19 565 986 |  | 8 399 804 | 8 392 479 | 8 396 142 |  | 94 284 | 397 395 | 123 656 |
| Mean | 7 889 799 | 7 470 655 | 93% | 698 785 | 12% | 299 993 | 299 731 | 299 862 | 44% | 3 626 | 15 284 | 4 756 |
| Standard deviation | 4 140 987 | 4 177 785 | 10% | 404 011 | 10% | 162 228 | 162 037 | 162 133 | 14% | 1828 | 6746 | 2127 |
