# Supplementary material for: Arctic Soil C and N Cycling Are Linked With Microbial Adaptations During Drought
Source: Glob Chang Biol. 2025 Sep 18;31(9):e70502. doi: 10.1111/gcb.70502 (PMC12445406; doi:10.1111/gcb.70502)
Supplement: Supplementary file 3 — Data S1: gcb70502‐sup‐0003‐Supinfo1.html. [file GCB-31-e70502-s002.html]

Data analysis for the study Arctic soil C and N cycling are linked with microbial adaptations during drought


# Data analysis for the study Arctic soil C and N cycling are linked with microbial adaptations during drought

# Data analysis for the study Arctic soil C and N cycling are linked with microbial adaptations during drought

## Setup

```
library(phyloseq)
library(DESeq2)
library(tidyverse)
library(vegan)
library(grid)
library(reshape2)
library(gridExtra)
library(egg)
library(RColorBrewer)
library(ggnewscale)
library(apeglm)
sessionInfo()
```

```
## R version 4.3.3 (2024-02-29 ucrt)
## Platform: x86_64-w64-mingw32/x64 (64-bit)
## Running under: Windows 11 x64 (build 26100)
## 
## Matrix products: default
## 
## 
## locale:
## [1] LC_COLLATE=English_United States.utf8 
## [2] LC_CTYPE=English_United States.utf8   
## [3] LC_MONETARY=English_United States.utf8
## [4] LC_NUMERIC=C                          
## [5] LC_TIME=English_United States.utf8    
## 
## time zone: Europe/Copenhagen
## tzcode source: internal
## 
## attached base packages:
## [1] grid      stats4    stats     graphics  grDevices utils     datasets 
## [8] methods   base     
## 
## other attached packages:
##  [1] apeglm_1.24.0               ggnewscale_0.4.10          
##  [3] RColorBrewer_1.1-3          egg_0.4.5                  
##  [5] gridExtra_2.3               reshape2_1.4.4             
##  [7] vegan_2.6-4                 lattice_0.22-5             
##  [9] permute_0.9-7               lubridate_1.9.3            
## [11] forcats_1.0.0               stringr_1.5.1              
## [13] dplyr_1.1.4                 purrr_1.0.2                
## [15] readr_2.1.5                 tidyr_1.3.1                
## [17] tibble_3.2.1                ggplot2_3.5.0              
## [19] tidyverse_2.0.0             DESeq2_1.42.1              
## [21] SummarizedExperiment_1.32.0 Biobase_2.62.0             
## [23] MatrixGenerics_1.14.0       matrixStats_1.2.0          
## [25] GenomicRanges_1.54.1        GenomeInfoDb_1.38.8        
## [27] IRanges_2.36.0              S4Vectors_0.40.2           
## [29] BiocGenerics_0.48.1         phyloseq_1.46.0            
## 
## loaded via a namespace (and not attached):
##  [1] bitops_1.0-7            rlang_1.1.3             magrittr_2.0.3         
##  [4] ade4_1.7-23             compiler_4.3.3          mgcv_1.9-1             
##  [7] vctrs_0.6.5             pkgconfig_2.0.3         crayon_1.5.3           
## [10] fastmap_1.2.0           XVector_0.42.0          rmarkdown_2.29         
## [13] tzdb_0.4.0              xfun_0.52               zlibbioc_1.48.2        
## [16] cachem_1.1.0            jsonlite_1.8.8          biomformat_1.30.0      
## [19] rhdf5filters_1.14.1     DelayedArray_0.28.0     Rhdf5lib_1.24.2        
## [22] BiocParallel_1.36.0     parallel_4.3.3          cluster_2.1.8.1        
## [25] R6_2.6.1                bslib_0.9.0             stringi_1.8.3          
## [28] jquerylib_0.1.4         numDeriv_2016.8-1.1     Rcpp_1.0.12            
## [31] iterators_1.0.14        knitr_1.50              Matrix_1.6-5           
## [34] splines_4.3.3           igraph_2.1.4            timechange_0.3.0       
## [37] tidyselect_1.2.1        rstudioapi_0.17.1       abind_1.4-8            
## [40] yaml_2.3.8              codetools_0.2-20        plyr_1.8.9             
## [43] withr_3.0.2             coda_0.19-4.1           evaluate_1.0.3         
## [46] survival_3.5-8          Biostrings_2.70.3       pillar_1.10.2          
## [49] foreach_1.5.2           generics_0.1.4          RCurl_1.98-1.14        
## [52] emdbook_1.3.13          hms_1.1.3               scales_1.4.0           
## [55] glue_1.7.0              tools_4.3.3             data.table_1.15.4      
## [58] locfit_1.5-9.10         mvtnorm_1.2-4           rhdf5_2.46.1           
## [61] ape_5.8-1               bbmle_1.0.25.1          bdsmatrix_1.3-7        
## [64] colorspace_2.1-0        nlme_3.1-164            GenomeInfoDbData_1.2.11
## [67] cli_3.6.2               S4Arrays_1.2.1          gtable_0.3.6           
## [70] sass_0.4.10             digest_0.6.35           SparseArray_1.2.4      
## [73] farver_2.1.1            htmltools_0.5.8.1       multtest_2.58.0        
## [76] lifecycle_1.0.4         MASS_7.3-60.0.1
```

```
# Settings for graphics 
# plot settings
plot.theme1 <- theme(panel.grid.major = element_blank(),
                     panel.grid.minor = element_blank(),
                     panel.background = element_rect(fill = "white",
                                                     colour = "black",
                                                     size = 0.5, linetype = "solid"),
                     panel.border= element_rect(fill=NA,size = 0.5, linetype = 'solid',colour = "black"),
                     axis.text.x = element_text(size=13),axis.text.y = element_text(size=13),legend.text = element_text(size=13),
                     axis.title = element_text(size=14),
                     legend.title = element_text(color = "black", size = 14),
                     strip.text.x = element_text(size=14),
                     strip.background = element_rect(colour="black", fill="white")
)

# function to create colors
ColCreate <- colorRampPalette(brewer.pal(9, "Set1")) # function interpolating palette to desired number of colors
```

## function for aggregating gene counts

```
#countab: counttable only numeric part,taxa are rows
#taxo=taxonomy table
#col2matchcount vector of rownames or column in counttab to which order of taxonomy table should be matched
#col2matchtax:vector of rownames or column in taxo to match
#Taxlevel: taxonomic level on which should be aggregated
#Samp: sample data
#fac: factor in Sample of which the mean should be made
#Summarize: should rare taxa be summarized and represented as others
#sumlevel: abundance threshold below which taxa are summarkued as others

AbuTableInteger=function(countab,taxo,col2matchcount,col2matchtax,Taxlevel,Samp,fac,Summarize=F,sumlevel=NULL){
  #replace NA with unclassified
  taxo=as.data.frame(apply(taxo,2,function(x){
    sapply(x,function(y){ifelse(is.na(y),"unclassified",y)})
  }))
  
  
  tax=taxo[match(col2matchcount,col2matchtax),]
  
  
  
  ## Abundance at chosen level
  taxabu=aggregate(countab,list(tax[,Taxlevel]),sum)
  rownames(taxabu)=taxabu[,1]
  taxabu=taxabu[,-1]
  
  taxabu <- as.data.frame(t(taxabu))
  
  for(i in 1:ncol(Samp)){
    Samp[,i]=as.character(Samp[,i])
  }
  
  # reorder rows of aggregated count table according to sample if not in same order
  if (!(all(rownames(taxabu)==rownames(Samp)))){
    taxabu <- taxab[rownames(Samp),]
  }
  
  taxabu.mean=aggregate(taxabu,list(Samp[,fac]),mean)
  colnames(taxabu.mean)[1]=fac
  rownames(taxabu.mean)=taxabu.mean[,1]
  
  a=c()
  for (i in colnames(taxabu.mean)){
    a[i]=is.numeric(taxabu.mean[,i])
  }
  
  taxabu.mean=taxabu.mean[,a]
  
  
  if(Summarize==T){
    num=taxabu.mean
    num.l=as.list(as.data.frame(t(num)))
    
    num.l=lapply(num.l,function(x){
      names(x)=colnames(num)
      Others=sum(x[which(x<sumlevel)])
      x=x[-which(x<sumlevel)]
      names(Others)="Others"
      x=c(x,Others)
    })
    taxabu.mean <-as.data.frame(do.call(rbind, lapply(num.l, "[", unique(unlist(sapply(num.l,names))))))
    
    colnames(taxabu.mean)=unique(unlist(sapply(num.l,names)))
    
    taxabu.mean=apply(taxabu.rel.mean,2,function(x){
      sapply(x,function(y){ifelse(is.na(y),0,y)})
    })
    
  }
  
  
  a=as.data.frame(Samp[!duplicated(Samp[,fac]),])
  
  if(all(rownames(taxabu.mean)==a[,fac])){
    taxabu.mean=cbind(taxabu.mean,a)
  }else{
    taxabu.mean=taxabu.mean[match(a[,fac],rownames(taxabu.mean)),]
    taxabu.mean=cbind(taxabu.mean,a)
  }
  
  
  return(taxabu.mean)
}
```

## Import sample file

```
# Samples sheet with design and metadata (microbial activity + soil chemistry)
sample <- read.csv("../input/sample.csv", sep = ",") 
rownames(sample) <- sample[,1]
# remove samples with too few reads (sample 6: no reads mapped; sample 19: about 300 reads mapped)
sample <- sample[-c(6,19),]

# RNA contents per g dry weight

# dry weight (wet weight /(1+water content)), exactly 1g was used for extraction
sample$DW <- 1/(1+(sample$WC/100))

# RNA per g dry weight
sample$RNA_DW <- (sample$RNA * 100) / sample$DW

# center by the mean
sample$RNA_DW_sc <- sample$RNA_DW/mean(sample$RNA_DW)

#add proportion of mrna reads over total reads

## import tables with number of reads for mrna and rrna for each sample
#mrna
mrna <- as.data.frame(read.table("../input/mrnaReadCounts.txt", sep = "",row.names = 1,header = T) )
sample$mrna <- mrna[rownames(sample),]
rm(mrna)

#rrna
rrna <- as.data.frame(read.table("../input/rrnaReadCounts.txt", sep = "",row.names = 1,header = T) )
#adapt rownames to match those in sample and mrna
rownames(rrna) <- gsub("rRNA","nonrRNA",rownames(rrna))
sample$rrna <- rrna[rownames(sample),]
rm(rrna)

#calculate fraction mrna from total rna
sample$mrna_rat <- sample$mrna/(sample$mrna+sample$rrna)

#weight RNA content by mrna fraction
sample$RNA_DW_mrna <- sample$RNA_DW*sample$mrna_rat

# scale by the mean
sample$RNA_DW_mrna_sc <- sample$RNA_DW_mrna/mean(sample$RNA_DW_mrna)
```

## Differential gene expression

### SEED

```
#### Import count table
count <- read.csv("../input/contigAbundanceAll.tsv", sep = "\t",row.names = 1) 
colnames(count) <- gsub("^X","",colnames(count))
# remove samples with too few reads (sample 6: no reads mapped; sample 19: about 300 reads mapped) and reorder count data according to sample sheet
count <- count[,rownames(sample)]

#### Import annotation tables
# seed
seed <- read.csv("../input/SEED.csv", sep = ",",row.names = 1)
#replace NA with "unclassified"
for (i in 2:6){
  seed[,i][is.na(seed[,i])] <- "unclassified"
}

# remove unclassified from annotation and count table
seed <- seed[seed$lvl4!='unclassified',]

# count table with contigs annotated in seed
count <- count[rownames(count)%in%seed$contig,]

seedLvl4Unique <- seed
# get combinations of contig and level 4
seedLvl4Unique[,"contigLvl4"] <- paste(seedLvl4Unique$contig,seedLvl4Unique$lvl4,sep = ";")
seedLvl4Unique <- seedLvl4Unique[!duplicated(seedLvl4Unique$contigLvl4),]

# duplicate contigs in count table if there are multiple annotations such that rownames of count table match annotation table
#remove suffix .1 for multiple annotations to make name rownames equal to those in count table for first occurrence
rownames(seedLvl4Unique) <- gsub("\\.1$","",rownames(seedLvl4Unique))

for (i in rownames(seedLvl4Unique)[!(rownames(seedLvl4Unique)%in%rownames(count))]){
  count[i,1:26] <- count[rownames(count)==seedLvl4Unique[i,"contig"],1:26]
}


# add column with combinations of all levels 
seed$lvl1.lvl2.lvl3 <- paste(seed$lvl1, seed$lvl2, seed$lvl3, sep = ";;")

all(colnames(count)==sample$sampleID)
```

```
## [1] TRUE
```

```
all(rownames(count)%in%rownames(seedLvl4Unique))
```

```
## [1] TRUE
```

```
all(rownames(seedLvl4Unique)%in%rownames(count))
```

```
## [1] TRUE
```

```
# aggregate at lvl4 (gene)
countLvl4 <- AbuTableInteger(countab = count, taxo = seedLvl4Unique, col2matchcount = rownames(count),
                             col2matchtax = rownames(seedLvl4Unique), Taxlevel = "lvl4", Samp = sample, fac = "sampleID")

#remove sample data
countLvl4 <- countLvl4[,-(1358:1379)]
countLvl4 <- as.data.frame(t(countLvl4))


## use only a subset of genes in level 1 categories of interest to reduce multiple testing burden 
# categories 
cat <- c("Respiration", "Protein Metabolism", "RNA Metabolism", "DNA Metabolism", "Stress Response", "Membrane Transport", "Amino Acids and Derivatives", "Motility and Chemotaxis",                   
"Regulation and Cell signaling","Nucleosides and Nucleotides", "Cell Wall and Capsule", "Phosphorus Metabolism", "Cell Division and Cell Cycle", "Dormancy and Sporulation")                          
#subset aggregated count table
countLvl4 <- countLvl4[rownames(countLvl4)%in%seedLvl4Unique$lvl4[seedLvl4Unique$lvl1%in%cat],]


colSums(countLvl4)
```

```
##   1_S1_nonrRNA   2_S2_nonrRNA   3_S3_nonrRNA   4_S4_nonrRNA   5_S5_nonrRNA 
##           1866           1121           2453           5013           1412 
##   7_S7_nonrRNA   8_S8_nonrRNA   9_S9_nonrRNA 10_S10_nonrRNA 11_S11_nonrRNA 
##           2166           2978            642            939           1580 
## 12_S12_nonrRNA 13_S13_nonrRNA 14_S14_nonrRNA 15_S15_nonrRNA 16_S16_nonrRNA 
##           1898           2325            532           1973           4158 
## 17_S17_nonrRNA 18_S18_nonrRNA 20_S20_nonrRNA 21_S21_nonrRNA 22_S22_nonrRNA 
##            996           1619            554           3061           2655 
## 23_S23_nonrRNA 24_S24_nonrRNA 25_S25_nonrRNA 26_S26_nonrRNA 27_S27_nonrRNA 
##           1959           1995           3688           2635           3052 
## 28_S28_nonrRNA 
##           3623
```

```
# remove unneeded categories also from annotation
seed <- seed[seed$lvl1%in%cat,]

#create Deseq object
all(colnames(countLvl4)==rownames(sample))
```

```
## [1] TRUE
```

```
dds <- DESeqDataSetFromMatrix(countData = countLvl4, colData = sample, design = ~treatment)
```

```
## converting counts to integer mode
```

```
##   Note: levels of factors in the design contain characters other than
##   letters, numbers, '_' and '.'. It is recommended (but not required) to use
##   only letters, numbers, and delimiters '_' or '.', as these are safe characters
##   for column names in R. [This is a message, not a warning or an error]
```

```
dds <- DESeq(dds, test="Wald", fitType="local",sfType = "poscounts")
```

```
## estimating size factors
##   Note: levels of factors in the design contain characters other than
##   letters, numbers, '_' and '.'. It is recommended (but not required) to use
##   only letters, numbers, and delimiters '_' or '.', as these are safe characters
##   for column names in R. [This is a message, not a warning or an error]
```

```
## estimating dispersions
```

```
## gene-wise dispersion estimates
```

```
## mean-dispersion relationship
```

```
##   Note: levels of factors in the design contain characters other than
##   letters, numbers, '_' and '.'. It is recommended (but not required) to use
##   only letters, numbers, and delimiters '_' or '.', as these are safe characters
##   for column names in R. [This is a message, not a warning or an error]
```

```
## final dispersion estimates
```

```
## fitting model and testing
```

```
### Extract all comparisons with the control using shrinkage with apeglm (for padj <0.05 )

df <- data.frame() # dataframe to store total number of sign. lfcs, neg. and pos. sign. lfcs

### drought T1 vs control
res <- results(dds, alpha = 0.05, name = "treatment_drought.T1_vs_control")
ddsRes1 = lfcShrink(dds, coef = "treatment_drought.T1_vs_control", type="apeglm", res = res)
```

```
## using 'apeglm' for LFC shrinkage. If used in published research, please cite:
##     Zhu, A., Ibrahim, J.G., Love, M.I. (2018) Heavy-tailed prior distributions for
##     sequence count data: removing the noise and preserving large differences.
##     Bioinformatics. https://doi.org/10.1093/bioinformatics/bty895
```

```
#subset to padj < 0.05
ddsRes1_0.05 = as.data.frame(ddsRes1[which(ddsRes1$padj<0.05),])
#no sign. lfc
df['drought.T1_vs_control','total']<- nrow(ddsRes1_0.05)
df['drought.T1_vs_control','positive']<- nrow(ddsRes1_0.05[ddsRes1_0.05$log2FoldChange>0,])
df['drought.T1_vs_control','negative']<- nrow(ddsRes1_0.05[ddsRes1_0.05$log2FoldChange<0,])

### drought T2 vs control
res <- results(dds, alpha = 0.05, name = "treatment_drought.T2_vs_control")
ddsRes2 = lfcShrink(dds, coef = "treatment_drought.T2_vs_control", type="apeglm", res = res)
```

```
## using 'apeglm' for LFC shrinkage. If used in published research, please cite:
##     Zhu, A., Ibrahim, J.G., Love, M.I. (2018) Heavy-tailed prior distributions for
##     sequence count data: removing the noise and preserving large differences.
##     Bioinformatics. https://doi.org/10.1093/bioinformatics/bty895
```

```
#subset to padj < 0.05
ddsRes2_0.05 = as.data.frame(ddsRes2[which(ddsRes2$padj<0.05),])

df['drought.T2_vs_control','total']<- nrow(ddsRes2_0.05)
df['drought.T2_vs_control','positive']<- nrow(ddsRes2_0.05[ddsRes2_0.05$log2FoldChange>0,])
df['drought.T2_vs_control','negative']<- nrow(ddsRes2_0.05[ddsRes2_0.05$log2FoldChange<0,])


#add annotation
a <- list()
for(i in rownames(ddsRes2_0.05)){
  a[[i]] <- data.frame()
  for (j in 1:length(unique(paste(seed[seed$lvl4==i,"lvl1.lvl2.lvl3"],i)))){
    a[[i]][j,colnames(ddsRes2_0.05)] <- ddsRes2_0.05[i,colnames(ddsRes2_0.05)]
    a[[i]][j,"annotation"] <- unique(paste(seed[seed$lvl4==i,"lvl1.lvl2.lvl3"],i,sep=";;"))[j]
  }
}
#combine
ddsRes2_0.05 <- do.call(rbind,a)
# separate annotation
ddsRes2_0.05 <- separate(data=ddsRes2_0.05,col=annotation, into = c("lvl1","lvl2","lvl3","lvl4"),sep = ";;",remove = F)
rownames(ddsRes2_0.05) <- make.unique(ddsRes2_0.05[,"lvl4"], sep=".")


### drought T3 vs control
res <- results(dds, alpha = 0.05, name = "treatment_drought.T3_vs_control")
ddsRes3 = lfcShrink(dds, coef = "treatment_drought.T3_vs_control", type="apeglm", res = res)
```

```
## using 'apeglm' for LFC shrinkage. If used in published research, please cite:
##     Zhu, A., Ibrahim, J.G., Love, M.I. (2018) Heavy-tailed prior distributions for
##     sequence count data: removing the noise and preserving large differences.
##     Bioinformatics. https://doi.org/10.1093/bioinformatics/bty895
```

```
#subset to padj < 0.05
ddsRes3_0.05 = as.data.frame(ddsRes3[which(ddsRes3$padj<0.05),])

df['drought.T3_vs_control','total']<- nrow(ddsRes3_0.05)
df['drought.T3_vs_control','positive']<- nrow(ddsRes3_0.05[ddsRes3_0.05$log2FoldChange>0,])
df['drought.T3_vs_control','negative']<- nrow(ddsRes3_0.05[ddsRes3_0.05$log2FoldChange<0,])

#add annotation
a <- list()
for(i in rownames(ddsRes3_0.05)){
  a[[i]] <- data.frame()
  for (j in 1:length(unique(paste(seed[seed$lvl4==i,"lvl1.lvl2.lvl3"],i)))){
    a[[i]][j,colnames(ddsRes3_0.05)] <- ddsRes3_0.05[i,colnames(ddsRes3_0.05)]
    a[[i]][j,"annotation"] <- unique(paste(seed[seed$lvl4==i,"lvl1.lvl2.lvl3"],i,sep=";;"))[j]
  }
}
#combine
ddsRes3_0.05 <- do.call(rbind,a)
# separate annotation
ddsRes3_0.05 <- separate(data=ddsRes3_0.05,col=annotation, into = c("lvl1","lvl2","lvl3","lvl4"),sep = ";;",remove = F)
rownames(ddsRes3_0.05) <- make.unique(ddsRes3_0.05[,"lvl4"], sep=".")


### drought T4 vs control
res <- results(dds, alpha = 0.05, name = "treatment_drought.T4_vs_control")
ddsRes4 = lfcShrink(dds, coef = "treatment_drought.T4_vs_control", type="apeglm", res = res)
```

```
## using 'apeglm' for LFC shrinkage. If used in published research, please cite:
##     Zhu, A., Ibrahim, J.G., Love, M.I. (2018) Heavy-tailed prior distributions for
##     sequence count data: removing the noise and preserving large differences.
##     Bioinformatics. https://doi.org/10.1093/bioinformatics/bty895
```

```
#subset to padj < 0.05
ddsRes4_0.05 = as.data.frame(ddsRes4[which(ddsRes4$padj<0.05),])

df['drought.T4_vs_control','total']<- nrow(ddsRes4_0.05)
df['drought.T4_vs_control','positive']<- nrow(ddsRes4_0.05[ddsRes4_0.05$log2FoldChange>0,])
df['drought.T4_vs_control','negative']<- nrow(ddsRes4_0.05[ddsRes4_0.05$log2FoldChange<0,])


#add annotation
a <- list()
for(i in rownames(ddsRes4_0.05)){
  a[[i]] <- data.frame()
  for (j in 1:length(unique(paste(seed[seed$lvl4==i,"lvl1.lvl2.lvl3"],i)))){
    a[[i]][j,colnames(ddsRes4_0.05)] <- ddsRes4_0.05[i,colnames(ddsRes4_0.05)]
    a[[i]][j,"annotation"] <- unique(paste(seed[seed$lvl4==i,"lvl1.lvl2.lvl3"],i,sep=";;"))[j]
  }
}
#combine
ddsRes4_0.05 <- do.call(rbind,a)
# separate annotation
ddsRes4_0.05 <- separate(data=ddsRes4_0.05,col=annotation, into = c("lvl1","lvl2","lvl3","lvl4"),sep = ";;",remove = F)
rownames(ddsRes4_0.05) <- make.unique(ddsRes4_0.05[,"lvl4"], sep=".")


### drought T5 vs control
res <- results(dds, alpha = 0.05, name = "treatment_drought.T5_vs_control")
ddsRes5 = lfcShrink(dds, coef = "treatment_drought.T5_vs_control", type="apeglm", res = res)
```

```
## using 'apeglm' for LFC shrinkage. If used in published research, please cite:
##     Zhu, A., Ibrahim, J.G., Love, M.I. (2018) Heavy-tailed prior distributions for
##     sequence count data: removing the noise and preserving large differences.
##     Bioinformatics. https://doi.org/10.1093/bioinformatics/bty895
```

```
#subset to padj < 0.05
ddsRes5_0.05 = as.data.frame(ddsRes5[which(ddsRes5$padj<0.05),])

df['drought.T5_vs_control','total']<- nrow(ddsRes5_0.05)
df['drought.T5_vs_control','positive']<- nrow(ddsRes5_0.05[ddsRes5_0.05$log2FoldChange>0,])
df['drought.T5_vs_control','negative']<- nrow(ddsRes5_0.05[ddsRes5_0.05$log2FoldChange<0,])


#add annotation
a <- list()
for(i in rownames(ddsRes5_0.05)){
  a[[i]] <- data.frame()
  for (j in 1:length(unique(paste(seed[seed$lvl4==i,"lvl1.lvl2.lvl3"],i)))){
    a[[i]][j,colnames(ddsRes5_0.05)] <- ddsRes5_0.05[i,colnames(ddsRes5_0.05)]
    a[[i]][j,"annotation"] <- unique(paste(seed[seed$lvl4==i,"lvl1.lvl2.lvl3"],i,sep=";;"))[j]
  }
}
#combine
ddsRes5_0.05 <- do.call(rbind,a)
# separate annotation
ddsRes5_0.05 <- separate(data=ddsRes5_0.05,col=annotation, into = c("lvl1","lvl2","lvl3","lvl4"),sep = ";;",remove = F)
rownames(ddsRes5_0.05) <- make.unique(ddsRes5_0.05[,"lvl4"], sep=".")


### rewetting vs control
res <- results(dds, alpha = 0.05, name = "treatment_rewetting_vs_control")
ddsRes6 = lfcShrink(dds, coef = "treatment_rewetting_vs_control", type="apeglm", res = res)
```

```
## using 'apeglm' for LFC shrinkage. If used in published research, please cite:
##     Zhu, A., Ibrahim, J.G., Love, M.I. (2018) Heavy-tailed prior distributions for
##     sequence count data: removing the noise and preserving large differences.
##     Bioinformatics. https://doi.org/10.1093/bioinformatics/bty895
```

```
#subset to padj < 0.05
ddsRes6_0.05 = as.data.frame(ddsRes6[which(ddsRes6$padj<0.05),])

df['rewetting_vs_control','total']<- nrow(ddsRes6_0.05)
df['rewetting_vs_control','positive']<- nrow(ddsRes6_0.05[ddsRes6_0.05$log2FoldChange>0,])
df['rewetting_vs_control','negative']<- nrow(ddsRes6_0.05[ddsRes6_0.05$log2FoldChange<0,])


#add annotation
a <- list()
for(i in rownames(ddsRes6_0.05)){
  a[[i]] <- data.frame()
  for (j in 1:length(unique(paste(seed[seed$lvl4==i,"lvl1.lvl2.lvl3"],i)))){
    a[[i]][j,colnames(ddsRes6_0.05)] <- ddsRes6_0.05[i,colnames(ddsRes6_0.05)]
    a[[i]][j,"annotation"] <- unique(paste(seed[seed$lvl4==i,"lvl1.lvl2.lvl3"],i,sep=";;"))[j]
  }
}
#combine
ddsRes6_0.05 <- do.call(rbind,a)
# separate annotation
ddsRes6_0.05 <- separate(data=ddsRes6_0.05,col=annotation, into = c("lvl1","lvl2","lvl3","lvl4"),sep = ";;",remove = F)
rownames(ddsRes6_0.05) <- make.unique(ddsRes6_0.05[,"lvl4"], sep=".")


### make combined data frame for all treatments with lfc and annotation
genes <- unique(c(rownames(ddsRes1_0.05),rownames(ddsRes2_0.05),rownames(ddsRes3_0.05),
                  rownames(ddsRes4_0.05),rownames(ddsRes5_0.05),rownames(ddsRes6_0.05)))

#create table with annotations for genes with sign. LFC in any of the treatment; and basemeans
#include only the treatments where sign. LFCs were found
anno <- unique(rbind(ddsRes2_0.05[,c("lvl1","lvl2","lvl3","lvl4","baseMean")],
                     ddsRes3_0.05[,c("lvl1","lvl2","lvl3","lvl4","baseMean")],ddsRes4_0.05[,c("lvl1","lvl2","lvl3","lvl4","baseMean")],
                     ddsRes5_0.05[,c("lvl1","lvl2","lvl3","lvl4","baseMean")],ddsRes6_0.05[,c("lvl1","lvl2","lvl3","lvl4","baseMean")]))
anno[,"lvl4unique"] <- rownames(anno)

ddsResAll_0.05 <- data.frame()
for (i in genes){
  ddsResAll_0.05[i,"drought_T1"] <- ifelse(i%in%rownames(ddsRes1_0.05),ddsRes1_0.05[i,"log2FoldChange"],NA)
  ddsResAll_0.05[i,"drought_T2"] <- ifelse(i%in%rownames(ddsRes2_0.05),ddsRes2_0.05[i,"log2FoldChange"],NA)
  ddsResAll_0.05[i,"drought_T3"] <- ifelse(i%in%rownames(ddsRes3_0.05),ddsRes3_0.05[i,"log2FoldChange"],NA)
  ddsResAll_0.05[i,"drought_T4"] <- ifelse(i%in%rownames(ddsRes4_0.05),ddsRes4_0.05[i,"log2FoldChange"],NA)
  ddsResAll_0.05[i,"drought_T5"] <- ifelse(i%in%rownames(ddsRes5_0.05),ddsRes5_0.05[i,"log2FoldChange"],NA)
  ddsResAll_0.05[i,"rewetting"] <- ifelse(i%in%rownames(ddsRes6_0.05),ddsRes6_0.05[i,"log2FoldChange"],NA)
  ddsResAll_0.05[i,c("lvl1","lvl2","lvl3","lvl4")] <- anno[i,c("lvl1","lvl2","lvl3","lvl4")]
}

# add a columns with the rownmaes to distiniguish genes with duplicate annotaitons
ddsResAll_0.05[,"lvl4unique"] <- rownames(ddsResAll_0.05)

#reorder according to lvl1 and 4
ddsResAll_0.05 <- ddsResAll_0.05[order(ddsResAll_0.05$lvl1,ddsResAll_0.05$lvl4),]


# add column with lvl1 lvl4 combinations and subset to unique
ddsResAll_0.05$lvl1.lvl4 <- paste(ddsResAll_0.05$lvl1,ddsResAll_0.05$lvl4, sep = ';;')
ddsResAll_0.05 <- ddsResAll_0.05[!duplicated(ddsResAll_0.05$lvl1.lvl4),]

# re-add a columns with the rownames as column <=> drop multiple annotations at lvl2 and 3
ddsResAll_0.05[,"lvl4unique"] <- rownames(ddsResAll_0.05)

# subset table with annotation and basemean accordingly
anno <- anno[rownames(anno)%in%ddsResAll_0.05$lvl4unique,]

# transform to long format
ddsResAll_0.05 <- melt(ddsResAll_0.05,id.vars = c("lvl1","lvl2","lvl3","lvl4","lvl4unique","lvl1.lvl4"))

# reorder level of genes according to category
ddsResAll_0.05$lvl4unique <- factor(ddsResAll_0.05$lvl4unique, levels = rev(unique(ddsResAll_0.05$lvl4unique[order(ddsResAll_0.05$lvl1,
                                                                                                                   ddsResAll_0.05$lvl4)])))

# separate plot for each level 1 category; first plot with legend
# scale of legend (points and gradient based on entire dataset)
for (i in unique(ddsResAll_0.05$lvl1)){
  
  if(which(unique(ddsResAll_0.05$lvl1)==i)==length(unique(ddsResAll_0.05$lvl1))){
    p <-
      ggplot(ddsResAll_0.05[ddsResAll_0.05$lvl1==i,],aes(variable,lvl4unique,fill=value))+
      geom_tile(color= "white",size=0.1) +
      geom_text(aes(label = round(value,digits=2)),size = 3)+
      scale_fill_gradient2(low = "blue4", mid = "white", high = "goldenrod2", midpoint = 0, space = "Lab",
                           na.value = "grey60", guide = "colourbar",aesthetics = "fill", name="LFC relative to control",
                           limits = c(min(ddsResAll_0.05$value,na.rm = T),max(ddsResAll_0.05$value, na.rm = T)))+
      plot.theme1+
      theme(axis.text.x = element_text(angle = 60, hjust = 1)) +
      geom_point(data = anno[anno$lvl1==i,],aes(x="Abundance",y=lvl4unique,size=baseMean),inherit.aes = F)+
      scale_size_continuous(limits = c(min(anno$baseMean),max(anno$baseMean)))+
      theme(legend.position=ifelse(which(unique(ddsResAll_0.05$lvl1)==i)==1,"top","none"))+
      
      ggtitle(label = i)
  }else{
    p <-
      ggplot(ddsResAll_0.05[ddsResAll_0.05$lvl1==i,],aes(variable,lvl4unique,fill=value))+
      geom_tile(color= "white",size=0.1) +
      geom_text(aes(label = round(value,digits=2)),size = 3)+
      scale_fill_gradient2(low = "blue4", mid = "white", high = "goldenrod2", midpoint = 0, space = "Lab",
                           na.value = "grey60", guide = "colourbar",aesthetics = "fill", name="LFC relative to control",
                           limits = c(min(ddsResAll_0.05$value,na.rm = T),max(ddsResAll_0.05$value, na.rm = T)))+
      plot.theme1+
      theme(axis.text.x = element_blank()) +
      geom_point(data = anno[anno$lvl1==i,],aes(x="Abundance",y=lvl4unique,size=baseMean),inherit.aes = F)+
      scale_size_continuous(limits = c(min(anno$baseMean),max(anno$baseMean)))+
      theme(legend.position=ifelse(which(unique(ddsResAll_0.05$lvl1)==i)==1,"top","none"))+
      
      ggtitle(label = i)
  }

  p <- set_panel_size(p,margin = unit(0, "mm"), width = unit(2.5, "inch"),
                      height = unit(length(unique(ddsResAll_0.05$lvl4unique[ddsResAll_0.05$lvl1==i]))/6, "inch"))
  assign(paste0("p",which(unique(ddsResAll_0.05$lvl1)==i)),p)
  rm(p)

}


grid.arrange(p1,p2,p3,p4,p5,p6,p7,p8,p9,p10,p11,p12,ncol=2)
```

```
# Plot with number of sign LFCs per treatment
df$treatment <- rownames(df)
df <- melt(df)
```

```
## Using treatment as id variables
```

```
df$treatment <- gsub('_.*','',df$treatment)
p <- ggplot(df,aes(x=treatment,y=value,color=variable,group=variable)) + geom_point() +
  geom_line() +
  plot.theme1 + 
  theme(axis.text.x = element_text(angle = 60, hjust = 1)) +
  scale_color_manual(values = c("grey20","goldenrod2","blue4"))
p <- set_panel_size(p,margin = unit(0, "mm"), width = unit(2.5, "inch"), height = unit(2.5, "inch"))


grid.arrange(p)
```

```
rm(list=ls(pattern = "ddsRes"))
rm(p,countLvl4,anno,genes)
rm(p1,p2,p3,p4,p5,p6,p7,p8,p9,p10,p11,p12)
```

normalized to RNA content, weighted by mrna fraction

normalization by RNA content included into DESeqs size factor

```
#create Deseq object
all(colnames(countLvl4)==rownames(sample))
```

```
## [1] TRUE
```

```
dds <- DESeqDataSetFromMatrix(countData = countLvl4, colData = sample, design = ~treatment)
```

```
## converting counts to integer mode
```

```
##   Note: levels of factors in the design contain characters other than
##   letters, numbers, '_' and '.'. It is recommended (but not required) to use
##   only letters, numbers, and delimiters '_' or '.', as these are safe characters
##   for column names in R. [This is a message, not a warning or an error]
```

```
# calculate size factors to include RNA weight
sf <- estimateSizeFactors(dds, type = "poscounts")
```

```
##   Note: levels of factors in the design contain characters other than
##   letters, numbers, '_' and '.'. It is recommended (but not required) to use
##   only letters, numbers, and delimiters '_' or '.', as these are safe characters
##   for column names in R. [This is a message, not a warning or an error]
```

```
sf <- sizeFactors(sf)

# include RNA (weighted by mrna fraction) per dry weight in size factor (divide size factor by mrna content)
all(names(sf)==rownames(sample))
```

```
## [1] TRUE
```

```
sf <- sf/sample$RNA_DW_mrna_sc

#Run Deseq
sizeFactors(dds) <- sf
```

```
##   Note: levels of factors in the design contain characters other than
##   letters, numbers, '_' and '.'. It is recommended (but not required) to use
##   only letters, numbers, and delimiters '_' or '.', as these are safe characters
##   for column names in R. [This is a message, not a warning or an error]
```

```
dds <- DESeq(dds, test="Wald", fitType="local")
```

```
## using pre-existing size factors
```

```
## estimating dispersions
```

```
## gene-wise dispersion estimates
```

```
## mean-dispersion relationship
```

```
##   Note: levels of factors in the design contain characters other than
##   letters, numbers, '_' and '.'. It is recommended (but not required) to use
##   only letters, numbers, and delimiters '_' or '.', as these are safe characters
##   for column names in R. [This is a message, not a warning or an error]
```

```
## final dispersion estimates
```

```
## fitting model and testing
```

all subsequent steps are the same as for non-normalized data

### Cazy

```
#### Import count table
count <- read.csv("../input/contigAbundanceAll.tsv", sep = "\t",row.names = 1) 
colnames(count) <- gsub("^X","",colnames(count))
# remove samples with too few reads (sample 6: no reads mapped; sample 19: about 300 reads mapped) and reorder count data according to sample sheet
count <- count[,rownames(sample)]

#### Import annotation tables
# Cazy
Cazy <- read.csv("../input/CAZY.csv", sep = ",",row.names = 1)
#replace NA with "unclassified"
for (i in 2:6){
  Cazy[,i][is.na(Cazy[,i])] <- "unclassified"
}

#subset count table and annotation table to contigs annotated with Cazy
Cazy <- Cazy[Cazy$annotation!="unclassified",]
count <- count[rownames(Cazy),]

all(colnames(count)==sample$sampleID)
```

```
## [1] TRUE
```

```
# aggregate at lvl2 (gene)
countFam <- AbuTableInteger(countab = count, taxo = Cazy, col2matchcount = rownames(count),
                             col2matchtax = rownames(Cazy), Taxlevel = "Family", Samp = sample, fac = "sampleID")

#remove sample data
countFam <- countFam[,-(314:335)]

#transpose
countFam <- as.data.frame(t(countFam))
```

- Deseq with raw counts

```
#create Deseq object
all(colnames(countFam)==rownames(sample))
```

```
## [1] TRUE
```

```
dds <- DESeqDataSetFromMatrix(countData = countFam, colData = sample, design = ~treatment) 
dds <- DESeq(dds, test="Wald", fitType="local") 

### Extract all comparisons with the control using shrinkage with apeglm
#dataframe for number of sign. LFCs
df <- data.frame()

### drought T1 vs control
res <- results(dds, alpha = 0.05, name = "treatment_drought.T1_vs_control")
ddsRes1 = lfcShrink(dds, coef = "treatment_drought.T1_vs_control", type="apeglm", res = res)

#subset to padj < 0.05
ddsRes1_0.05 = as.data.frame(ddsRes1[which(ddsRes1$padj<0.05),])
#add annotation
ddsRes1_0.05$Family <- rownames(ddsRes1_0.05)

df['drought.T1_vs_control','total']<- nrow(ddsRes1_0.05)
df['drought.T1_vs_control','positive']<- nrow(ddsRes1_0.05[ddsRes1_0.05$log2FoldChange>0,])
df['drought.T1_vs_control','negative']<- nrow(ddsRes1_0.05[ddsRes1_0.05$log2FoldChange<0,])

### drought T2 vs control
res <- results(dds, alpha = 0.05, name = "treatment_drought.T2_vs_control")
ddsRes2 = lfcShrink(dds, coef = "treatment_drought.T2_vs_control", type="apeglm", res = res)
#subset to padj < 0.05
ddsRes2_0.05 = as.data.frame(ddsRes2[which(ddsRes2$padj<0.05),])
#add annotation
ddsRes2_0.05$Family <- rownames(ddsRes2_0.05)

df['drought.T2_vs_control','total']<- nrow(ddsRes2_0.05)
df['drought.T2_vs_control','positive']<- nrow(ddsRes2_0.05[ddsRes2_0.05$log2FoldChange>0,])
df['drought.T2_vs_control','negative']<- nrow(ddsRes2_0.05[ddsRes2_0.05$log2FoldChange<0,])

### drought T3 vs control
res <- results(dds, alpha = 0.05, name = "treatment_drought.T3_vs_control")
ddsRes3 = lfcShrink(dds, coef = "treatment_drought.T3_vs_control", type="apeglm",res = res)
#subset to padj < 0.05
ddsRes3_0.05 = as.data.frame(ddsRes3[which(ddsRes3$padj<0.05),])
#add annotation
ddsRes3_0.05$Family <- rownames(ddsRes3_0.05)
df['drought.T3_vs_control','total']<- nrow(ddsRes3_0.05)
df['drought.T3_vs_control','positive']<- nrow(ddsRes3_0.05[ddsRes3_0.05$log2FoldChange>0,])
df['drought.T3_vs_control','negative']<- nrow(ddsRes3_0.05[ddsRes3_0.05$log2FoldChange<0,])

### drought T4 vs control
res <- results(dds, alpha = 0.05, name = "treatment_drought.T4_vs_control")
ddsRes4 = lfcShrink(dds, coef = "treatment_drought.T4_vs_control", type="apeglm", res = res)
#subset to padj < 0.05
ddsRes4_0.05 = as.data.frame(ddsRes4[which(ddsRes4$padj<0.05),])
#add annotation
ddsRes4_0.05$Family <- rownames(ddsRes4_0.05)

df['drought.T4_vs_control','total']<- nrow(ddsRes4_0.05)
df['drought.T4_vs_control','positive']<- nrow(ddsRes4_0.05[ddsRes4_0.05$log2FoldChange>0,])
df['drought.T4_vs_control','negative']<- nrow(ddsRes4_0.05[ddsRes4_0.05$log2FoldChange<0,])


### drought T5 vs control
res <- results(dds, alpha = 0.05, name = "treatment_drought.T5_vs_control")
ddsRes5 = lfcShrink(dds, coef = "treatment_drought.T5_vs_control", type="apeglm",res = res)
#subset to padj < 0.05
ddsRes5_0.05 = as.data.frame(ddsRes5[which(ddsRes5$padj<0.05),])
#add annotation
ddsRes5_0.05$Family <- rownames(ddsRes5_0.05)

df['drought.T5_vs_control','total']<- nrow(ddsRes5_0.05)
df['drought.T5_vs_control','positive']<- nrow(ddsRes5_0.05[ddsRes5_0.05$log2FoldChange>0,])
df['drought.T5_vs_control','negative']<- nrow(ddsRes5_0.05[ddsRes5_0.05$log2FoldChange<0,])


### rewetting vs control
res <- results(dds, alpha = 0.05, name = "treatment_rewetting_vs_control")
ddsRes6 = lfcShrink(dds, coef = "treatment_rewetting_vs_control", type="apeglm", res = res)
#subset to padj < 0.05
ddsRes6_0.05 = as.data.frame(ddsRes6[which(ddsRes6$padj<0.05),])
#add annotation
ddsRes6_0.05$Family <- rownames(ddsRes6_0.05)

df['rewetting_vs_control','total']<- nrow(ddsRes6_0.05)
df['rewetting_vs_control','positive']<- nrow(ddsRes6_0.05[ddsRes6_0.05$log2FoldChange>0,])
df['rewetting_vs_control','negative']<- nrow(ddsRes6_0.05[ddsRes6_0.05$log2FoldChange<0,])


### make combined data frame for all treatments with lfc and annotation
genes <- unique(c(rownames(ddsRes1_0.05),rownames(ddsRes2_0.05),rownames(ddsRes3_0.05),
                  rownames(ddsRes4_0.05),rownames(ddsRes5_0.05),rownames(ddsRes6_0.05)))

#create table with baseMeans for genes with sign. LFC in any of the treatment; 
#include only the treatments where sign. LFCs were found
bm <- rbind(ddsRes2_0.05, ddsRes3_0.05,ddsRes4_0.05, ddsRes5_0.05, ddsRes6_0.05)
bm <- unique(bm[,c("Family","baseMean")])


ddsResAll_0.05 <- data.frame()
for (i in genes){
  ddsResAll_0.05[i,"drought_T1"] <- ifelse(i%in%rownames(ddsRes1_0.05),ddsRes1_0.05[i,"log2FoldChange"],NA)
  ddsResAll_0.05[i,"drought_T2"] <- ifelse(i%in%rownames(ddsRes2_0.05),ddsRes2_0.05[i,"log2FoldChange"],NA)
  ddsResAll_0.05[i,"drought_T3"] <- ifelse(i%in%rownames(ddsRes3_0.05),ddsRes3_0.05[i,"log2FoldChange"],NA)
  ddsResAll_0.05[i,"drought_T4"] <- ifelse(i%in%rownames(ddsRes4_0.05),ddsRes4_0.05[i,"log2FoldChange"],NA)
  ddsResAll_0.05[i,"drought_T5"] <- ifelse(i%in%rownames(ddsRes5_0.05),ddsRes5_0.05[i,"log2FoldChange"],NA)
  ddsResAll_0.05[i,"rewetting"] <- ifelse(i%in%rownames(ddsRes6_0.05),ddsRes6_0.05[i,"log2FoldChange"],NA)
  ddsResAll_0.05[i,"Family"] <- i
}


#reorder alphabetically
ddsResAll_0.05 <- ddsResAll_0.05[order(ddsResAll_0.05$Family),]

# transform to long format
ddsResAll_0.05 <- melt(ddsResAll_0.05,id.vars = "Family")

# reorder level of family according to category
ddsResAll_0.05$Family <- factor(ddsResAll_0.05$Family, levels = rev(unique(ddsResAll_0.05$Family[order(ddsResAll_0.05$Family)])))

p <- ggplot(ddsResAll_0.05,aes(variable,Family,fill=value))+
  geom_tile(color= "white",size=0.1) +
  geom_text(aes(label = round(value,digits=2)),size = 3)+
  scale_fill_gradient2(low = "blue4", mid = "white", high = "goldenrod2", midpoint = 0, space = "Lab",
                       na.value = "grey60", guide = "colourbar",aesthetics = "fill", name="LFC relative to control")+
  plot.theme1+
  theme(axis.text.x = element_text(angle = 60, hjust = 1)) +
  geom_point(data = bm,aes(x="Abundance",y=Family,size=baseMean),inherit.aes = F)

p=set_panel_size(p,margin = unit(0, "mm"), width = unit(2.5, "inch"), height = unit(length(unique(ddsResAll_0.05$Family))/5, "inch"))

grid.arrange(p)
```

```
# number of sign. LFCs per treatment
df$treatment <- rownames(df)
df <- melt(df)
df$treatment <- gsub('_.*','',df$treatment)
p <- ggplot(df,aes(x=treatment,y=value,color=variable,group=variable)) + geom_point() +
  geom_line() +
  plot.theme1 + 
  theme(axis.text.x = element_text(angle = 60, hjust = 1)) +
  scale_color_manual(values = c("grey20","goldenrod2","blue4"))
p <- set_panel_size(p,margin = unit(0, "mm"), width = unit(2.5, "inch"), height = unit(2.5, "inch"))

grid.arrange(p)
```

```
rm(list=ls(pattern = "dds"))
rm(p,countID,anno,genes,bm,df)
```

- normalized by mrna content: normalization done as for SEED: DESeq
  size factor divided by mrna content, then DESeq run with predefined size
  factor

```
## [1] TRUE
```

```
## [1] TRUE
```

### Ncyc

```
#### Import count table
count <- read.csv("../input/contigAbundanceAll.tsv", sep = "\t",row.names = 1) 
colnames(count) <- gsub("^X","",colnames(count))
# remove samples with too few reads (sample 6: no reads mapped; sample 19: about 300 reads mapped) and reorder count data according to sample sheet
count <- count[,rownames(sample)]

#### Import annotation tables
# Ncyc
Ncyc <- read.csv("../input/NcycFinal.csv", sep = ",",row.names = 1)
#replace NA with "unclassified"
for (i in 2:7){
  Ncyc[,i][is.na(Ncyc[,i])] <- "unclassified"
}
# remove spaces in beginning of lvl1and lvl2
Ncyc$lvl1 <- gsub("^ ","",Ncyc$lvl1)
Ncyc$lvl2 <- gsub("^ ","",Ncyc$lvl2)

# duplicate contigs in count table if there are multiple annotations such that rownames of count table match annotation table

for (i in rownames(Ncyc)[!(rownames(Ncyc)%in%rownames(count))]){
  count[i,1:26] <- count[rownames(count)==Ncyc[i,"contig"],1:26]
}
```

- Deseq based raw data, aggregated at lvl2

```
#subset count table and annotation table to contigs annotated with Ncyc
NcycAnno <- Ncyc[Ncyc$NcycID!="unclassified",]
countAnno <- count[rownames(NcycAnno),]

all(colnames(countAnno)==sample$sampleID)
```

```
## [1] TRUE
```

```
# aggregate at lvl2 (gene)
countLvl2 <- AbuTableInteger(countab = countAnno, taxo = NcycAnno, col2matchcount = rownames(countAnno),
                             col2matchtax = rownames(NcycAnno), Taxlevel = "lvl2", Samp = sample, fac = "sampleID")

#remove sample data
countLvl2 <- countLvl2[,-(48:69)]

#transpose
countLvl2 <- as.data.frame(t(countLvl2))

#create Deseq object
all(colnames(countLvl2)==rownames(sample))
```

```
## [1] TRUE
```

```
dds <- DESeqDataSetFromMatrix(countData = countLvl2, colData = sample, design = ~treatment) 

# Run Deseq
dds <- DESeq(dds, test="Wald", fitType="local") 


### Extract all comparisons with the control using shrinkage with apeglm, for padj <0.05

#dataframe for number of sign. LFCs
df <- data.frame()

### drought T1 vs control
res <- results(dds, alpha = 0.05, name = "treatment_drought.T1_vs_control")
ddsRes1 = lfcShrink(dds, coef = "treatment_drought.T1_vs_control", type="apeglm", res = res)
#subset to padj < 0.05
ddsRes1_0.05 = as.data.frame(ddsRes1[which(ddsRes1$padj<0.05),])
# no sign. lfc

df['drought.T1_vs_control','total']<- nrow(ddsRes1_0.05)
df['drought.T1_vs_control','positive']<- nrow(ddsRes1_0.05[ddsRes1_0.05$log2FoldChange>0,])
df['drought.T1_vs_control','negative']<- nrow(ddsRes1_0.05[ddsRes1_0.05$log2FoldChange<0,])

### drought T2 vs control
res <- results(dds, alpha = 0.05, name = "treatment_drought.T2_vs_control")
ddsRes2 = lfcShrink(dds, coef = "treatment_drought.T2_vs_control", type="apeglm",res = res)
#subset to padj < 0.05
ddsRes2_0.05 = as.data.frame(ddsRes2[which(ddsRes2$padj<0.05),])
# no sign. LFC at p< 0.05
df['drought.T2_vs_control','total']<- nrow(ddsRes2_0.05)
df['drought.T2_vs_control','positive']<- nrow(ddsRes2_0.05[ddsRes2_0.05$log2FoldChange>0,])
df['drought.T2_vs_control','negative']<- nrow(ddsRes2_0.05[ddsRes2_0.05$log2FoldChange<0,])

### drought T3 vs control
res <- results(dds, alpha = 0.05, name = "treatment_drought.T3_vs_control")
ddsRes3 = lfcShrink(dds, coef = "treatment_drought.T3_vs_control", type="apeglm", res = res)
#subset to padj < 0.05
ddsRes3_0.05 = as.data.frame(ddsRes3[which(ddsRes3$padj<0.05),])

#add annotation
a <- list()
for(i in rownames(ddsRes3_0.05)){
  a[[i]] <- data.frame()
  for (j in 1:length(unique(paste(Ncyc[Ncyc$lvl2==i,"lvl1"],i)))){
    a[[i]][j,colnames(ddsRes3_0.05)] <- ddsRes3_0.05[i,colnames(ddsRes3_0.05)]
    a[[i]][j,"annotation"] <- unique(paste(Ncyc[Ncyc$lvl2==i,"lvl1"],i,sep=";"))[j]
  }
}
#combine
ddsRes3_0.05 <- do.call(rbind,a)
# separate annotation
library(tidyr)
ddsRes3_0.05 <- separate(data=ddsRes3_0.05,col=annotation, into = c("lvl1","lvl2"),sep = ";",remove = F)
rownames(ddsRes3_0.05) <- make.unique(ddsRes3_0.05[,"lvl2"], sep=".")

df['drought.T3_vs_control','total']<- nrow(ddsRes3_0.05)
df['drought.T3_vs_control','positive']<- nrow(ddsRes3_0.05[ddsRes3_0.05$log2FoldChange>0,])
df['drought.T3_vs_control','negative']<- nrow(ddsRes3_0.05[ddsRes3_0.05$log2FoldChange<0,])

### drought T4 vs control
res <- results(dds, alpha = 0.05, name = "treatment_drought.T4_vs_control")
ddsRes4 = lfcShrink(dds, coef = "treatment_drought.T4_vs_control", type="apeglm", res = res)
#subset to padj < 0.05
ddsRes4_0.05 = as.data.frame(ddsRes4[which(ddsRes4$padj<0.05),])

#add annotation
a <- list()
for(i in rownames(ddsRes4_0.05)){
  a[[i]] <- data.frame()
  for (j in 1:length(unique(paste(Ncyc[Ncyc$lvl2==i,"lvl1"],i)))){
    a[[i]][j,colnames(ddsRes4_0.05)] <- ddsRes4_0.05[i,colnames(ddsRes4_0.05)]
    a[[i]][j,"annotation"] <- unique(paste(Ncyc[Ncyc$lvl2==i,"lvl1"],i,sep=";"))[j]
  }
}
#combine
ddsRes4_0.05 <- do.call(rbind,a)
# separate annotation
ddsRes4_0.05 <- separate(data=ddsRes4_0.05,col=annotation, into = c("lvl1","lvl2"),sep = ";",remove = F)
rownames(ddsRes4_0.05) <- make.unique(ddsRes4_0.05[,"lvl2"], sep=".")

df['drought.T4_vs_control','total']<- nrow(ddsRes4_0.05)
df['drought.T4_vs_control','positive']<- nrow(ddsRes4_0.05[ddsRes4_0.05$log2FoldChange>0,])
df['drought.T4_vs_control','negative']<- nrow(ddsRes4_0.05[ddsRes4_0.05$log2FoldChange<0,])

### drought T5 vs control
res <- results(dds, alpha = 0.05, name = "treatment_drought.T5_vs_control")
ddsRes5 = lfcShrink(dds, coef = "treatment_drought.T5_vs_control", type="apeglm", res =res)
#subset to padj < 0.05
ddsRes5_0.05 = as.data.frame(ddsRes5[which(ddsRes5$padj<0.05),])

#add annotation
a <- list()
for(i in rownames(ddsRes5_0.05)){
  a[[i]] <- data.frame()
  for (j in 1:length(unique(paste(Ncyc[Ncyc$lvl2==i,"lvl1"],i)))){
    a[[i]][j,colnames(ddsRes5_0.05)] <- ddsRes5_0.05[i,colnames(ddsRes5_0.05)]
    a[[i]][j,"annotation"] <- unique(paste(Ncyc[Ncyc$lvl2==i,"lvl1"],i,sep=";"))[j]
  }
}
#combine
ddsRes5_0.05 <- do.call(rbind,a)
# separate annotation
ddsRes5_0.05 <- separate(data=ddsRes5_0.05,col=annotation, into = c("lvl1","lvl2"),sep = ";",remove = F)
rownames(ddsRes5_0.05) <- make.unique(ddsRes5_0.05[,"lvl2"], sep=".")

df['drought.T5_vs_control','total']<- nrow(ddsRes5_0.05)
df['drought.T5_vs_control','positive']<- nrow(ddsRes5_0.05[ddsRes5_0.05$log2FoldChange>0,])
df['drought.T5_vs_control','negative']<- nrow(ddsRes5_0.05[ddsRes5_0.05$log2FoldChange<0,])


### rewetting vs control
res <- results(dds, alpha = 0.05, name = "treatment_rewetting_vs_control")
ddsRes6 = lfcShrink(dds, coef = "treatment_rewetting_vs_control", type="apeglm",res = res)
#subset to padj < 0.05
ddsRes6_0.05 = as.data.frame(ddsRes6[which(ddsRes6$padj<0.05),])

#add annotation
a <- list()
for(i in rownames(ddsRes6_0.05)){
  a[[i]] <- data.frame()
  for (j in 1:length(unique(paste(Ncyc[Ncyc$lvl2==i,"lvl1"],i)))){
    a[[i]][j,colnames(ddsRes6_0.05)] <- ddsRes6_0.05[i,colnames(ddsRes6_0.05)]
    a[[i]][j,"annotation"] <- unique(paste(Ncyc[Ncyc$lvl2==i,"lvl1"],i,sep=";"))[j]
  }
}
#combine
ddsRes6_0.05 <- do.call(rbind,a)
# separate annotation
ddsRes6_0.05 <- separate(data=ddsRes6_0.05,col=annotation, into = c("lvl1","lvl2"),sep = ";",remove = F)
rownames(ddsRes6_0.05) <- make.unique(ddsRes6_0.05[,"lvl2"], sep=".")

df['rewetting_vs_control','total']<- nrow(ddsRes6_0.05)
df['rewetting_vs_control','positive']<- nrow(ddsRes6_0.05[ddsRes6_0.05$log2FoldChange>0,])
df['rewetting_vs_control','negative']<- nrow(ddsRes6_0.05[ddsRes6_0.05$log2FoldChange<0,])


### make combined data frame for all treatments with lfc and annotation
genes <- unique(c(rownames(ddsRes1_0.05),rownames(ddsRes2_0.05),rownames(ddsRes3_0.05),
                  rownames(ddsRes4_0.05),rownames(ddsRes5_0.05),rownames(ddsRes6_0.05)))

#create table with annotations for genes with sign. LFC in any of the treatment; and basemeans
#include only the treatments where sign. LFCs were found
anno <- unique(rbind(ddsRes3_0.05[,c("lvl1","lvl2","baseMean")],ddsRes4_0.05[,c("lvl1","lvl2","baseMean")],
                     ddsRes5_0.05[,c("lvl1","lvl2","baseMean")],ddsRes6_0.05[,c("lvl1","lvl2","baseMean")]))
anno[,"lvl2unique"] <- rownames(anno)

ddsResAll_0.05 <- data.frame()
for (i in genes){
  ddsResAll_0.05[i,"drought_T1"] <- ifelse(i%in%rownames(ddsRes1_0.05),ddsRes1_0.05[i,"log2FoldChange"],NA)
  ddsResAll_0.05[i,"drought_T2"] <- ifelse(i%in%rownames(ddsRes2_0.05),ddsRes2_0.05[i,"log2FoldChange"],NA)
  ddsResAll_0.05[i,"drought_T3"] <- ifelse(i%in%rownames(ddsRes3_0.05),ddsRes3_0.05[i,"log2FoldChange"],NA)
  ddsResAll_0.05[i,"drought_T4"] <- ifelse(i%in%rownames(ddsRes4_0.05),ddsRes4_0.05[i,"log2FoldChange"],NA)
  ddsResAll_0.05[i,"drought_T5"] <- ifelse(i%in%rownames(ddsRes5_0.05),ddsRes5_0.05[i,"log2FoldChange"],NA)
  ddsResAll_0.05[i,"rewetting"] <- ifelse(i%in%rownames(ddsRes6_0.05),ddsRes6_0.05[i,"log2FoldChange"],NA)
  ddsResAll_0.05[i,c("lvl1","lvl2")] <- anno[i,c("lvl1","lvl2")]
}

# add a columns with the rownmaes to distiniguish genes with duplicate annotaitons
ddsResAll_0.05[,"lvl2unique"] <- rownames(ddsResAll_0.05)
#reorder according to lvl1
ddsResAll_0.05 <- ddsResAll_0.05[order(ddsResAll_0.05$lvl1),]


# transform to long format
ddsResAll_0.05 <- melt(ddsResAll_0.05,id.vars = c("lvl1","lvl2","lvl2unique"))

# reorder level of genes according to category
ddsResAll_0.05$lvl2unique <- factor(ddsResAll_0.05$lvl2unique, levels = rev(unique(ddsResAll_0.05$lvl2unique[order(ddsResAll_0.05$lvl1)])))

p <- ggplot(ddsResAll_0.05,aes(variable,lvl2unique,fill=value))+
  geom_tile(color= "white",size=0.1) +
  geom_text(aes(label = round(value,digits=2)),size = 3)+
  scale_fill_gradient2(low = "blue4", mid = "white", high = "goldenrod2", midpoint = 0, space = "Lab",
                       na.value = "grey60", guide = "colourbar",aesthetics = "fill", name="LFC relative to control")+
  plot.theme1+
  theme(axis.text.x = element_text(angle = 60, hjust = 1)) +
  geom_point(data = anno,aes(x="Abundance",y=lvl2unique,size=baseMean),inherit.aes = F)

p=set_panel_size(p,margin = unit(0, "mm"), width = unit(2.5, "inch"), height = unit(length(unique(ddsResAll_0.05$lvl2unique))/5, "inch"))

grid.arrange(p)
```

```
# plot with number of sign. LFCs
df$treatment <- rownames(df)
df <- melt(df)
df$treatment <- gsub('_.*','',df$treatment)
p <- ggplot(df,aes(x=treatment,y=value,color=variable,group=variable)) + geom_point() +
  geom_line() +
  plot.theme1 + 
  theme(axis.text.x = element_text(angle = 60, hjust = 1)) +
  scale_color_manual(values = c("grey20","goldenrod2","blue4"))
p <- set_panel_size(p,margin = unit(0, "mm"), width = unit(2.5, "inch"), height = unit(2.5, "inch"))


grid.arrange(p)
```

```
rm(list=ls(pattern = "dds"))
rm(p,countLvl2,anno,genes)
```

- normalized to RNA content weighted by mrna fraction (normalization
  as for SEED and CAZy, DESeq analysis same as for raw counts)

```
## [1] TRUE
```

```
## [1] TRUE
```

```
## [1] TRUE
```

### rRNA genes (taxonomic groups at phylum level)

```
# create phyloseq object for sample data
phySample <- sample_data(sample)
#### Import count table
count <- read.csv("../input/otu.csv", sep = ",",row.names = 1) 

#  samples with too few reads (sample 6: no reads mapped; sample 19: about 300 reads mapped) are already removed in input file
# adjust column names of the count table to match the sample table
colnames(count) <- paste(gsub("S","",colnames(count)),
                         colnames(count),
                         "nonrRNA", sep = "_")

all(colnames(count)==rownames(sample))
```

```
## [1] TRUE
```

```
# create phyloseq object
phycount <- otu_table(count,taxa_are_rows = T)


#### Import taxonomy table
tax <- read.csv("../input/tax.csv", sep = ",",row.names = 1) 
tax <- as.matrix(tax)

# create phyloseq object
phytax <- tax_table(tax)

phy <- merge_phyloseq(phySample,phycount,phytax)

# create object for prokaryotes and eukaryotes

phyPro <- prune_taxa(phy@tax_table@.Data[,"Domain"]%in%c("Bacteria",   "Archaea"),phy)
phyEu <-prune_taxa(phy@tax_table@.Data[,"Domain"]=="Eukaryota" ,phy)
```

#### Prokaryotes

- raw counts

```
# count table for prokaryotes
countP <- as.data.frame(otu_table(phyPro))
taxP <- as.data.frame(tax_table(phyPro))
sample$sampleID <- rownames(sample)
all(colnames(countP)==sample$sampleID)
```

```
## [1] TRUE
```

```
# aggregate at lvl2 (gene)
countPAgg <- AbuTableInteger(countab = countP, taxo = taxP, col2matchcount = rownames(countP),
                             col2matchtax = rownames(taxP), Taxlevel = "Phylum", Samp = sample, fac = "sampleID")

#remove sample data
countPAgg <- countPAgg[,-(40:62)]

#transpose
countPAgg <- as.data.frame(t(countPAgg))

#create Deseq object
all(colnames(countPAgg)==rownames(sample))
```

```
## [1] TRUE
```

```
dds <- DESeqDataSetFromMatrix(countData = countPAgg, colData = sample, design = ~treatment)
```

```
## converting counts to integer mode
```

```
##   Note: levels of factors in the design contain characters other than
##   letters, numbers, '_' and '.'. It is recommended (but not required) to use
##   only letters, numbers, and delimiters '_' or '.', as these are safe characters
##   for column names in R. [This is a message, not a warning or an error]
```

```
# Run Deseq
dds <- DESeq(dds, test="Wald", fitType="local")
```

```
## estimating size factors
##   Note: levels of factors in the design contain characters other than
##   letters, numbers, '_' and '.'. It is recommended (but not required) to use
##   only letters, numbers, and delimiters '_' or '.', as these are safe characters
##   for column names in R. [This is a message, not a warning or an error]
```

```
## estimating dispersions
```

```
## gene-wise dispersion estimates
```

```
## mean-dispersion relationship
```

```
##   Note: levels of factors in the design contain characters other than
##   letters, numbers, '_' and '.'. It is recommended (but not required) to use
##   only letters, numbers, and delimiters '_' or '.', as these are safe characters
##   for column names in R. [This is a message, not a warning or an error]
```

```
## final dispersion estimates
```

```
## fitting model and testing
```

```
### Extract all comparisons with the control using shrinkage with apeglm

#dataframe for number of sign. LFCs
df <- data.frame()

### drought T1 vs control
res <- results(dds, alpha = 0.05, name = "treatment_drought.T1_vs_control")
ddsRes1 = lfcShrink(dds, coef = "treatment_drought.T1_vs_control", type="apeglm", res = res)
```

```
## using 'apeglm' for LFC shrinkage. If used in published research, please cite:
##     Zhu, A., Ibrahim, J.G., Love, M.I. (2018) Heavy-tailed prior distributions for
##     sequence count data: removing the noise and preserving large differences.
##     Bioinformatics. https://doi.org/10.1093/bioinformatics/bty895
```

```
#subset to padj < 0.05
ddsRes1_0.05 = as.data.frame(ddsRes1[which(ddsRes1$padj<0.05),])
# no sign. lfc

df['drought.T1_vs_control','total']<- nrow(ddsRes1_0.05)
df['drought.T1_vs_control','positive']<- nrow(ddsRes1_0.05[ddsRes1_0.05$log2FoldChange>0,])
df['drought.T1_vs_control','negative']<- nrow(ddsRes1_0.05[ddsRes1_0.05$log2FoldChange<0,])

### drought T2 vs control
res <- results(dds, alpha = 0.05, name = "treatment_drought.T2_vs_control")
ddsRes2 = lfcShrink(dds, coef = "treatment_drought.T2_vs_control", type="apeglm",res = res)
```

```
## using 'apeglm' for LFC shrinkage. If used in published research, please cite:
##     Zhu, A., Ibrahim, J.G., Love, M.I. (2018) Heavy-tailed prior distributions for
##     sequence count data: removing the noise and preserving large differences.
##     Bioinformatics. https://doi.org/10.1093/bioinformatics/bty895
```

```
#subset to padj < 0.05
ddsRes2_0.05 = as.data.frame(ddsRes2[which(ddsRes2$padj<0.05),])
# no sign. LFC at p< 0.05
df['drought.T2_vs_control','total']<- nrow(ddsRes2_0.05)
df['drought.T2_vs_control','positive']<- nrow(ddsRes2_0.05[ddsRes2_0.05$log2FoldChange>0,])
df['drought.T2_vs_control','negative']<- nrow(ddsRes2_0.05[ddsRes2_0.05$log2FoldChange<0,])

### drought T3 vs control
res <- results(dds, alpha = 0.05, name = "treatment_drought.T3_vs_control")
ddsRes3 = lfcShrink(dds, coef = "treatment_drought.T3_vs_control", type="apeglm", res = res)
```

```
## using 'apeglm' for LFC shrinkage. If used in published research, please cite:
##     Zhu, A., Ibrahim, J.G., Love, M.I. (2018) Heavy-tailed prior distributions for
##     sequence count data: removing the noise and preserving large differences.
##     Bioinformatics. https://doi.org/10.1093/bioinformatics/bty895
```

```
#subset to padj < 0.05
ddsRes3_0.05 = as.data.frame(ddsRes3[which(ddsRes3$padj<0.05),])

df['drought.T3_vs_control','total']<- nrow(ddsRes3_0.05)
df['drought.T3_vs_control','positive']<- nrow(ddsRes3_0.05[ddsRes3_0.05$log2FoldChange>0,])
df['drought.T3_vs_control','negative']<- nrow(ddsRes3_0.05[ddsRes3_0.05$log2FoldChange<0,])

### drought T4 vs control
res <- results(dds, alpha = 0.05, name = "treatment_drought.T4_vs_control")
ddsRes4 = lfcShrink(dds, coef = "treatment_drought.T4_vs_control", type="apeglm", res = res)
```

```
## using 'apeglm' for LFC shrinkage. If used in published research, please cite:
##     Zhu, A., Ibrahim, J.G., Love, M.I. (2018) Heavy-tailed prior distributions for
##     sequence count data: removing the noise and preserving large differences.
##     Bioinformatics. https://doi.org/10.1093/bioinformatics/bty895
```

```
#subset to padj < 0.05
ddsRes4_0.05 = as.data.frame(ddsRes4[which(ddsRes4$padj<0.05),])


df['drought.T4_vs_control','total']<- nrow(ddsRes4_0.05)
df['drought.T4_vs_control','positive']<- nrow(ddsRes4_0.05[ddsRes4_0.05$log2FoldChange>0,])
df['drought.T4_vs_control','negative']<- nrow(ddsRes4_0.05[ddsRes4_0.05$log2FoldChange<0,])

### drought T5 vs control
res <- results(dds, alpha = 0.05, name = "treatment_drought.T5_vs_control")
ddsRes5 = lfcShrink(dds, coef = "treatment_drought.T5_vs_control", type="apeglm", res =res)
```

```
## using 'apeglm' for LFC shrinkage. If used in published research, please cite:
##     Zhu, A., Ibrahim, J.G., Love, M.I. (2018) Heavy-tailed prior distributions for
##     sequence count data: removing the noise and preserving large differences.
##     Bioinformatics. https://doi.org/10.1093/bioinformatics/bty895
```

```
#subset to padj < 0.05
ddsRes5_0.05 = as.data.frame(ddsRes5[which(ddsRes5$padj<0.05),])


df['drought.T5_vs_control','total']<- nrow(ddsRes5_0.05)
df['drought.T5_vs_control','positive']<- nrow(ddsRes5_0.05[ddsRes5_0.05$log2FoldChange>0,])
df['drought.T5_vs_control','negative']<- nrow(ddsRes5_0.05[ddsRes5_0.05$log2FoldChange<0,])


### rewetting vs control
res <- results(dds, alpha = 0.05, name = "treatment_rewetting_vs_control")
ddsRes6 = lfcShrink(dds, coef = "treatment_rewetting_vs_control", type="apeglm",res = res)
```

```
## using 'apeglm' for LFC shrinkage. If used in published research, please cite:
##     Zhu, A., Ibrahim, J.G., Love, M.I. (2018) Heavy-tailed prior distributions for
##     sequence count data: removing the noise and preserving large differences.
##     Bioinformatics. https://doi.org/10.1093/bioinformatics/bty895
```

```
#subset to padj < 0.05
ddsRes6_0.05 = as.data.frame(ddsRes6[which(ddsRes6$padj<0.05),])


df['rewetting_vs_control','total']<- nrow(ddsRes6_0.05)
df['rewetting_vs_control','positive']<- nrow(ddsRes6_0.05[ddsRes6_0.05$log2FoldChange>0,])
df['rewetting_vs_control','negative']<- nrow(ddsRes6_0.05[ddsRes6_0.05$log2FoldChange<0,])


### make combined data frame for all treatments with lfc and annotation
phylum <- unique(c(rownames(ddsRes1_0.05),rownames(ddsRes2_0.05),rownames(ddsRes3_0.05),
                   rownames(ddsRes4_0.05),rownames(ddsRes5_0.05),rownames(ddsRes6_0.05)))


ddsResAll_0.05 <- data.frame()
for (i in phylum){
  ddsResAll_0.05[i,"drought_T1"] <- ifelse(i%in%rownames(ddsRes1_0.05),ddsRes1_0.05[i,"log2FoldChange"],NA)
  ddsResAll_0.05[i,"drought_T2"] <- ifelse(i%in%rownames(ddsRes2_0.05),ddsRes2_0.05[i,"log2FoldChange"],NA)
  ddsResAll_0.05[i,"drought_T3"] <- ifelse(i%in%rownames(ddsRes3_0.05),ddsRes3_0.05[i,"log2FoldChange"],NA)
  ddsResAll_0.05[i,"drought_T4"] <- ifelse(i%in%rownames(ddsRes4_0.05),ddsRes4_0.05[i,"log2FoldChange"],NA)
  ddsResAll_0.05[i,"drought_T5"] <- ifelse(i%in%rownames(ddsRes5_0.05),ddsRes5_0.05[i,"log2FoldChange"],NA)
  ddsResAll_0.05[i,"rewetting"] <- ifelse(i%in%rownames(ddsRes6_0.05),ddsRes6_0.05[i,"log2FoldChange"],NA)
  ddsResAll_0.05[i,"phylum"] <-i
}


# transform to long format
ddsResAll_0.05 <- melt(ddsResAll_0.05, id.vars = c("phylum"))

# combined table with abundance (base mean)
# add column with phylum to each results data frame
ddsRes1_0.05[,"phylum"] <- rownames(ddsRes1_0.05)
ddsRes2_0.05[,"phylum"] <- rownames(ddsRes2_0.05)
ddsRes3_0.05[,"phylum"] <- rownames(ddsRes3_0.05)
ddsRes4_0.05[,"phylum"] <- rownames(ddsRes4_0.05)
ddsRes5_0.05[,"phylum"] <- rownames(ddsRes5_0.05)
ddsRes6_0.05[,"phylum"] <- rownames(ddsRes6_0.05)

ddsResAll_0.05.bm <- do.call(rbind,list(ddsRes1_0.05,ddsRes2_0.05,ddsRes3_0.05,ddsRes4_0.05,ddsRes5_0.05,ddsRes6_0.05))
ddsResAll_0.05.bm <- ddsResAll_0.05.bm[!duplicated(ddsResAll_0.05.bm$baseMean),]

p <- ggplot(ddsResAll_0.05,aes(variable,phylum,fill=value))+
  geom_tile(color= "white",size=0.1) +
  geom_text(aes(label = round(value,digits=2)),size = 3)+
  scale_fill_gradient2(low = "blue4", mid = "white", high = "goldenrod2", midpoint = 0, space = "Lab",
                       na.value = "grey60", guide = "colourbar",aesthetics = "fill", name="LFC relative to control")+
  plot.theme1+
  theme(axis.text.x = element_text(angle = 60, hjust = 1)) +
  geom_point(data = ddsResAll_0.05.bm,aes(x="Abundance",y=phylum,size=baseMean),inherit.aes = F)

p=set_panel_size(p,margin = unit(0, "mm"), width = unit(2.5, "inch"), height = unit(length(unique(ddsResAll_0.05$phylum))/5, "inch"))


grid.arrange(p)
```

```
# number of sign. LFCs
df$treatment <- rownames(df)
df <- melt(df)
```

```
## Using treatment as id variables
```

```
df$treatment <- gsub('_.*','',df$treatment)
p <- ggplot(df,aes(x=treatment,y=value,color=variable,group=variable)) + geom_point() +
  geom_line() +
  plot.theme1 + 
  theme(axis.text.x = element_text(angle = 60, hjust = 1)) +
  scale_color_manual(values = c("grey20","goldenrod2","blue4"))
p <- set_panel_size(p,margin = unit(0, "mm"), width = unit(2.5, "inch"), height = unit(2.5, "inch"))


grid.arrange(p)
```

```
rm(list=ls(pattern = "dds"))
```

- normalized to RNA content weighted by rrna fraction, (normalization
  same as for functional gene databases, but rrna fraction instead of mrna
  fraction used, following DESeq analyses are the same as for raw
  counts)

```
# add column with RNA content normlalized by rrna fraction
#calculate fraction rrna from total rna
sample$rrna_rat <- sample$rrna/(sample$mrna+sample$rrna)

#weight RNA content by mrna fraction
sample$RNA_DW_rrna <- sample$RNA_DW*sample$rrna_rat

# scale relative to mean
sample$RNA_DW_rrna_sc <- sample$RNA_DW_rrna/mean(sample$RNA_DW_rrna)
```

```
## [1] TRUE
```

```
## [1] TRUE
```

```
## converting counts to integer mode
```

```
##   Note: levels of factors in the design contain characters other than
##   letters, numbers, '_' and '.'. It is recommended (but not required) to use
##   only letters, numbers, and delimiters '_' or '.', as these are safe characters
##   for column names in R. [This is a message, not a warning or an error]
##   Note: levels of factors in the design contain characters other than
##   letters, numbers, '_' and '.'. It is recommended (but not required) to use
##   only letters, numbers, and delimiters '_' or '.', as these are safe characters
##   for column names in R. [This is a message, not a warning or an error]
```

```
## [1] TRUE
```

```
##   Note: levels of factors in the design contain characters other than
##   letters, numbers, '_' and '.'. It is recommended (but not required) to use
##   only letters, numbers, and delimiters '_' or '.', as these are safe characters
##   for column names in R. [This is a message, not a warning or an error]
```

```
## using pre-existing size factors
```

```
## estimating dispersions
```

```
## gene-wise dispersion estimates
```

```
## mean-dispersion relationship
```

```
##   Note: levels of factors in the design contain characters other than
##   letters, numbers, '_' and '.'. It is recommended (but not required) to use
##   only letters, numbers, and delimiters '_' or '.', as these are safe characters
##   for column names in R. [This is a message, not a warning or an error]
```

```
## final dispersion estimates
```

```
## fitting model and testing
```

```
## using 'apeglm' for LFC shrinkage. If used in published research, please cite:
##     Zhu, A., Ibrahim, J.G., Love, M.I. (2018) Heavy-tailed prior distributions for
##     sequence count data: removing the noise and preserving large differences.
##     Bioinformatics. https://doi.org/10.1093/bioinformatics/bty895
## using 'apeglm' for LFC shrinkage. If used in published research, please cite:
##     Zhu, A., Ibrahim, J.G., Love, M.I. (2018) Heavy-tailed prior distributions for
##     sequence count data: removing the noise and preserving large differences.
##     Bioinformatics. https://doi.org/10.1093/bioinformatics/bty895
## using 'apeglm' for LFC shrinkage. If used in published research, please cite:
##     Zhu, A., Ibrahim, J.G., Love, M.I. (2018) Heavy-tailed prior distributions for
##     sequence count data: removing the noise and preserving large differences.
##     Bioinformatics. https://doi.org/10.1093/bioinformatics/bty895
## using 'apeglm' for LFC shrinkage. If used in published research, please cite:
##     Zhu, A., Ibrahim, J.G., Love, M.I. (2018) Heavy-tailed prior distributions for
##     sequence count data: removing the noise and preserving large differences.
##     Bioinformatics. https://doi.org/10.1093/bioinformatics/bty895
## using 'apeglm' for LFC shrinkage. If used in published research, please cite:
##     Zhu, A., Ibrahim, J.G., Love, M.I. (2018) Heavy-tailed prior distributions for
##     sequence count data: removing the noise and preserving large differences.
##     Bioinformatics. https://doi.org/10.1093/bioinformatics/bty895
## using 'apeglm' for LFC shrinkage. If used in published research, please cite:
##     Zhu, A., Ibrahim, J.G., Love, M.I. (2018) Heavy-tailed prior distributions for
##     sequence count data: removing the noise and preserving large differences.
##     Bioinformatics. https://doi.org/10.1093/bioinformatics/bty895
```

```
## Using treatment as id variables
```

#### Eukaryotes \* for raw counts (analyses same as for prokaryotes)

```
## [1] TRUE
```

```
## [1] TRUE
```

```
## converting counts to integer mode
```

```
##   Note: levels of factors in the design contain characters other than
##   letters, numbers, '_' and '.'. It is recommended (but not required) to use
##   only letters, numbers, and delimiters '_' or '.', as these are safe characters
##   for column names in R. [This is a message, not a warning or an error]
```

```
## estimating size factors
```

```
##   Note: levels of factors in the design contain characters other than
##   letters, numbers, '_' and '.'. It is recommended (but not required) to use
##   only letters, numbers, and delimiters '_' or '.', as these are safe characters
##   for column names in R. [This is a message, not a warning or an error]
```

```
## estimating dispersions
```

```
## gene-wise dispersion estimates
```

```
## mean-dispersion relationship
```

```
##   Note: levels of factors in the design contain characters other than
##   letters, numbers, '_' and '.'. It is recommended (but not required) to use
##   only letters, numbers, and delimiters '_' or '.', as these are safe characters
##   for column names in R. [This is a message, not a warning or an error]
```

```
## final dispersion estimates
```

```
## fitting model and testing
```

```
## using 'apeglm' for LFC shrinkage. If used in published research, please cite:
##     Zhu, A., Ibrahim, J.G., Love, M.I. (2018) Heavy-tailed prior distributions for
##     sequence count data: removing the noise and preserving large differences.
##     Bioinformatics. https://doi.org/10.1093/bioinformatics/bty895
## using 'apeglm' for LFC shrinkage. If used in published research, please cite:
##     Zhu, A., Ibrahim, J.G., Love, M.I. (2018) Heavy-tailed prior distributions for
##     sequence count data: removing the noise and preserving large differences.
##     Bioinformatics. https://doi.org/10.1093/bioinformatics/bty895
## using 'apeglm' for LFC shrinkage. If used in published research, please cite:
##     Zhu, A., Ibrahim, J.G., Love, M.I. (2018) Heavy-tailed prior distributions for
##     sequence count data: removing the noise and preserving large differences.
##     Bioinformatics. https://doi.org/10.1093/bioinformatics/bty895
## using 'apeglm' for LFC shrinkage. If used in published research, please cite:
##     Zhu, A., Ibrahim, J.G., Love, M.I. (2018) Heavy-tailed prior distributions for
##     sequence count data: removing the noise and preserving large differences.
##     Bioinformatics. https://doi.org/10.1093/bioinformatics/bty895
## using 'apeglm' for LFC shrinkage. If used in published research, please cite:
##     Zhu, A., Ibrahim, J.G., Love, M.I. (2018) Heavy-tailed prior distributions for
##     sequence count data: removing the noise and preserving large differences.
##     Bioinformatics. https://doi.org/10.1093/bioinformatics/bty895
## using 'apeglm' for LFC shrinkage. If used in published research, please cite:
##     Zhu, A., Ibrahim, J.G., Love, M.I. (2018) Heavy-tailed prior distributions for
##     sequence count data: removing the noise and preserving large differences.
##     Bioinformatics. https://doi.org/10.1093/bioinformatics/bty895
```

```
## Using treatment as id variables
```

- normalized by RNA content weigthed by rrna fraction (same analyses
  as for prokaryotes)

```
## [1] TRUE
```

```
## [1] TRUE
```

```
## converting counts to integer mode
```

```
##   Note: levels of factors in the design contain characters other than
##   letters, numbers, '_' and '.'. It is recommended (but not required) to use
##   only letters, numbers, and delimiters '_' or '.', as these are safe characters
##   for column names in R. [This is a message, not a warning or an error]
##   Note: levels of factors in the design contain characters other than
##   letters, numbers, '_' and '.'. It is recommended (but not required) to use
##   only letters, numbers, and delimiters '_' or '.', as these are safe characters
##   for column names in R. [This is a message, not a warning or an error]
```

```
## [1] TRUE
```

```
##   Note: levels of factors in the design contain characters other than
##   letters, numbers, '_' and '.'. It is recommended (but not required) to use
##   only letters, numbers, and delimiters '_' or '.', as these are safe characters
##   for column names in R. [This is a message, not a warning or an error]
```

```
## using pre-existing size factors
```

```
## estimating dispersions
```

```
## gene-wise dispersion estimates
```

```
## mean-dispersion relationship
```

```
##   Note: levels of factors in the design contain characters other than
##   letters, numbers, '_' and '.'. It is recommended (but not required) to use
##   only letters, numbers, and delimiters '_' or '.', as these are safe characters
##   for column names in R. [This is a message, not a warning or an error]
```

```
## final dispersion estimates
```

```
## fitting model and testing
```

```
## using 'apeglm' for LFC shrinkage. If used in published research, please cite:
##     Zhu, A., Ibrahim, J.G., Love, M.I. (2018) Heavy-tailed prior distributions for
##     sequence count data: removing the noise and preserving large differences.
##     Bioinformatics. https://doi.org/10.1093/bioinformatics/bty895
## using 'apeglm' for LFC shrinkage. If used in published research, please cite:
##     Zhu, A., Ibrahim, J.G., Love, M.I. (2018) Heavy-tailed prior distributions for
##     sequence count data: removing the noise and preserving large differences.
##     Bioinformatics. https://doi.org/10.1093/bioinformatics/bty895
## using 'apeglm' for LFC shrinkage. If used in published research, please cite:
##     Zhu, A., Ibrahim, J.G., Love, M.I. (2018) Heavy-tailed prior distributions for
##     sequence count data: removing the noise and preserving large differences.
##     Bioinformatics. https://doi.org/10.1093/bioinformatics/bty895
## using 'apeglm' for LFC shrinkage. If used in published research, please cite:
##     Zhu, A., Ibrahim, J.G., Love, M.I. (2018) Heavy-tailed prior distributions for
##     sequence count data: removing the noise and preserving large differences.
##     Bioinformatics. https://doi.org/10.1093/bioinformatics/bty895
## using 'apeglm' for LFC shrinkage. If used in published research, please cite:
##     Zhu, A., Ibrahim, J.G., Love, M.I. (2018) Heavy-tailed prior distributions for
##     sequence count data: removing the noise and preserving large differences.
##     Bioinformatics. https://doi.org/10.1093/bioinformatics/bty895
## using 'apeglm' for LFC shrinkage. If used in published research, please cite:
##     Zhu, A., Ibrahim, J.G., Love, M.I. (2018) Heavy-tailed prior distributions for
##     sequence count data: removing the noise and preserving large differences.
##     Bioinformatics. https://doi.org/10.1093/bioinformatics/bty895
```

```
## Using treatment as id variables
```

## Re-Import count table annotations for all databases to create phyloseq object

```
#### Import count table
count <- read.csv("../input/contigAbundanceAll.tsv", sep = "\t",row.names = 1) 
colnames(count) <- gsub("^X","",colnames(count))
# remove samples with too few reads (sample 6: no reads mapped; sample 19: about 300 reads mapped) and reorder count data according to sample sheet
count <- count[,rownames(sample)]


# Import annotation tables and create count tables with multiple annotations duplicated 
#SEED
seed <- read.csv("../input/SEED.csv", sep = ",",row.names = 1)
#replace NA with "unclassified"
for (i in 2:6){
  seed[,i][is.na(seed[,i])] <- "unclassified"
}

head(seed)
```

```
# duplicate contigs with different level 4 annotations in count table to be able to aggregate them under multiple level 4 categories
# make annotation table with multiple annotations only when different at level 4 (drop multiple annotations at other levels)
seedLvl4Unique <- seed
# get combinations of contig and level 4
seedLvl4Unique[,"contigLvl4"] <- paste(seedLvl4Unique$contig,seedLvl4Unique$lvl4,sep = ";")
seedLvl4Unique <- seedLvl4Unique[!duplicated(seedLvl4Unique$contigLvl4),]

# remove unclassified
seedLvl4Unique <- seedLvl4Unique[seedLvl4Unique$lvl4!='unclassified',]

# count table with contigs annotated in seed
countS <- count[rownames(count)%in%seedLvl4Unique$contig,]

# duplicate contigs in count table if there are multiple annotations such that rownames of count table match annotation table

#remove suffix .1 for multiple annotations to make name rownames equal to those in count table 
rownames(seedLvl4Unique) <- gsub("\\.1$","",rownames(seedLvl4Unique))

for (i in rownames(seedLvl4Unique)[!(rownames(seedLvl4Unique)%in%rownames(countS))]){
  countS[i,1:26] <- countS[rownames(countS)==seedLvl4Unique[i,"contig"],1:26]
}

any(is.na(rownames(countS)))
```

```
## [1] FALSE
```

```
any(is.na(rownames(seedLvl4Unique)))
```

```
## [1] FALSE
```

```
all(rownames(countS)%in%rownames(seedLvl4Unique))
```

```
## [1] TRUE
```

```
all(rownames(seedLvl4Unique)%in%rownames(countS))
```

```
## [1] TRUE
```

```
# aggregate at lvl4
countLvl4 <- AbuTableInteger(countab = countS, taxo = seedLvl4Unique, col2matchcount = rownames(countS),
                             col2matchtax = rownames(seedLvl4Unique), Taxlevel = "lvl4", Samp = sample, fac = "sampleID")

#remove sample data
countLvl4 <- countLvl4[,-(1358:1382)]

#transpose
countLvl4 <- as.data.frame(t(countLvl4))

# double check aggregation
all(colSums(countLvl4) == colSums(countS))
```

```
## [1] TRUE
```

```
# rename rows
rownames(countLvl4) <- paste0('lvl4_',1:nrow(countLvl4))
phycount <- otu_table(countLvl4,taxa_are_rows = T)

#merge (leave out annotation table <=> not needed for further analyses)
phySEED <- merge_phyloseq(phycount,phySample)
rm(countS,countLvl4,phycount,seed,seedLvl4Unique)


#CAZy
cazy <- read.csv("../input/CAZY.csv", sep = ",",row.names = 1)
for (i in 2:6){
  cazy[,i][is.na(cazy[,i])] <- "unclassified"
}


#subset count table and annotation table to contigs annotated with Cazy
cazy <- cazy[cazy$annotation!="unclassified",]
countC <- count[rownames(cazy),]

all(colnames(countC)==sample$sampleID)
```

```
## [1] TRUE
```

```
any(is.na(rownames(countC)))
```

```
## [1] FALSE
```

```
any(is.na(rownames(cazy)))
```

```
## [1] FALSE
```

```
all(rownames(countC)%in%rownames(cazy))
```

```
## [1] TRUE
```

```
all(rownames(cazy)%in%rownames(countC))
```

```
## [1] TRUE
```

```
# aggregate at lvl2 (gene)
countFam <- AbuTableInteger(countab = countC, taxo = cazy, col2matchcount = rownames(countC),
                            col2matchtax = rownames(cazy), Taxlevel = "Family", Samp = sample, fac = "sampleID")

#remove sample data
countFam <- countFam[,-(314:338)]

#transpose
countFam <- as.data.frame(t(countFam))

# double check aggregation
all(colSums(countFam) == colSums(countC))
```

```
## [1] TRUE
```

```
phycount <- otu_table(countFam,taxa_are_rows = T)

phyCAZy <- merge_phyloseq(phycount,phySample)
rm(countC,phycount,cazy,countFam)


#Ncyc
Ncyc <- read.csv("../input/NcycFinal.csv", sep = ",",row.names = 1)

#replace NA with "unclassified"
for (i in 2:7){
  Ncyc[,i][is.na(Ncyc[,i])] <- "unclassified"
}
# remove spaces in beginning of lvl1and lvl2
Ncyc$lvl1 <- gsub("^ ","",Ncyc$lvl1)
Ncyc$lvl2 <- gsub("^ ","",Ncyc$lvl2)

# remove unclassified
Ncyc <- Ncyc[Ncyc$lvl2!='unclassified',]

# count table with contigs annotated in Ncyc
countN <- count[rownames(count)%in%Ncyc$contig,]


# duplicate contigs in count table if there are multiple annotations such that rownames of count table match annotation table

for (i in rownames(Ncyc)[!(rownames(Ncyc)%in%rownames(countN))]){
  countN[i,1:26] <- countN[rownames(countN)==Ncyc[i,"contig"],1:26]
}


all(colnames(countN)==sample$sampleID)
```

```
## [1] TRUE
```

```
any(is.na(rownames(countN)))
```

```
## [1] FALSE
```

```
any(is.na(rownames(Ncyc)))
```

```
## [1] FALSE
```

```
all(rownames(countN)%in%rownames(Ncyc))
```

```
## [1] TRUE
```

```
all(rownames(Ncyc)%in%rownames(countN))
```

```
## [1] TRUE
```

```
# aggregate at lvl2 (gene)
countLvl2 <- AbuTableInteger(countab = countN, taxo = Ncyc, col2matchcount = rownames(countN),
                             col2matchtax = rownames(Ncyc), Taxlevel = "lvl2", Samp = sample, fac = "sampleID")

#remove sample data
countLvl2 <- countLvl2[,-(48:72)]

#transpose
countLvl2 <- as.data.frame(t(countLvl2))

all(colSums(countLvl2) == colSums(countN))
```

```
## [1] TRUE
```

```
phycount <- otu_table(countLvl2,taxa_are_rows = T)

#merge 
phyNcyc <- merge_phyloseq(phycount,phySample)
rm(countN,phycount,Ncyc,countLvl2)
```

## Alphadiversity

```
# load agricolae package (not loaded in beginning because it interfers with DESeq2)
library(agricolae)
sessionInfo()
```

```
## R version 4.3.3 (2024-02-29 ucrt)
## Platform: x86_64-w64-mingw32/x64 (64-bit)
## Running under: Windows 11 x64 (build 26100)
## 
## Matrix products: default
## 
## 
## locale:
## [1] LC_COLLATE=English_United States.utf8 
## [2] LC_CTYPE=English_United States.utf8   
## [3] LC_MONETARY=English_United States.utf8
## [4] LC_NUMERIC=C                          
## [5] LC_TIME=English_United States.utf8    
## 
## time zone: Europe/Copenhagen
## tzcode source: internal
## 
## attached base packages:
## [1] grid      stats4    stats     graphics  grDevices utils     datasets 
## [8] methods   base     
## 
## other attached packages:
##  [1] agricolae_1.3-7             apeglm_1.24.0              
##  [3] ggnewscale_0.4.10           RColorBrewer_1.1-3         
##  [5] egg_0.4.5                   gridExtra_2.3              
##  [7] reshape2_1.4.4              vegan_2.6-4                
##  [9] lattice_0.22-5              permute_0.9-7              
## [11] lubridate_1.9.3             forcats_1.0.0              
## [13] stringr_1.5.1               dplyr_1.1.4                
## [15] purrr_1.0.2                 readr_2.1.5                
## [17] tidyr_1.3.1                 tibble_3.2.1               
## [19] ggplot2_3.5.0               tidyverse_2.0.0            
## [21] DESeq2_1.42.1               SummarizedExperiment_1.32.0
## [23] Biobase_2.62.0              MatrixGenerics_1.14.0      
## [25] matrixStats_1.2.0           GenomicRanges_1.54.1       
## [27] GenomeInfoDb_1.38.8         IRanges_2.36.0             
## [29] S4Vectors_0.40.2            BiocGenerics_0.48.1        
## [31] phyloseq_1.46.0            
## 
## loaded via a namespace (and not attached):
##  [1] bitops_1.0-7            rlang_1.1.3             magrittr_2.0.3         
##  [4] ade4_1.7-23             compiler_4.3.3          mgcv_1.9-1             
##  [7] vctrs_0.6.5             pkgconfig_2.0.3         crayon_1.5.3           
## [10] fastmap_1.2.0           XVector_0.42.0          labeling_0.4.3         
## [13] rmarkdown_2.29          tzdb_0.4.0              xfun_0.52              
## [16] zlibbioc_1.48.2         cachem_1.1.0            jsonlite_1.8.8         
## [19] biomformat_1.30.0       rhdf5filters_1.14.1     DelayedArray_0.28.0    
## [22] Rhdf5lib_1.24.2         BiocParallel_1.36.0     parallel_4.3.3         
## [25] cluster_2.1.8.1         R6_2.6.1                bslib_0.9.0            
## [28] stringi_1.8.3           jquerylib_0.1.4         numDeriv_2016.8-1.1    
## [31] Rcpp_1.0.12             iterators_1.0.14        knitr_1.50             
## [34] Matrix_1.6-5            splines_4.3.3           igraph_2.1.4           
## [37] timechange_0.3.0        tidyselect_1.2.1        rstudioapi_0.17.1      
## [40] abind_1.4-8             yaml_2.3.8              AlgDesign_1.2.1.2      
## [43] codetools_0.2-20        plyr_1.8.9              withr_3.0.2            
## [46] coda_0.19-4.1           evaluate_1.0.3          survival_3.5-8         
## [49] Biostrings_2.70.3       pillar_1.10.2           foreach_1.5.2          
## [52] generics_0.1.4          RCurl_1.98-1.14         emdbook_1.3.13         
## [55] hms_1.1.3               scales_1.4.0            glue_1.7.0             
## [58] tools_4.3.3             data.table_1.15.4       locfit_1.5-9.10        
## [61] mvtnorm_1.2-4           rhdf5_2.46.1            ape_5.8-1              
## [64] bbmle_1.0.25.1          bdsmatrix_1.3-7         colorspace_2.1-0       
## [67] nlme_3.1-164            GenomeInfoDbData_1.2.11 cli_3.6.2              
## [70] S4Arrays_1.2.1          gtable_0.3.6            sass_0.4.10            
## [73] digest_0.6.35           SparseArray_1.2.4       farver_2.1.1           
## [76] htmltools_0.5.8.1       multtest_2.58.0         lifecycle_1.0.4        
## [79] MASS_7.3-60.0.1
```

```
# seed
#rarefy
phyRar=rarefy_even_depth(phySEED,rngseed=T)
```

```
## `set.seed(TRUE)` was used to initialize repeatable random subsampling.
```

```
## Please record this for your records so others can reproduce.
```

```
## Try `set.seed(TRUE); .Random.seed` for the full vector
```

```
## ...
```

```
## 127OTUs were removed because they are no longer 
## present in any sample after random subsampling
```

```
## ...
```

```
# calculate indices
AlphadivS=estimate_richness(phyRar)
rm(phyRar)
# add sample data
all(rownames(AlphadivS)==rownames(sample))
```

```
## [1] FALSE
```

```
# remove X from rownames in Alphadiv
rownames(AlphadivS) <- gsub('^X','',rownames(AlphadivS))
all(rownames(AlphadivS)==rownames(sample))
```

```
## [1] TRUE
```

```
AlphadivS <- cbind(AlphadivS,sample)

# cazy
#rarefy
phyRar=rarefy_even_depth(phyCAZy,rngseed=T)
```

```
## `set.seed(TRUE)` was used to initialize repeatable random subsampling.
```

```
## Please record this for your records so others can reproduce.
```

```
## Try `set.seed(TRUE); .Random.seed` for the full vector
```

```
## ...
```

```
## 19OTUs were removed because they are no longer 
## present in any sample after random subsampling
```

```
## ...
```

```
# calculate indices
AlphadivC=estimate_richness(phyRar)
rm(phyRar)
# add sample data
all(rownames(AlphadivC)==rownames(sample))
```

```
## [1] FALSE
```

```
# remove X from rownames in Alphadiv
rownames(AlphadivC) <- gsub('^X','',rownames(AlphadivC))
all(rownames(AlphadivC)==rownames(sample))
```

```
## [1] TRUE
```

```
AlphadivC <- cbind(AlphadivC,sample)

# ncyc
#rarefy
phyRar=rarefy_even_depth(phyNcyc,rngseed=T)
```

```
## `set.seed(TRUE)` was used to initialize repeatable random subsampling.
```

```
## Please record this for your records so others can reproduce.
```

```
## Try `set.seed(TRUE); .Random.seed` for the full vector
```

```
## ...
```

```
## 2OTUs were removed because they are no longer 
## present in any sample after random subsampling
```

```
## ...
```

```
# calculate indices
AlphadivN=estimate_richness(phyRar)
rm(phyRar)
# add sample data
all(rownames(AlphadivN)==rownames(sample))
```

```
## [1] FALSE
```

```
# remove X from rownames in Alphadiv
rownames(AlphadivN) <- gsub('^X','',rownames(AlphadivN))
all(rownames(AlphadivN)==rownames(sample))
```

```
## [1] TRUE
```

```
AlphadivN <- cbind(AlphadivN,sample)

# Prokaryotes
#rarefy
phyRar=rarefy_even_depth(phyPro,rngseed=T)
```

```
## `set.seed(TRUE)` was used to initialize repeatable random subsampling.
```

```
## Please record this for your records so others can reproduce.
```

```
## Try `set.seed(TRUE); .Random.seed` for the full vector
```

```
## ...
```

```
## 2938OTUs were removed because they are no longer 
## present in any sample after random subsampling
```

```
## ...
```

```
# calculate indices
AlphadivP=estimate_richness(phyRar)
rm(phyRar)
# add sample data
all(rownames(AlphadivP)==rownames(sample))
```

```
## [1] FALSE
```

```
AlphadivP <- cbind(AlphadivP,sample)

# Eukaryotes
#rarefy
phyRar=rarefy_even_depth(phyEu,rngseed=T)
```

```
## `set.seed(TRUE)` was used to initialize repeatable random subsampling.
```

```
## Please record this for your records so others can reproduce.
```

```
## Try `set.seed(TRUE); .Random.seed` for the full vector
```

```
## ...
```

```
## 1607OTUs were removed because they are no longer 
## present in any sample after random subsampling
```

```
## ...
```

```
# calculate indices
AlphadivE=estimate_richness(phyRar)
rm(phyRar)
# add sample data
all(rownames(AlphadivE)==rownames(sample))
```

```
## [1] FALSE
```

```
AlphadivE <- cbind(AlphadivE,sample)
```

### Alphadiversity plots

```
### observed Richness
# seed
pS=ggplot(data = AlphadivS, aes(x=treatment, y=Observed))+stat_summary(fun.y="mean", geom="bar",position=position_dodge())+
  stat_summary(fun.y = mean,
               fun.ymin = function(x) mean(x) - sd(x), 
               fun.ymax = function(x) mean(x) + sd(x), 
               geom = "errorbar",width=0.2) +
  ylab("Richness")+xlab("") +ggtitle("seed")+
  theme(axis.text.x = element_text(angle = 60, hjust = 1)) +
  plot.theme1

pS=set_panel_size(pS,margin = unit(0, "mm"), width = unit(3, "inch"), height = unit(3, "inch"))


# cazy
pC=ggplot(data = AlphadivC, aes(x=treatment, y=Observed))+stat_summary(fun.y="mean", geom="bar",position=position_dodge())+
  stat_summary(fun.y = mean,
               fun.ymin = function(x) mean(x) - sd(x), 
               fun.ymax = function(x) mean(x) + sd(x), 
               geom = "errorbar",width=0.2) +
  ylab("Richness")+xlab("") +ggtitle("cazy")+
  theme(axis.text.x = element_text(angle = 60, hjust = 1)) +
  plot.theme1

pC=set_panel_size(pC,margin = unit(0, "mm"), width = unit(3, "inch"), height = unit(3, "inch"))

# ncyc
pN=ggplot(data = AlphadivN, aes(x=treatment, y=Observed))+stat_summary(fun.y="mean", geom="bar",position=position_dodge())+
  stat_summary(fun.y = mean,
               fun.ymin = function(x) mean(x) - sd(x), 
               fun.ymax = function(x) mean(x) + sd(x), 
               geom = "errorbar",width=0.2) +
  ylab("Richness")+xlab("") +ggtitle("ncycDB")+
  theme(axis.text.x = element_text(angle = 60, hjust = 1)) +
  plot.theme1

pN=set_panel_size(pN,margin = unit(0, "mm"), width = unit(3, "inch"), height = unit(3, "inch"))


grid.arrange(pS,pC,pN,ncol = 2)
```

```
# Prokaryotes
pP=ggplot(data = AlphadivP, aes(x=treatment, y=Observed))+stat_summary(fun.y="mean", geom="bar",position=position_dodge())+
  stat_summary(fun.y = mean,
               fun.ymin = function(x) mean(x) - sd(x), 
               fun.ymax = function(x) mean(x) + sd(x), 
               geom = "errorbar",width=0.2) +
  ylab("Richness")+xlab("") +ggtitle("Prokaryotes")+
  theme(axis.text.x = element_text(angle = 60, hjust = 1)) +
  plot.theme1

pP=set_panel_size(pP,margin = unit(0, "mm"), width = unit(3, "inch"), height = unit(3, "inch"))

# Eukaryotes
pE=ggplot(data = AlphadivE, aes(x=treatment, y=Observed))+stat_summary(fun.y="mean", geom="bar",position=position_dodge())+
  stat_summary(fun.y = mean,
               fun.ymin = function(x) mean(x) - sd(x), 
               fun.ymax = function(x) mean(x) + sd(x), 
               geom = "errorbar",width=0.2) +
  ylab("Richness")+xlab("") +ggtitle("Eukaryotes")+
  theme(axis.text.x = element_text(angle = 60, hjust = 1)) +
  plot.theme1

pE=set_panel_size(pE,margin = unit(0, "mm"), width = unit(3, "inch"), height = unit(3, "inch"))

grid.arrange(pP,pE,ncol = 2)
```

```
rm(pS,pC,p,pN,pP,pE)
```

### Stats

```
# seed
Alphadiv.stats=list()

for (i in c("Observed","Shannon")){
  Alphadiv.stats[[i]]=list()
  Alphadiv.stats[[i]][["aov"]]=aov(data=AlphadivS,get(i)~treatment)
  Alphadiv.stats[[i]][["shapiro"]]=shapiro.test(Alphadiv.stats[[i]][["aov"]]$residuals)
  Alphadiv.stats[[i]][["bartlett"]]=bartlett.test(data=AlphadivS,get(i)~treatment)
  Alphadiv.stats[[i]][["TukeyHSD"]]=TukeyHSD(Alphadiv.stats[[i]][["aov"]])
  Alphadiv.stats[[i]][["Tukeygroup"]]=HSD.test(Alphadiv.stats[[i]][["aov"]],"treatment",group=T)
  Alphadiv.stats[[i]][["KruskallWallis"]]=kruskal.test(data=AlphadivS,get(i)~treatment)
  
}

# diagnostic plots
par(mfrow = c(2,2))
plot(Alphadiv.stats$Observed$aov)
```

```
#summarize in dataframe results of shapiro (normality) and bartlett test (homoskedasticity)
stats.valid=data.frame()
for(i in c("Observed","Shannon")){
  stats.valid[i,"p_shapiro"]=Alphadiv.stats[[i]][["shapiro"]]$p.value
  stats.valid[i,"p_bartlett"]=Alphadiv.stats[[i]][["bartlett"]]$p.value
  
}

print(stats.valid)
```

```
##            p_shapiro  p_bartlett
## Observed 0.006799138 0.010753312
## Shannon  0.002062508 0.006544648
```

```
#print groups from Tukeytest with agricolae
for (i in c("Observed","Shannon")){print(i)
  print(Alphadiv.stats[[i]][["Tukeygroup"]]$groups)
}
```

```
## [1] "Observed"
##              get(i) groups
## drought T3 345.0000      a
## drought T5 341.7500      a
## drought T4 310.3333      a
## rewetting  309.2500      a
## drought T1 290.3333      a
## drought T2 290.2500      a
## control    285.7500      a
## [1] "Shannon"
##              get(i) groups
## drought T3 5.372101      a
## drought T4 5.297999      a
## drought T5 5.255871      a
## drought T2 5.195355      a
## drought T1 5.080000      a
## rewetting  5.068699      a
## control    5.049974      a
```

```
#treatment Effect Observed
anova(Alphadiv.stats$Observed$aov)
```

```
# Cazy
Alphadiv.stats=list()

for (i in c("Observed","Shannon")){
  Alphadiv.stats[[i]]=list()
  Alphadiv.stats[[i]][["aov"]]=aov(data=AlphadivC,get(i)~treatment)
  Alphadiv.stats[[i]][["shapiro"]]=shapiro.test(Alphadiv.stats[[i]][["aov"]]$residuals)
  Alphadiv.stats[[i]][["bartlett"]]=bartlett.test(data=AlphadivC,get(i)~treatment)
  Alphadiv.stats[[i]][["TukeyHSD"]]=TukeyHSD(Alphadiv.stats[[i]][["aov"]])
  Alphadiv.stats[[i]][["Tukeygroup"]]=HSD.test(Alphadiv.stats[[i]][["aov"]],"treatment",group=T)
  Alphadiv.stats[[i]][["KruskallWallis"]]=kruskal.test(data=AlphadivC,get(i)~treatment)
  
}

# diagnostic plots
par(mfrow = c(2,2))
plot(Alphadiv.stats$Observed$aov)
```

```
#summarize in dataframe results of shapiro (normality) and bartlett test (homoskedasticity)
stats.valid=data.frame()
for(i in c("Observed","Shannon")){
  stats.valid[i,"p_shapiro"]=Alphadiv.stats[[i]][["shapiro"]]$p.value
  stats.valid[i,"p_bartlett"]=Alphadiv.stats[[i]][["bartlett"]]$p.value
  
}

print(stats.valid)
```

```
##           p_shapiro  p_bartlett
## Observed 0.02484003 0.008350722
## Shannon  0.56116489 0.265972032
```

```
#print groups from Tukeytest with agricolae
for (i in c("Observed","Shannon")){print(i)
  print(Alphadiv.stats[[i]][["Tukeygroup"]]$groups)
}
```

```
## [1] "Observed"
##              get(i) groups
## drought T5 169.0000      a
## drought T3 166.2500      a
## rewetting  159.7500      a
## drought T4 156.6667      a
## control    155.5000      a
## drought T1 153.6667      a
## drought T2 150.7500      a
## [1] "Shannon"
##              get(i) groups
## drought T3 3.559400      a
## drought T4 3.557002     ab
## rewetting  3.460059     ab
## drought T5 3.406529     ab
## control    3.309577     ab
## drought T1 3.300199     ab
## drought T2 3.205442      b
```

```
#treatment Effect Observed
anova(Alphadiv.stats$Observed$aov)
```

```
#treatment Effect Shannon
anova(Alphadiv.stats$Shannon$aov)
```

```
# ncyc
Alphadiv.stats=list()

for (i in c("Observed","Shannon")){
  Alphadiv.stats[[i]]=list()
  Alphadiv.stats[[i]][["aov"]]=aov(data=AlphadivN,get(i)~treatment)
  Alphadiv.stats[[i]][["shapiro"]]=shapiro.test(Alphadiv.stats[[i]][["aov"]]$residuals)
  Alphadiv.stats[[i]][["bartlett"]]=bartlett.test(data=AlphadivN,get(i)~treatment)
  Alphadiv.stats[[i]][["TukeyHSD"]]=TukeyHSD(Alphadiv.stats[[i]][["aov"]])
  Alphadiv.stats[[i]][["Tukeygroup"]]=HSD.test(Alphadiv.stats[[i]][["aov"]],"treatment",group=T)
  Alphadiv.stats[[i]][["KruskallWallis"]]=kruskal.test(data=AlphadivN,get(i)~treatment)
  
}

# diagnostic plots
par(mfrow = c(2,2))
plot(Alphadiv.stats$Observed$aov)
```

```
#summarize in dataframe results of shapiro (normality) and bartlett test (homoskedasticity)
stats.valid=data.frame()
for(i in c("Observed","Shannon")){
  stats.valid[i,"p_shapiro"]=Alphadiv.stats[[i]][["shapiro"]]$p.value
  stats.valid[i,"p_bartlett"]=Alphadiv.stats[[i]][["bartlett"]]$p.value
  
}

print(stats.valid)
```

```
##          p_shapiro p_bartlett
## Observed 0.1740692  0.1608419
## Shannon  0.8020735  0.4361931
```

```
#print groups from Tukeytest with agricolae
for (i in c("Observed","Shannon")){print(i)
  print(Alphadiv.stats[[i]][["Tukeygroup"]]$groups)
}
```

```
## [1] "Observed"
##              get(i) groups
## rewetting  34.75000      a
## control    34.50000      a
## drought T5 34.25000      a
## drought T3 33.25000      a
## drought T1 32.66667      a
## drought T4 30.66667      a
## drought T2 30.50000      a
## [1] "Shannon"
##              get(i) groups
## drought T4 2.499367      a
## drought T5 2.481660      a
## drought T1 2.470917     ab
## drought T3 2.436865     ab
## control    2.431705     ab
## rewetting  2.382205      b
## drought T2 2.377753      b
```

```
#treatment Effect Observed
anova(Alphadiv.stats$Observed$aov)
```

```
par(mfrow = c(1,1))

# Prokaryotes
Alphadiv.stats=list()

for (i in c("Observed","Shannon","Chao1")){
  Alphadiv.stats[[i]]=list()
  Alphadiv.stats[[i]][["aov"]]=aov(data=AlphadivP,get(i)~treatment)
  Alphadiv.stats[[i]][["shapiro"]]=shapiro.test(Alphadiv.stats[[i]][["aov"]]$residuals)
  Alphadiv.stats[[i]][["bartlett"]]=bartlett.test(data=AlphadivP,get(i)~treatment)
  Alphadiv.stats[[i]][["TukeyHSD"]]=TukeyHSD(Alphadiv.stats[[i]][["aov"]])
  Alphadiv.stats[[i]][["Tukeygroup"]]=HSD.test(Alphadiv.stats[[i]][["aov"]],"treatment",group=T)
  Alphadiv.stats[[i]][["KruskallWallis"]]=kruskal.test(data=AlphadivP,get(i)~treatment)
  
}

# diagnostic plots
par(mfrow = c(2,2))
plot(Alphadiv.stats$Observed$aov)
```

```
plot(Alphadiv.stats$Shannon$aov)
```

```
#summarize in dataframe results of shapiro (normality) and bartlett test (homoskedasticity)
stats.valid=data.frame()
for(i in c("Observed","Shannon","Chao1")){
  stats.valid[i,"p_shapiro"]=Alphadiv.stats[[i]][["shapiro"]]$p.value
  stats.valid[i,"p_bartlett"]=Alphadiv.stats[[i]][["bartlett"]]$p.value
  
}

print(stats.valid)
```

```
##          p_shapiro p_bartlett
## Observed 0.9397911 0.43123798
## Shannon  0.2469146 0.48459477
## Chao1    0.9552379 0.08047678
```

```
#print groups from Tukeytest with agricolae
for (i in c("Observed","Shannon","Chao1")){print(i)
  print(Alphadiv.stats[[i]][["Tukeygroup"]]$groups)
}
```

```
## [1] "Observed"
##              get(i) groups
## control    3353.500      a
## drought T5 3337.750     ab
## rewetting  3288.000     ab
## drought T1 3278.333     ab
## drought T2 3217.250     ab
## drought T4 3167.667     ab
## drought T3 3072.500      b
## [1] "Shannon"
##              get(i) groups
## drought T4 4.734064      a
## rewetting  4.712087      a
## drought T5 4.707597      a
## drought T3 4.676942     ab
## drought T2 4.629812      b
## control    4.622591      b
## drought T1 4.602851      b
## [1] "Chao1"
##              get(i) groups
## rewetting  4394.416      a
## control    4377.589      a
## drought T5 4295.561     ab
## drought T1 4226.249     ab
## drought T2 3990.565     ab
## drought T4 3756.559     ab
## drought T3 3715.951      b
```

```
#treatment Effect Observed
anova(Alphadiv.stats$Observed$aov)
```

```
# Eukaryotes
Alphadiv.stats=list()

for (i in c("Observed","Shannon","Chao1")){
  Alphadiv.stats[[i]]=list()
  Alphadiv.stats[[i]][["aov"]]=aov(data=AlphadivE,get(i)~treatment)
  Alphadiv.stats[[i]][["shapiro"]]=shapiro.test(Alphadiv.stats[[i]][["aov"]]$residuals)
  Alphadiv.stats[[i]][["bartlett"]]=bartlett.test(data=AlphadivE,get(i)~treatment)
  Alphadiv.stats[[i]][["TukeyHSD"]]=TukeyHSD(Alphadiv.stats[[i]][["aov"]])
  Alphadiv.stats[[i]][["Tukeygroup"]]=HSD.test(Alphadiv.stats[[i]][["aov"]],"treatment",group=T)
  Alphadiv.stats[[i]][["KruskallWallis"]]=kruskal.test(data=AlphadivE,get(i)~treatment)
  
}

# diagnostic plots
par(mfrow = c(2,2))
plot(Alphadiv.stats$Observed$aov)
```

```
plot(Alphadiv.stats$Shannon$aov)
```

```
#summarize in dataframe results of shapiro (normality) and bartlett test (homoskedasticity)
stats.valid=data.frame()
for(i in c("Observed","Shannon","Chao1")){
  stats.valid[i,"p_shapiro"]=Alphadiv.stats[[i]][["shapiro"]]$p.value
  stats.valid[i,"p_bartlett"]=Alphadiv.stats[[i]][["bartlett"]]$p.value
  
}

print(stats.valid)
```

```
##           p_shapiro p_bartlett
## Observed 0.56547164 0.12474853
## Shannon  0.04250593 0.78220052
## Chao1    0.56406834 0.09743675
```

```
#print groups from Tukeytest with agricolae
for (i in c("Observed","Shannon","Chao1")){print(i)
  print(Alphadiv.stats[[i]][["Tukeygroup"]]$groups)
}
```

```
## [1] "Observed"
##              get(i) groups
## control    831.7500      a
## drought T1 789.6667     ab
## drought T2 782.0000     ab
## drought T5 761.2500     ab
## drought T3 743.0000     ab
## rewetting  740.5000     ab
## drought T4 720.6667      b
## [1] "Shannon"
##              get(i) groups
## drought T3 3.926833      a
## drought T1 3.922980      a
## drought T2 3.843564      a
## control    3.827402     ab
## drought T5 3.807400     ab
## drought T4 3.797063     ab
## rewetting  3.704948      b
## [1] "Chao1"
##               get(i) groups
## control    1218.4376      a
## drought T1 1111.9905     ab
## drought T5 1099.7498     ab
## drought T2 1082.6754     ab
## rewetting  1032.4597     ab
## drought T3  956.2633      b
## drought T4  911.1265      b
```

```
#treatment Effect Observed
anova(Alphadiv.stats$Observed$aov)
```

## Functional gene and taxonomic community structures

### PERMANOVA

#### With relative abundances

- seed (code shown for seed, same code used for other databases

```
phyRel <- transform_sample_counts(phySEED,function(x)x/sum(x))

#extract table of rel. abundances from physeq
SRel <- as.data.frame(as.matrix(phyRel@otu_table@.Data))
SRel <- as.data.frame(t(SRel))

all(rownames(SRel)==rownames(sample))
```

```
## [1] TRUE
```

```
#permanova
adonis2(SRel~treatment,data=sample,method="bray",permutations = 9999)
```

```
#homogeneity of variance
betad <- betadisper(vegdist(SRel,method="bray"),sample$treatment)
permutest(betad,permutations = 9999)
```

```
## 
## Permutation test for homogeneity of multivariate dispersions
## Permutation: free
## Number of permutations: 9999
## 
## Response: Distances
##           Df   Sum Sq   Mean Sq      F N.Perm Pr(>F)  
## Groups     6 0.026836 0.0044727 2.1155   9999 0.0779 .
## Residuals 19 0.040171 0.0021143                       
## ---
## Signif. codes:  0 '***' 0.001 '**' 0.01 '*' 0.05 '.' 0.1 ' ' 1
```

- cazy

```
## [1] TRUE
```

```
## 
## Permutation test for homogeneity of multivariate dispersions
## Permutation: free
## Number of permutations: 9999
## 
## Response: Distances
##           Df    Sum Sq    Mean Sq      F N.Perm Pr(>F)
## Groups     6 0.0034057 0.00056761 0.5491   9999 0.7584
## Residuals 19 0.0196390 0.00103363
```

- Ncyc

```
## [1] TRUE
```

```
## 
## Permutation test for homogeneity of multivariate dispersions
## Permutation: free
## Number of permutations: 9999
## 
## Response: Distances
##           Df   Sum Sq    Mean Sq     F N.Perm Pr(>F)
## Groups     6 0.003679 0.00061317 0.538   9999 0.7752
## Residuals 19 0.021656 0.00113979
```

- rrna genes, Prokaryotes

```
## [1] TRUE
```

```
## 
## Permutation test for homogeneity of multivariate dispersions
## Permutation: free
## Number of permutations: 9999
## 
## Response: Distances
##           Df    Sum Sq    Mean Sq      F N.Perm Pr(>F)
## Groups     6 0.0006590 0.00010984 0.2324   9999 0.9621
## Residuals 19 0.0089786 0.00047256
```

- rrna genes, Prokaryotes

```
## [1] TRUE
```

```
## 
## Permutation test for homogeneity of multivariate dispersions
## Permutation: free
## Number of permutations: 9999
## 
## Response: Distances
##           Df    Sum Sq    Mean Sq      F N.Perm Pr(>F)
## Groups     6 0.0017605 0.00029342 0.2185   9999 0.9662
## Residuals 19 0.0255133 0.00134281
```

#### Normalized by RNA content weighted by mrna fraction

Normalization by multiplying relative abundances with RNA content,
weighted by the fraction of mRNA in each sample \* SEED (same code used
for other databases)

```
#SEED
phyRel <- transform_sample_counts(phySEED,function(x)x/sum(x))
# normalize by RNA content 
all(colnames(phyRel@otu_table)==rownames(phyRel@sam_data))
```

```
## [1] TRUE
```

```
phyRel@otu_table <- otu_table(t(apply(phyRel@otu_table,1,function(x)x*phyRel@sam_data$RNA_DW_mrna_sc)), taxa_are_rows = T)

#extract table
SRel <- as.data.frame(as.matrix(phyRel@otu_table@.Data))
SRel <- as.data.frame(t(SRel))

all(rownames(SRel)==rownames(sample))
```

```
## [1] TRUE
```

```
#permanova
adonis2(SRel~treatment,data=sample,method="bray",permutations = 9999)
```

```
#homogeneity of variance
betad <- betadisper(vegdist(SRel,method="bray"),sample$treatment)
permutest(betad,permutations = 9999)
```

```
## 
## Permutation test for homogeneity of multivariate dispersions
## Permutation: free
## Number of permutations: 9999
## 
## Response: Distances
##           Df   Sum Sq   Mean Sq      F N.Perm Pr(>F)
## Groups     6 0.028352 0.0047253 0.9579   9999  0.484
## Residuals 19 0.093721 0.0049327
```

```
## [1] TRUE
```

```
## [1] TRUE
```

```
## 
## Permutation test for homogeneity of multivariate dispersions
## Permutation: free
## Number of permutations: 9999
## 
## Response: Distances
##           Df   Sum Sq   Mean Sq      F N.Perm Pr(>F)
## Groups     6 0.023419 0.0039031 0.4356   9999 0.8456
## Residuals 19 0.170247 0.0089604
```

- Ncyc

```
## [1] TRUE
```

```
## [1] TRUE
```

```
## 
## Permutation test for homogeneity of multivariate dispersions
## Permutation: free
## Number of permutations: 9999
## 
## Response: Distances
##           Df   Sum Sq   Mean Sq      F N.Perm Pr(>F)
## Groups     6 0.025478 0.0042464 0.4775   9999 0.8142
## Residuals 19 0.168978 0.0088936
```

### PcoA with environmental variables (envfit)

#### Relative abundances

- For SEED

```
# define colors for plots
col1 <- brewer.pal(9,"Oranges")
col1 <- col1[5:9]
col1 <- c("grey50",col1,"cornflowerblue")
names(col1) <- unique(sample$treatment)

#SEED
phyRel <- transform_sample_counts(phySEED,function(x)x/sum(x))

#extract count table
SRel <- as.data.frame(as.matrix(phyRel@otu_table@.Data))
SRel <- as.data.frame(t(SRel))

# PCoA with vegan
cmd <- cmdscale(vegdist(SRel,method="bray"),eig=TRUE) #PCoA with vegan

# Calculate percentage explained by axis
# Axis 1 for all databases
xvar <- paste0("Axis 1 [",round((cmd$eig[1]/sum(cmd$eig))*100,digits=1),"%]")

#A xis 2 for all databases
yvar <- paste0("Axis 2 [",round((cmd$eig[2]/sum(cmd$eig))*100,digits=1),"%]")

# define tips of arrows
arrowhead <- arrow(length = unit(0.02, "npc"))

# create data frames for input data and add sample information
datCmd <- as.data.frame(cmd$points)
all(rownames(datCmd)==rownames(sample))
```

```
## [1] TRUE
```

```
datCmd <- cbind(datCmd,sample)

# envfit: correlate env variables with axes of pcoa
all(rownames(sample)==rownames(SRel))
```

```
## [1] TRUE
```

```
fit1 <- envfit(cmd, sample[,3:14], permutations=9999, na.rm=T)
print(fit1$vectors)
```

```
##               Dim1     Dim2     r2 Pr(>r)    
## WC        -0.75894 -0.65116 0.8668 0.0001 ***
## DOC       -0.64933  0.76051 0.1147 0.2445    
## DON       -0.87829  0.47813 0.0788 0.3961    
## NH4       -0.36604 -0.93060 0.1736 0.1078    
## NO3        0.89764  0.44072 0.1218 0.2273    
## PO4       -0.98055  0.19625 0.2587 0.0178 *  
## RNA       -0.99893  0.04631 0.2374 0.0349 *  
## Laccase   -0.99831 -0.05807 0.5479 0.0002 ***
## Cellulase -0.98686 -0.16158 0.4076 0.0017 ** 
## CO2       -0.94169 -0.33649 0.6521 0.0001 ***
## CH4        0.11461 -0.99341 0.1029 0.2833    
## N2O       -0.26360 -0.96463 0.0913 0.3407    
## ---
## Signif. codes:  0 '***' 0.001 '**' 0.01 '*' 0.05 '.' 0.1 ' ' 1
## Permutation: free
## Number of permutations: 9999
```

```
#extract arrows
arrowDat <- scores(fit1,display = "vectors")

#plot
pS <- ggplot(data=datCmd,aes(x=V1,y=V2,color=treatment))+geom_point(size=4)+
  geom_segment(aes(xend=0.3*Dim1,yend=0.3*Dim2,x=0,y=0,shape=NULL,color=NULL), 
               size = 1, 
               data = as.data.frame(arrowDat), 
               color = "gray", 
               arrow=arrow(length = unit(0.02, "npc"))) + 
  geom_text(aes(x = 0.35* Dim1, 
                y = 0.35* Dim2, 
                shape = NULL, 
                color = NULL), 
            size = 4, 
            color = "gray", 
            data = as.data.frame(arrowDat), 
            show_guide = FALSE,label=rownames(arrowDat))+
  plot.theme1+
  scale_color_manual(values=col1)+
  labs(color="Treatment")+ggtitle('seed')+
  xlab(xvar)+
  ylab(yvar)


#fix panel size
pS <- set_panel_size(pS,margin = unit(0, "mm"), width = unit(4, "inch"), height = unit(4, "inch"))
```

- same for CAZy

```
## [1] TRUE
```

```
## [1] TRUE
```

```
##               Dim1     Dim2     r2 Pr(>r)    
## WC        -0.43867  0.89865 0.2467 0.0347 *  
## DOC       -0.23472  0.97206 0.1963 0.0849 .  
## DON       -0.15331  0.98818 0.2808 0.0282 *  
## NH4        0.02475  0.99969 0.3239 0.0133 *  
## NO3        0.25530  0.96686 0.3313 0.0088 ** 
## PO4       -0.93458  0.35575 0.2593 0.0463 *  
## RNA       -0.67204 -0.74051 0.2303 0.0587 .  
## Laccase   -0.64467  0.76446 0.5451 0.0001 ***
## Cellulase -0.98929  0.14595 0.1240 0.2168    
## CO2       -0.41299  0.91074 0.4056 0.0022 ** 
## CH4       -0.00982 -0.99995 0.2065 0.0668 .  
## N2O        0.92122  0.38905 0.0034 0.9432    
## ---
## Signif. codes:  0 '***' 0.001 '**' 0.01 '*' 0.05 '.' 0.1 ' ' 1
## Permutation: free
## Number of permutations: 9999
```

- Ncyc

```
## [1] TRUE
```

```
## [1] TRUE
```

```
##               Dim1     Dim2     r2 Pr(>r)    
## WC        -0.06364  0.99797 0.2948 0.0174 *  
## DOC       -0.34896  0.93714 0.0878 0.3528    
## DON       -0.15791  0.98745 0.0683 0.4537    
## NH4        0.63302  0.77414 0.1176 0.2360    
## NO3        0.82699 -0.56222 0.0845 0.3760    
## PO4       -0.46972  0.88282 0.3979 0.0008 ***
## RNA       -0.79381  0.60816 0.1384 0.1780    
## Laccase   -0.66503  0.74681 0.2669 0.0279 *  
## Cellulase -0.34395  0.93899 0.1361 0.1908    
## CO2       -0.23290  0.97250 0.2032 0.0729 .  
## CH4       -0.00712  0.99997 0.0375 0.6504    
## N2O        0.15992  0.98713 0.1495 0.1501    
## ---
## Signif. codes:  0 '***' 0.001 '**' 0.01 '*' 0.05 '.' 0.1 ' ' 1
## Permutation: free
## Number of permutations: 9999
```

Plot PCoAs for all databases

```
grid.arrange(pS ,pC,pN,ncol=2)
```

\* for rrna genes (prokaryotes and eukaryotes; same analyses as for
functional genes) \*prokaryotes

```
## [1] TRUE
```

```
## [1] TRUE
```

```
##               Dim1     Dim2     r2 Pr(>r)    
## WC        -0.13577  0.99074 0.7901 0.0001 ***
## DOC       -0.88105  0.47303 0.3830 0.0039 ** 
## DON       -0.69748  0.71660 0.2970 0.0157 *  
## NH4        0.17174  0.98514 0.3804 0.0033 ** 
## NO3       -0.41302  0.91072 0.0078 0.9162    
## PO4       -0.78132  0.62413 0.2368 0.0432 *  
## RNA       -0.93542 -0.35355 0.0518 0.5601    
## Laccase   -0.55970  0.82869 0.5881 0.0002 ***
## Cellulase -0.68765  0.72604 0.4683 0.0009 ***
## CO2       -0.33138  0.94350 0.8378 0.0001 ***
## CH4        0.81154 -0.58430 0.2075 0.0709 .  
## N2O        0.51457  0.85745 0.1763 0.1434    
## ---
## Signif. codes:  0 '***' 0.001 '**' 0.01 '*' 0.05 '.' 0.1 ' ' 1
## Permutation: free
## Number of permutations: 9999
```

- for eukaryotes

```
## [1] TRUE
```

```
## [1] TRUE
```

```
##               Dim1     Dim2     r2 Pr(>r)   
## WC         0.30015 -0.95389 0.3543 0.0083 **
## DOC        0.91624 -0.40062 0.0811 0.3713   
## DON        0.71823 -0.69581 0.0572 0.5145   
## NH4       -0.52556 -0.85076 0.2847 0.0195 * 
## NO3       -0.99974 -0.02293 0.0246 0.7497   
## PO4        0.88904 -0.45783 0.2038 0.0559 . 
## RNA        0.91282 -0.40835 0.0419 0.6354   
## Laccase    0.54064 -0.84125 0.3151 0.0124 * 
## Cellulase  0.95568 -0.29440 0.1635 0.1320   
## CO2        0.43789 -0.89903 0.2603 0.0309 * 
## CH4        0.48179  0.87628 0.1532 0.1488   
## N2O       -0.39800 -0.91739 0.1329 0.1757   
## ---
## Signif. codes:  0 '***' 0.001 '**' 0.01 '*' 0.05 '.' 0.1 ' ' 1
## Permutation: free
## Number of permutations: 9999
```

#### Normalized by RNA content weighted mrna fraction

Normalization as for PERMANOVA, otherwise same analysis as for
relative abundances \* SEED

```
## [1] TRUE
```

```
## [1] TRUE
```

```
## [1] TRUE
```

```
##               Dim1     Dim2     r2 Pr(>r)   
## WC        -0.99146  0.13039 0.3004 0.0150 * 
## DOC       -0.73615  0.67682 0.0011 0.9858   
## DON       -0.99937  0.03560 0.0063 0.9191   
## NH4       -0.95357  0.30118 0.0026 0.9720   
## NO3        0.99987 -0.01637 0.2451 0.0384 * 
## PO4       -0.76213  0.64742 0.1986 0.0909 . 
## Laccase   -0.54872  0.83601 0.3774 0.0034 **
## Cellulase -0.69894  0.71518 0.4429 0.0014 **
## CO2       -0.74428  0.66787 0.2624 0.0278 * 
## CH4       -0.04631 -0.99893 0.1526 0.1453   
## N2O       -0.38945 -0.92105 0.0418 0.4758   
## ---
## Signif. codes:  0 '***' 0.001 '**' 0.01 '*' 0.05 '.' 0.1 ' ' 1
## Permutation: free
## Number of permutations: 9999
```

- CAZy

```
## [1] TRUE
```

```
## [1] TRUE
```

```
## [1] TRUE
```

```
##               Dim1     Dim2     r2 Pr(>r)   
## WC        -0.98723  0.15931 0.2565 0.0320 * 
## DOC       -0.28703 -0.95792 0.0048 0.9453   
## DON       -0.55317 -0.83307 0.0136 0.8375   
## NH4       -0.99959 -0.02866 0.0023 0.9718   
## NO3        0.97708 -0.21289 0.2219 0.0565 . 
## PO4       -0.79393  0.60801 0.1689 0.1253   
## Laccase   -0.54470  0.83863 0.3250 0.0098 **
## Cellulase -0.65982  0.75142 0.4210 0.0022 **
## CO2       -0.72923  0.68427 0.2181 0.0549 . 
## CH4       -0.04720 -0.99889 0.1277 0.2028   
## N2O       -0.35663 -0.93424 0.0381 0.5132   
## ---
## Signif. codes:  0 '***' 0.001 '**' 0.01 '*' 0.05 '.' 0.1 ' ' 1
## Permutation: free
## Number of permutations: 9999
```

- Ncyc

```
## [1] TRUE
```

```
## [1] TRUE
```

```
## [1] TRUE
```

```
##               Dim1     Dim2     r2 Pr(>r)   
## WC        -0.97253  0.23278 0.2582 0.0285 * 
## DOC       -0.38173 -0.92427 0.0036 0.9580   
## DON       -0.58589 -0.81039 0.0140 0.8363   
## NH4       -0.74648 -0.66541 0.0035 0.9579   
## NO3        0.94204 -0.33550 0.2209 0.0560 . 
## PO4       -0.76086  0.64892 0.1717 0.1216   
## Laccase   -0.52018  0.85406 0.3361 0.0075 **
## Cellulase -0.64209  0.76663 0.4271 0.0025 **
## CO2       -0.70025  0.71390 0.2247 0.0486 * 
## CH4       -0.04439 -0.99901 0.1131 0.2550   
## N2O       -0.40712 -0.91338 0.0325 0.5791   
## ---
## Signif. codes:  0 '***' 0.001 '**' 0.01 '*' 0.05 '.' 0.1 ' ' 1
## Permutation: free
## Number of permutations: 9999
```

Plots for all databases

## microbial activity and soil chemistry

### Plot sample data

```
# convert negative values for enzymes to zero
sample$Laccase <- ifelse(sample$Laccase<0,0,sample$Laccase)
sample$Cellulase <- ifelse(sample$Cellulase<0,0,sample$Cellulase)

# calculate respiration to DNA ratio
sample$resp_RNA <- sample$CO2/sample$RNA_DW

# DOC/DON ratio
sample$DOC_DON_rat <- sample$DOC/sample$DON

# DOC/DON+DIN ratio
sample$DOC_DON_DIN_rat <- sample$DOC/(sample$DON+sample$NH4+sample$NO3)
```

```
sampleL <- melt(sample)
```

```
## Using sampleID, treatment as id variables
```

```
ggplot(data = sampleL, aes(x=treatment, y=value))+stat_summary(fun.y="mean", geom="bar",position=position_dodge())+
  stat_summary(fun.y = mean,
               fun.ymin = function(x) mean(x) - sd(x), 
               fun.ymax = function(x) mean(x) + sd(x), 
               geom = "errorbar",width=0.2) +
  facet_wrap(~variable, scales = "free")+
  ylab("")+xlab("")+
  theme(axis.text.x = element_text(angle = 60, hjust = 1)) +
  plot.theme1
```

### statistical analyses

```
psc <- 0.00000000001 # pseudocount for log transformation of data with 0
# function to calculate cube roots (for transformation of negative vlaues)
cbrt<-function(x){
  sign(x)*abs(x)^(1/3)
}


par(mfrow = c(3,4))
mod1 <- lm(WC ~ treatment , data = sample) 
any(sample$WC==0)
```

```
## [1] FALSE
```

```
mod1l <- lm(log(WC) ~ treatment, data = sample) 
mod1sq <- lm(sqrt(WC) ~ treatment, data = sample) 
plot(mod1)# best
plot(mod1l)
plot(mod1sq)
```

```
summary(mod1)
```

```
## 
## Call:
## lm(formula = WC ~ treatment, data = sample)
## 
## Residuals:
##     Min      1Q  Median      3Q     Max 
## -5.3500 -0.4263  0.0000  0.8356  4.8900 
## 
## Coefficients:
##                     Estimate Std. Error  t value Pr(>|t|)    
## (Intercept)          179.522      1.165  154.142  < 2e-16 ***
## treatmentdrought T1  -15.992      1.779   -8.989 2.84e-08 ***
## treatmentdrought T2  -58.392      1.647  -35.452  < 2e-16 ***
## treatmentdrought T3  -97.687      1.647  -59.310  < 2e-16 ***
## treatmentdrought T4 -130.392      1.779  -73.294  < 2e-16 ***
## treatmentdrought T5 -165.122      1.647 -100.252  < 2e-16 ***
## treatmentrewetting     1.368      1.647    0.830    0.417    
## ---
## Signif. codes:  0 '***' 0.001 '**' 0.01 '*' 0.05 '.' 0.1 ' ' 1
## 
## Residual standard error: 2.329 on 19 degrees of freedom
## Multiple R-squared:  0.999,  Adjusted R-squared:  0.9986 
## F-statistic:  3043 on 6 and 19 DF,  p-value: < 2.2e-16
```

```
summary(mod1l)
```

```
## 
## Call:
## lm(formula = log(WC) ~ treatment, data = sample)
## 
## Residuals:
##      Min       1Q   Median       3Q      Max 
## -0.36712 -0.00528  0.00001  0.00897  0.19994 
## 
## Coefficients:
##                      Estimate Std. Error t value Pr(>|t|)    
## (Intercept)          5.190294   0.051539 100.705  < 2e-16 ***
## treatmentdrought T1 -0.093301   0.078728  -1.185    0.251    
## treatmentdrought T2 -0.393895   0.072888  -5.404 3.25e-05 ***
## treatmentdrought T3 -0.785799   0.072888 -10.781 1.55e-09 ***
## treatmentdrought T4 -1.295864   0.078728 -16.460 1.06e-12 ***
## treatmentdrought T5 -2.545911   0.072888 -34.929  < 2e-16 ***
## treatmentrewetting   0.007589   0.072888   0.104    0.918    
## ---
## Signif. codes:  0 '***' 0.001 '**' 0.01 '*' 0.05 '.' 0.1 ' ' 1
## 
## Residual standard error: 0.1031 on 19 degrees of freedom
## Multiple R-squared:  0.9901, Adjusted R-squared:  0.9869 
## F-statistic: 315.9 on 6 and 19 DF,  p-value: < 2.2e-16
```

```
summary(mod1sq)
```

```
## 
## Call:
## lm(formula = sqrt(WC) ~ treatment, data = sample)
## 
## Residuals:
##      Min       1Q   Median       3Q      Max 
## -0.65142 -0.02447  0.00002  0.03437  0.37216 
## 
## Coefficients:
##                     Estimate Std. Error t value Pr(>|t|)    
## (Intercept)         13.39858    0.10167 131.784  < 2e-16 ***
## treatmentdrought T1 -0.61070    0.15530  -3.932 0.000894 ***
## treatmentdrought T2 -2.39395    0.14378 -16.650 8.67e-13 ***
## treatmentdrought T3 -4.35278    0.14378 -30.273  < 2e-16 ***
## treatmentdrought T4 -6.38937    0.15530 -41.141  < 2e-16 ***
## treatmentdrought T5 -9.62466    0.14378 -66.938  < 2e-16 ***
## treatmentrewetting   0.05093    0.14378   0.354 0.727056    
## ---
## Signif. codes:  0 '***' 0.001 '**' 0.01 '*' 0.05 '.' 0.1 ' ' 1
## 
## Residual standard error: 0.2033 on 19 degrees of freedom
## Multiple R-squared:  0.9974, Adjusted R-squared:  0.9966 
## F-statistic:  1236 on 6 and 19 DF,  p-value: < 2.2e-16
```

```
HSD.test(mod1,"treatment",group=T,unbalanced = T)$groups
```

```
mod1 <- lm(DOC ~ treatment , data = sample) 
any(sample$DOC==0)
```

```
## [1] FALSE
```

```
mod1l <- lm(log(DOC) ~ treatment, data = sample) 
mod1sq <- lm(sqrt(DOC) ~ treatment, data = sample) 
plot(mod1)# best
plot(mod1l)
plot(mod1sq)
```

```
summary(mod1)
```

```
## 
## Call:
## lm(formula = DOC ~ treatment, data = sample)
## 
## Residuals:
##     Min      1Q  Median      3Q     Max 
## -37.140 -10.874   3.116   6.407  65.680 
## 
## Coefficients:
##                     Estimate Std. Error t value Pr(>|t|)    
## (Intercept)           105.68      11.49   9.200 1.98e-08 ***
## treatmentdrought T1    16.31      17.55   0.929  0.36439    
## treatmentdrought T2    29.10      16.24   1.791  0.08921 .  
## treatmentdrought T3    56.36      16.24   3.470  0.00257 ** 
## treatmentdrought T4   -44.46      17.55  -2.534  0.02025 *  
## treatmentdrought T5   -32.74      16.24  -2.016  0.05821 .  
## treatmentrewetting    -31.70      16.24  -1.951  0.06593 .  
## ---
## Signif. codes:  0 '***' 0.001 '**' 0.01 '*' 0.05 '.' 0.1 ' ' 1
## 
## Residual standard error: 22.97 on 19 degrees of freedom
## Multiple R-squared:  0.7563, Adjusted R-squared:  0.6794 
## F-statistic:  9.83 on 6 and 19 DF,  p-value: 5.486e-05
```

```
summary(mod1l)
```

```
## 
## Call:
## lm(formula = log(DOC) ~ treatment, data = sample)
## 
## Residuals:
##      Min       1Q   Median       3Q      Max 
## -0.41059 -0.09688  0.02931  0.09138  0.36673 
## 
## Coefficients:
##                     Estimate Std. Error t value Pr(>|t|)    
## (Intercept)          4.65840    0.09797  47.548  < 2e-16 ***
## treatmentdrought T1  0.14464    0.14966   0.966  0.34595    
## treatmentdrought T2  0.23211    0.13855   1.675  0.11027    
## treatmentdrought T3  0.40299    0.13855   2.909  0.00901 ** 
## treatmentdrought T4 -0.55659    0.14966  -3.719  0.00145 ** 
## treatmentdrought T5 -0.37986    0.13855  -2.742  0.01297 *  
## treatmentrewetting  -0.38141    0.13855  -2.753  0.01266 *  
## ---
## Signif. codes:  0 '***' 0.001 '**' 0.01 '*' 0.05 '.' 0.1 ' ' 1
## 
## Residual standard error: 0.1959 on 19 degrees of freedom
## Multiple R-squared:  0.799,  Adjusted R-squared:  0.7355 
## F-statistic: 12.59 on 6 and 19 DF,  p-value: 9.707e-06
```

```
summary(mod1sq)
```

```
## 
## Call:
## lm(formula = sqrt(DOC) ~ treatment, data = sample)
## 
## Residuals:
##     Min      1Q  Median      3Q     Max 
## -1.6348 -0.5113  0.1606  0.3713  2.4468 
## 
## Coefficients:
##                     Estimate Std. Error t value Pr(>|t|)    
## (Intercept)          10.2749     0.5040  20.387 2.25e-14 ***
## treatmentdrought T1   0.7674     0.7698   0.997  0.33138    
## treatmentdrought T2   1.2963     0.7127   1.819  0.08474 .  
## treatmentdrought T3   2.3687     0.7127   3.323  0.00357 ** 
## treatmentdrought T4  -2.4753     0.7698  -3.215  0.00455 ** 
## treatmentdrought T5  -1.7575     0.7127  -2.466  0.02336 *  
## treatmentrewetting   -1.7285     0.7127  -2.425  0.02543 *  
## ---
## Signif. codes:  0 '***' 0.001 '**' 0.01 '*' 0.05 '.' 0.1 ' ' 1
## 
## Residual standard error: 1.008 on 19 degrees of freedom
## Multiple R-squared:  0.7906, Adjusted R-squared:  0.7244 
## F-statistic: 11.95 on 6 and 19 DF,  p-value: 1.407e-05
```

```
HSD.test(mod1,"treatment",group=T,unbalanced = T)$groups
```

```
mod1 <- lm(DON ~ treatment , data = sample) 
any(sample$DON==0)
```

```
## [1] FALSE
```

```
mod1l <- lm(log(DON) ~ treatment, data = sample) 
mod1sq <- lm(sqrt(DON) ~ treatment, data = sample) 
plot(mod1)# best
plot(mod1l)
plot(mod1sq)
```

```
summary(mod1)
```

```
## 
## Call:
## lm(formula = DON ~ treatment, data = sample)
## 
## Residuals:
##     Min      1Q  Median      3Q     Max 
## -8.7400 -2.6438  0.2175  0.9675 19.8900 
## 
## Coefficients:
##                     Estimate Std. Error t value Pr(>|t|)    
## (Intercept)          31.2175     2.9927  10.431 2.65e-09 ***
## treatmentdrought T1   0.6558     4.5714   0.143  0.88743    
## treatmentdrought T2   4.2200     4.2323   0.997  0.33125    
## treatmentdrought T3  14.0425     4.2323   3.318  0.00362 ** 
## treatmentdrought T4 -13.7008     4.5714  -2.997  0.00741 ** 
## treatmentdrought T5 -10.8075     4.2323  -2.554  0.01941 *  
## treatmentrewetting   -5.7250     4.2323  -1.353  0.19203    
## ---
## Signif. codes:  0 '***' 0.001 '**' 0.01 '*' 0.05 '.' 0.1 ' ' 1
## 
## Residual standard error: 5.985 on 19 degrees of freedom
## Multiple R-squared:  0.7444, Adjusted R-squared:  0.6637 
## F-statistic: 9.225 on 6 and 19 DF,  p-value: 8.393e-05
```

```
summary(mod1l)
```

```
## 
## Call:
## lm(formula = log(DON) ~ treatment, data = sample)
## 
## Residuals:
##      Min       1Q   Median       3Q      Max 
## -0.25637 -0.09344  0.00952  0.03874  0.39283 
## 
## Coefficients:
##                     Estimate Std. Error t value Pr(>|t|)    
## (Intercept)          3.43919    0.08059  42.677  < 2e-16 ***
## treatmentdrought T1  0.02206    0.12310   0.179  0.85966    
## treatmentdrought T2  0.12449    0.11397   1.092  0.28835    
## treatmentdrought T3  0.34467    0.11397   3.024  0.00698 ** 
## treatmentdrought T4 -0.59782    0.12310  -4.856  0.00011 ***
## treatmentdrought T5 -0.43427    0.11397  -3.811  0.00118 ** 
## treatmentrewetting  -0.20230    0.11397  -1.775  0.09191 .  
## ---
## Signif. codes:  0 '***' 0.001 '**' 0.01 '*' 0.05 '.' 0.1 ' ' 1
## 
## Residual standard error: 0.1612 on 19 degrees of freedom
## Multiple R-squared:  0.8238, Adjusted R-squared:  0.7682 
## F-statistic: 14.81 on 6 and 19 DF,  p-value: 2.929e-06
```

```
summary(mod1sq)
```

```
## 
## Call:
## lm(formula = sqrt(DON) ~ treatment, data = sample)
## 
## Residuals:
##      Min       1Q   Median       3Q      Max 
## -0.63489 -0.24817  0.02321  0.09678  1.39349 
## 
## Coefficients:
##                     Estimate Std. Error t value Pr(>|t|)    
## (Intercept)          5.58477    0.23440  23.826 1.29e-15 ***
## treatmentdrought T1  0.06015    0.35805   0.168 0.868369    
## treatmentdrought T2  0.36203    0.33149   1.092 0.288426    
## treatmentdrought T3  1.09329    0.33149   3.298 0.003781 ** 
## treatmentdrought T4 -1.42260    0.35805  -3.973 0.000815 ***
## treatmentdrought T5 -1.07922    0.33149  -3.256 0.004160 ** 
## treatmentrewetting  -0.53762    0.33149  -1.622 0.121311    
## ---
## Signif. codes:  0 '***' 0.001 '**' 0.01 '*' 0.05 '.' 0.1 ' ' 1
## 
## Residual standard error: 0.4688 on 19 degrees of freedom
## Multiple R-squared:  0.7965, Adjusted R-squared:  0.7322 
## F-statistic: 12.39 on 6 and 19 DF,  p-value: 1.087e-05
```

```
HSD.test(mod1,"treatment",group=T,unbalanced = T)$groups
```

```
mod1 <- lm(NH4 ~ treatment , data = sample) 
any(sample$NH4==0)
```

```
## [1] FALSE
```

```
mod1l <- lm(log(NH4) ~ treatment, data = sample) 
mod1sq <- lm(sqrt(NH4) ~ treatment, data = sample) 
plot(mod1)# best
plot(mod1l)
plot(mod1sq)
```

```
summary(mod1)
```

```
## 
## Call:
## lm(formula = NH4 ~ treatment, data = sample)
## 
## Residuals:
##     Min      1Q  Median      3Q     Max 
## -21.500 -11.623   2.030   9.058  25.860 
## 
## Coefficients:
##                     Estimate Std. Error t value Pr(>|t|)    
## (Intercept)          101.110      6.984  14.478 1.02e-11 ***
## treatmentdrought T1  -13.580     10.668  -1.273   0.2184    
## treatmentdrought T2   -5.088      9.876  -0.515   0.6124    
## treatmentdrought T3    2.720      9.876   0.275   0.7860    
## treatmentdrought T4  -13.610     10.668  -1.276   0.2174    
## treatmentdrought T5  -22.903      9.876  -2.319   0.0317 *  
## treatmentrewetting    16.960      9.876   1.717   0.1022    
## ---
## Signif. codes:  0 '***' 0.001 '**' 0.01 '*' 0.05 '.' 0.1 ' ' 1
## 
## Residual standard error: 13.97 on 19 degrees of freedom
## Multiple R-squared:  0.518,  Adjusted R-squared:  0.3658 
## F-statistic: 3.404 on 6 and 19 DF,  p-value: 0.01881
```

```
summary(mod1l)
```

```
## 
## Call:
## lm(formula = log(NH4) ~ treatment, data = sample)
## 
## Residuals:
##      Min       1Q   Median       3Q      Max 
## -0.20300 -0.11490  0.02518  0.10802  0.20911 
## 
## Coefficients:
##                     Estimate Std. Error t value Pr(>|t|)    
## (Intercept)           4.6080     0.0712  64.722   <2e-16 ***
## treatmentdrought T1  -0.1460     0.1087  -1.342   0.1953    
## treatmentdrought T2  -0.0476     0.1007  -0.473   0.6418    
## treatmentdrought T3   0.0289     0.1007   0.287   0.7772    
## treatmentdrought T4  -0.1440     0.1087  -1.324   0.2012    
## treatmentdrought T5  -0.2536     0.1007  -2.519   0.0209 *  
## treatmentrewetting    0.1522     0.1007   1.512   0.1470    
## ---
## Signif. codes:  0 '***' 0.001 '**' 0.01 '*' 0.05 '.' 0.1 ' ' 1
## 
## Residual standard error: 0.1424 on 19 degrees of freedom
## Multiple R-squared:  0.5206, Adjusted R-squared:  0.3693 
## F-statistic: 3.439 on 6 and 19 DF,  p-value: 0.01801
```

```
summary(mod1sq)
```

```
## 
## Call:
## lm(formula = sqrt(NH4) ~ treatment, data = sample)
## 
## Residuals:
##     Min      1Q  Median      3Q     Max 
## -1.0089 -0.5747  0.1138  0.4941  1.1611 
## 
## Coefficients:
##                     Estimate Std. Error t value Pr(>|t|)    
## (Intercept)          10.0347     0.3501  28.662   <2e-16 ***
## treatmentdrought T1  -0.7018     0.5348  -1.312   0.2050    
## treatmentdrought T2  -0.2460     0.4951  -0.497   0.6250    
## treatmentdrought T3   0.1402     0.4951   0.283   0.7801    
## treatmentdrought T4  -0.6982     0.5348  -1.306   0.2073    
## treatmentdrought T5  -1.2021     0.4951  -2.428   0.0253 *  
## treatmentrewetting    0.8012     0.4951   1.618   0.1221    
## ---
## Signif. codes:  0 '***' 0.001 '**' 0.01 '*' 0.05 '.' 0.1 ' ' 1
## 
## Residual standard error: 0.7002 on 19 degrees of freedom
## Multiple R-squared:  0.5206, Adjusted R-squared:  0.3693 
## F-statistic: 3.439 on 6 and 19 DF,  p-value: 0.01801
```

```
HSD.test(mod1,"treatment",group=T,unbalanced = T)$groups
```

```
mod1 <- lm(NO3 ~ treatment , data = sample) 
any(sample$NO3==0)
```

```
## [1] FALSE
```

```
mod1l <- lm(log(NO3) ~ treatment, data = sample) 
mod1sq <- lm(sqrt(NO3) ~ treatment, data = sample) 
plot(mod1)# best
plot(mod1l)
plot(mod1sq)
```

```
summary(mod1)
```

```
## 
## Call:
## lm(formula = NO3 ~ treatment, data = sample)
## 
## Residuals:
##     Min      1Q  Median      3Q     Max 
## -7.9700 -2.6169 -0.4162  2.5750  7.0250 
## 
## Coefficients:
##                     Estimate Std. Error t value Pr(>|t|)    
## (Intercept)           18.487      2.185   8.461 7.22e-08 ***
## treatmentdrought T1    5.343      3.338   1.601   0.1259    
## treatmentdrought T2    8.005      3.090   2.591   0.0179 *  
## treatmentdrought T3   18.698      3.090   6.051 8.06e-06 ***
## treatmentdrought T4    8.333      3.338   2.497   0.0219 *  
## treatmentdrought T5    2.243      3.090   0.726   0.4768    
## treatmentrewetting     6.088      3.090   1.970   0.0636 .  
## ---
## Signif. codes:  0 '***' 0.001 '**' 0.01 '*' 0.05 '.' 0.1 ' ' 1
## 
## Residual standard error: 4.37 on 19 degrees of freedom
## Multiple R-squared:  0.702,  Adjusted R-squared:  0.6079 
## F-statistic:  7.46 on 6 and 19 DF,  p-value: 0.0003261
```

```
summary(mod1l)
```

```
## 
## Call:
## lm(formula = log(NO3) ~ treatment, data = sample)
## 
## Residuals:
##      Min       1Q   Median       3Q      Max 
## -0.32742 -0.07352 -0.00826  0.12500  0.27325 
## 
## Coefficients:
##                     Estimate Std. Error t value Pr(>|t|)    
## (Intercept)          2.91138    0.08853  32.884  < 2e-16 ***
## treatmentdrought T1  0.25736    0.13524   1.903  0.07231 .  
## treatmentdrought T2  0.36303    0.12521   2.899  0.00919 ** 
## treatmentdrought T3  0.69853    0.12521   5.579 2.22e-05 ***
## treatmentdrought T4  0.35255    0.13524   2.607  0.01733 *  
## treatmentdrought T5  0.10721    0.12521   0.856  0.40251    
## treatmentrewetting   0.26536    0.12521   2.119  0.04746 *  
## ---
## Signif. codes:  0 '***' 0.001 '**' 0.01 '*' 0.05 '.' 0.1 ' ' 1
## 
## Residual standard error: 0.1771 on 19 degrees of freedom
## Multiple R-squared:  0.6638, Adjusted R-squared:  0.5577 
## F-statistic: 6.253 on 6 and 19 DF,  p-value: 0.0009312
```

```
summary(mod1sq)
```

```
## 
## Call:
## lm(formula = sqrt(NO3) ~ treatment, data = sample)
## 
## Residuals:
##      Min       1Q   Median       3Q      Max 
## -0.80568 -0.21852 -0.03017  0.28214  0.68592 
## 
## Coefficients:
##                     Estimate Std. Error t value Pr(>|t|)    
## (Intercept)           4.2937     0.2172  19.764 3.95e-14 ***
## treatmentdrought T1   0.5852     0.3319   1.764   0.0939 .  
## treatmentdrought T2   0.8503     0.3072   2.767   0.0123 *  
## treatmentdrought T3   1.7950     0.3072   5.842 1.26e-05 ***
## treatmentdrought T4   0.8537     0.3319   2.572   0.0186 *  
## treatmentdrought T5   0.2449     0.3072   0.797   0.4353    
## treatmentrewetting    0.6329     0.3072   2.060   0.0534 .  
## ---
## Signif. codes:  0 '***' 0.001 '**' 0.01 '*' 0.05 '.' 0.1 ' ' 1
## 
## Residual standard error: 0.4345 on 19 degrees of freedom
## Multiple R-squared:  0.685,  Adjusted R-squared:  0.5856 
## F-statistic: 6.888 on 6 and 19 DF,  p-value: 0.0005291
```

```
HSD.test(mod1,"treatment",group=T,unbalanced = T)$groups
```

```
mod1 <- lm(PO4 ~ treatment , data = sample) 
any(sample$PO4==0)
```

```
## [1] FALSE
```

```
mod1l <- lm(log(PO4) ~ treatment, data = sample) 
mod1sq <- lm(sqrt(PO4) ~ treatment, data = sample) 
plot(mod1)
plot(mod1l)
plot(mod1sq)
```

```
summary(mod1)
```

```
## 
## Call:
## lm(formula = PO4 ~ treatment, data = sample)
## 
## Residuals:
##     Min      1Q  Median      3Q     Max 
## -1.0100 -0.2610  0.0600  0.2006  1.6700 
## 
## Coefficients:
##                     Estimate Std. Error t value Pr(>|t|)    
## (Intercept)           1.1975     0.2850   4.201 0.000484 ***
## treatmentdrought T1   0.4625     0.4354   1.062 0.301447    
## treatmentdrought T2  -0.0150     0.4031  -0.037 0.970705    
## treatmentdrought T3  -0.3425     0.4031  -0.850 0.406098    
## treatmentdrought T4  -0.4708     0.4354  -1.081 0.293067    
## treatmentdrought T5  -0.5050     0.4031  -1.253 0.225489    
## treatmentrewetting   -0.3975     0.4031  -0.986 0.336479    
## ---
## Signif. codes:  0 '***' 0.001 '**' 0.01 '*' 0.05 '.' 0.1 ' ' 1
## 
## Residual standard error: 0.5701 on 19 degrees of freedom
## Multiple R-squared:  0.2834, Adjusted R-squared:  0.05714 
## F-statistic: 1.253 on 6 and 19 DF,  p-value: 0.3246
```

```
summary(mod1l)
```

```
## 
## Call:
## lm(formula = log(PO4) ~ treatment, data = sample)
## 
## Residuals:
##      Min       1Q   Median       3Q      Max 
## -0.68818 -0.31884  0.08866  0.25226  0.94558 
## 
## Coefficients:
##                     Estimate Std. Error t value Pr(>|t|)  
## (Intercept)          0.14193    0.21926   0.647    0.525  
## treatmentdrought T1  0.11547    0.33492   0.345    0.734  
## treatmentdrought T2 -0.07001    0.31008  -0.226    0.824  
## treatmentdrought T3 -0.32215    0.31008  -1.039    0.312  
## treatmentdrought T4 -0.49942    0.33492  -1.491    0.152  
## treatmentdrought T5 -0.57342    0.31008  -1.849    0.080 .
## treatmentrewetting  -0.38148    0.31008  -1.230    0.234  
## ---
## Signif. codes:  0 '***' 0.001 '**' 0.01 '*' 0.05 '.' 0.1 ' ' 1
## 
## Residual standard error: 0.4385 on 19 degrees of freedom
## Multiple R-squared:  0.2876, Adjusted R-squared:  0.06268 
## F-statistic: 1.279 on 6 and 19 DF,  p-value: 0.3133
```

```
summary(mod1sq)# best
```

```
## 
## Call:
## lm(formula = sqrt(PO4) ~ treatment, data = sample)
## 
## Residuals:
##      Min       1Q   Median       3Q      Max 
## -0.40413 -0.15712  0.03644  0.11116  0.61448 
## 
## Coefficients:
##                     Estimate Std. Error t value Pr(>|t|)    
## (Intercept)          1.08435    0.11919   9.098 2.36e-08 ***
## treatmentdrought T1  0.12600    0.18206   0.692    0.497    
## treatmentdrought T2 -0.02225    0.16856  -0.132    0.896    
## treatmentdrought T3 -0.16498    0.16856  -0.979    0.340    
## treatmentdrought T4 -0.23970    0.18206  -1.317    0.204    
## treatmentdrought T5 -0.26520    0.16856  -1.573    0.132    
## treatmentrewetting  -0.19344    0.16856  -1.148    0.265    
## ---
## Signif. codes:  0 '***' 0.001 '**' 0.01 '*' 0.05 '.' 0.1 ' ' 1
## 
## Residual standard error: 0.2384 on 19 degrees of freedom
## Multiple R-squared:  0.2862, Adjusted R-squared:  0.06079 
## F-statistic:  1.27 on 6 and 19 DF,  p-value: 0.3172
```

```
HSD.test(mod1sq,"treatment",group=T,unbalanced = T)$groups
```

```
mod1 <- lm(RNA_DW ~ treatment , data = sample) 
any(sample$RNA_DW==0)
```

```
## [1] FALSE
```

```
mod1l <- lm(log(RNA_DW) ~ treatment, data = sample) 
mod1sq <- lm(sqrt(RNA_DW) ~ treatment, data = sample) 
plot(mod1)
plot(mod1l)# best
plot(mod1sq)
```

```
summary(mod1)
```

```
## 
## Call:
## lm(formula = RNA_DW ~ treatment, data = sample)
## 
## Residuals:
##     Min      1Q  Median      3Q     Max 
## -8006.9 -1194.5   -74.1  1198.8 10794.9 
## 
## Coefficients:
##                     Estimate Std. Error t value Pr(>|t|)    
## (Intercept)            20059       1785  11.235 7.81e-10 ***
## treatmentdrought T1   -12167       2727  -4.462 0.000268 ***
## treatmentdrought T2   -16659       2525  -6.598 2.58e-06 ***
## treatmentdrought T3   -17485       2525  -6.925 1.33e-06 ***
## treatmentdrought T4   -17813       2727  -6.532 2.96e-06 ***
## treatmentdrought T5   -16029       2525  -6.349 4.32e-06 ***
## treatmentrewetting    -14717       2525  -5.829 1.29e-05 ***
## ---
## Signif. codes:  0 '***' 0.001 '**' 0.01 '*' 0.05 '.' 0.1 ' ' 1
## 
## Residual standard error: 3571 on 19 degrees of freedom
## Multiple R-squared:  0.7925, Adjusted R-squared:  0.7269 
## F-statistic: 12.09 on 6 and 19 DF,  p-value: 1.297e-05
```

```
summary(mod1l)
```

```
## 
## Call:
## lm(formula = log(RNA_DW) ~ treatment, data = sample)
## 
## Residuals:
##     Min      1Q  Median      3Q     Max 
## -1.1672 -0.2691  0.0818  0.2997  0.8026 
## 
## Coefficients:
##                     Estimate Std. Error t value Pr(>|t|)    
## (Intercept)           9.8459     0.2467  39.917  < 2e-16 ***
## treatmentdrought T1  -0.9159     0.3768  -2.431 0.025139 *  
## treatmentdrought T2  -1.7893     0.3488  -5.130 5.96e-05 ***
## treatmentdrought T3  -2.2042     0.3488  -6.319 4.59e-06 ***
## treatmentdrought T4  -2.2373     0.3768  -5.938 1.02e-05 ***
## treatmentdrought T5  -1.5950     0.3488  -4.572 0.000208 ***
## treatmentrewetting   -1.2829     0.3488  -3.678 0.001598 ** 
## ---
## Signif. codes:  0 '***' 0.001 '**' 0.01 '*' 0.05 '.' 0.1 ' ' 1
## 
## Residual standard error: 0.4933 on 19 degrees of freedom
## Multiple R-squared:  0.7522, Adjusted R-squared:  0.6739 
## F-statistic: 9.612 on 6 and 19 DF,  p-value: 6.38e-05
```

```
summary(mod1sq)
```

```
## 
## Call:
## lm(formula = sqrt(RNA_DW) ~ treatment, data = sample)
## 
## Residuals:
##     Min      1Q  Median      3Q     Max 
## -29.723 -10.425   1.356   9.605  36.148 
## 
## Coefficients:
##                     Estimate Std. Error t value Pr(>|t|)    
## (Intercept)          139.505      8.338  16.731 7.95e-13 ***
## treatmentdrought T1  -51.606     12.737  -4.052 0.000681 ***
## treatmentdrought T2  -82.297     11.792  -6.979 1.20e-06 ***
## treatmentdrought T3  -91.093     11.792  -7.725 2.81e-07 ***
## treatmentdrought T4  -93.381     12.737  -7.331 5.97e-07 ***
## treatmentdrought T5  -76.801     11.792  -6.513 3.07e-06 ***
## treatmentrewetting   -66.789     11.792  -5.664 1.85e-05 ***
## ---
## Signif. codes:  0 '***' 0.001 '**' 0.01 '*' 0.05 '.' 0.1 ' ' 1
## 
## Residual standard error: 16.68 on 19 degrees of freedom
## Multiple R-squared:  0.8216, Adjusted R-squared:  0.7653 
## F-statistic: 14.59 on 6 and 19 DF,  p-value: 3.281e-06
```

```
HSD.test(mod1l,"treatment",group=T,unbalanced = T)$groups
```

```
mod1 <- lm(Laccase ~ treatment , data = sample) 
any(sample$Laccase==0)
```

```
## [1] TRUE
```

```
mod1l <- lm(log(Laccase+psc) ~ treatment, data = sample) 
mod1sq <- lm(sqrt(Laccase) ~ treatment, data = sample) 
plot(mod1)# best
plot(mod1l)
plot(mod1sq)
```

```
summary(mod1)
```

```
## 
## Call:
## lm(formula = Laccase ~ treatment, data = sample)
## 
## Residuals:
##     Min      1Q  Median      3Q     Max 
## -0.8075 -0.2263 -0.0250  0.2033  0.8525 
## 
## Coefficients:
##                     Estimate Std. Error t value Pr(>|t|)    
## (Intercept)           1.8750     0.2550   7.352 5.74e-07 ***
## treatmentdrought T1  -0.6650     0.3896  -1.707 0.104096    
## treatmentdrought T2  -0.5375     0.3607  -1.490 0.152549    
## treatmentdrought T3  -1.2200     0.3607  -3.383 0.003124 ** 
## treatmentdrought T4  -1.5683     0.3896  -4.026 0.000722 ***
## treatmentdrought T5  -1.8700     0.3607  -5.185 5.27e-05 ***
## treatmentrewetting   -1.2700     0.3607  -3.521 0.002282 ** 
## ---
## Signif. codes:  0 '***' 0.001 '**' 0.01 '*' 0.05 '.' 0.1 ' ' 1
## 
## Residual standard error: 0.51 on 19 degrees of freedom
## Multiple R-squared:  0.6618, Adjusted R-squared:  0.555 
## F-statistic: 6.196 on 6 and 19 DF,  p-value: 0.0009814
```

```
summary(mod1l)
```

```
## 
## Call:
## lm(formula = log(Laccase + psc) ~ treatment, data = sample)
## 
## Residuals:
##      Min       1Q   Median       3Q      Max 
## -18.7364  -0.6726  -0.0321   1.0126  16.0623 
## 
## Coefficients:
##                     Estimate Std. Error t value Pr(>|t|)   
## (Intercept)           0.6106     4.0041   0.152  0.88040   
## treatmentdrought T1  -0.4387     6.1164  -0.072  0.94357   
## treatmentdrought T2  -0.4895     5.6627  -0.086  0.93202   
## treatmentdrought T3  -1.3546     5.6627  -0.239  0.81350   
## treatmentdrought T4  -9.5873     6.1164  -1.567  0.13351   
## treatmentdrought T5 -20.5850     5.6627  -3.635  0.00176 **
## treatmentrewetting   -7.2026     5.6627  -1.272  0.21874   
## ---
## Signif. codes:  0 '***' 0.001 '**' 0.01 '*' 0.05 '.' 0.1 ' ' 1
## 
## Residual standard error: 8.008 on 19 degrees of freedom
## Multiple R-squared:  0.5233, Adjusted R-squared:  0.3728 
## F-statistic: 3.476 on 6 and 19 DF,  p-value: 0.01722
```

```
summary(mod1sq)
```

```
## 
## Call:
## lm(formula = sqrt(Laccase) ~ treatment, data = sample)
## 
## Residuals:
##      Min       1Q   Median       3Q      Max 
## -0.65144 -0.09942 -0.01731  0.15047  0.53600 
## 
## Coefficients:
##                     Estimate Std. Error t value Pr(>|t|)    
## (Intercept)           1.3631     0.1596   8.543 6.23e-08 ***
## treatmentdrought T1  -0.2683     0.2437  -1.101  0.28472    
## treatmentdrought T2  -0.2524     0.2256  -1.118  0.27732    
## treatmentdrought T3  -0.6124     0.2256  -2.714  0.01377 *  
## treatmentdrought T4  -0.9136     0.2437  -3.749  0.00136 ** 
## treatmentdrought T5  -1.3277     0.2256  -5.884 1.15e-05 ***
## treatmentrewetting   -0.7117     0.2256  -3.154  0.00523 ** 
## ---
## Signif. codes:  0 '***' 0.001 '**' 0.01 '*' 0.05 '.' 0.1 ' ' 1
## 
## Residual standard error: 0.3191 on 19 degrees of freedom
## Multiple R-squared:  0.7088, Adjusted R-squared:  0.6169 
## F-statistic: 7.709 on 6 and 19 DF,  p-value: 0.0002661
```

```
HSD.test(mod1,"treatment",group=T,unbalanced = T)$groups
```

```
mod1 <- lm(Cellulase ~ treatment , data = sample) 
any(sample$Cellulase==0)
```

```
## [1] TRUE
```

```
mod1l <- lm(log(Cellulase+psc) ~ treatment, data = sample) 
mod1sq <- lm(sqrt(Cellulase) ~ treatment, data = sample) 
plot(mod1)# best
plot(mod1l)
plot(mod1sq)
```

```
summary(mod1)
```

```
## 
## Call:
## lm(formula = Cellulase ~ treatment, data = sample)
## 
## Residuals:
##    Min     1Q Median     3Q    Max 
## -6.140 -0.260  0.000  1.316  3.127 
## 
## Coefficients:
##                     Estimate Std. Error t value Pr(>|t|)    
## (Intercept)            9.380      1.106   8.482 6.95e-08 ***
## treatmentdrought T1   -2.613      1.689  -1.547  0.13833    
## treatmentdrought T2   -5.057      1.564  -3.234  0.00437 ** 
## treatmentdrought T3   -7.830      1.564  -5.007 7.83e-05 ***
## treatmentdrought T4   -9.380      1.689  -5.553 2.35e-05 ***
## treatmentdrought T5   -9.257      1.564  -5.920 1.07e-05 ***
## treatmentrewetting    -9.030      1.564  -5.774 1.45e-05 ***
## ---
## Signif. codes:  0 '***' 0.001 '**' 0.01 '*' 0.05 '.' 0.1 ' ' 1
## 
## Residual standard error: 2.212 on 19 degrees of freedom
## Multiple R-squared:  0.7682, Adjusted R-squared:  0.695 
## F-statistic: 10.49 on 6 and 19 DF,  p-value: 3.513e-05
```

```
summary(mod1l)
```

```
## 
## Call:
## lm(formula = log(Cellulase + psc) ~ treatment, data = sample)
## 
## Residuals:
##      Min       1Q   Median       3Q      Max 
## -20.2888  -1.2001   0.1076   4.1449  18.4613 
## 
## Coefficients:
##                     Estimate Std. Error t value Pr(>|t|)    
## (Intercept)           2.1207     4.3533   0.487 0.631730    
## treatmentdrought T1  -0.2229     6.6498  -0.034 0.973612    
## treatmentdrought T2  -7.1603     6.1566  -1.163 0.259214    
## treatmentdrought T3  -2.3497     6.1566  -0.382 0.706945    
## treatmentdrought T4 -27.4491     6.6498  -4.128 0.000572 ***
## treatmentdrought T5 -21.2954     6.1566  -3.459 0.002629 ** 
## treatmentrewetting   -9.6071     6.1566  -1.560 0.135153    
## ---
## Signif. codes:  0 '***' 0.001 '**' 0.01 '*' 0.05 '.' 0.1 ' ' 1
## 
## Residual standard error: 8.707 on 19 degrees of freedom
## Multiple R-squared:  0.6218, Adjusted R-squared:  0.5024 
## F-statistic: 5.207 on 6 and 19 DF,  p-value: 0.002547
```

```
summary(mod1sq)
```

```
## 
## Call:
## lm(formula = sqrt(Cellulase) ~ treatment, data = sample)
## 
## Residuals:
##     Min      1Q  Median      3Q     Max 
## -1.7880 -0.1750  0.0000  0.4261  0.9415 
## 
## Coefficients:
##                     Estimate Std. Error t value Pr(>|t|)    
## (Intercept)           2.9851     0.3455   8.639 5.25e-08 ***
## treatmentdrought T1  -0.3930     0.5278  -0.745 0.465639    
## treatmentdrought T2  -1.1971     0.4886  -2.450 0.024156 *  
## treatmentdrought T3  -1.9024     0.4886  -3.893 0.000978 ***
## treatmentdrought T4  -2.9851     0.5278  -5.656 1.88e-05 ***
## treatmentdrought T5  -2.8101     0.4886  -5.751 1.53e-05 ***
## treatmentrewetting   -2.5590     0.4886  -5.237 4.70e-05 ***
## ---
## Signif. codes:  0 '***' 0.001 '**' 0.01 '*' 0.05 '.' 0.1 ' ' 1
## 
## Residual standard error: 0.6911 on 19 degrees of freedom
## Multiple R-squared:  0.7718, Adjusted R-squared:  0.6998 
## F-statistic: 10.71 on 6 and 19 DF,  p-value: 3.045e-05
```

```
HSD.test(mod1,"treatment",group=T,unbalanced = T)$groups
```

```
mod1 <- lm(CO2 ~ treatment , data = sample) 
any(sample$CO2==0)
```

```
## [1] FALSE
```

```
mod1l <- lm(log(CO2) ~ treatment, data = sample) 
mod1sq <- lm(sqrt(CO2) ~ treatment, data = sample) 
plot(mod1)# best
plot(mod1l)
plot(mod1sq)
```

```
summary(mod1)
```

```
## 
## Call:
## lm(formula = CO2 ~ treatment, data = sample)
## 
## Residuals:
##      Min       1Q   Median       3Q      Max 
## -13.3133  -0.7906   0.4691   1.2858   6.7675 
## 
## Coefficients:
##                     Estimate Std. Error t value Pr(>|t|)    
## (Intercept)           28.171      1.991  14.149 1.53e-11 ***
## treatmentdrought T1   -2.653      3.041  -0.872  0.39388    
## treatmentdrought T2   -5.922      2.816  -2.103  0.04900 *  
## treatmentdrought T3  -10.296      2.816  -3.656  0.00168 ** 
## treatmentdrought T4  -19.045      3.041  -6.262 5.17e-06 ***
## treatmentdrought T5  -27.454      2.816  -9.750 7.90e-09 ***
## treatmentrewetting    -7.339      2.816  -2.606  0.01735 *  
## ---
## Signif. codes:  0 '***' 0.001 '**' 0.01 '*' 0.05 '.' 0.1 ' ' 1
## 
## Residual standard error: 3.982 on 19 degrees of freedom
## Multiple R-squared:  0.8755, Adjusted R-squared:  0.8361 
## F-statistic: 22.26 on 6 and 19 DF,  p-value: 1.21e-07
```

```
summary(mod1l)
```

```
## 
## Call:
## lm(formula = log(CO2) ~ treatment, data = sample)
## 
## Residuals:
##      Min       1Q   Median       3Q      Max 
## -0.90624 -0.04284  0.03931  0.13043  0.39409 
## 
## Coefficients:
##                     Estimate Std. Error t value Pr(>|t|)    
## (Intercept)          3.33784    0.16287  20.494 2.04e-14 ***
## treatmentdrought T1 -0.09959    0.24878  -0.400 0.693387    
## treatmentdrought T2 -0.23757    0.23033  -1.031 0.315275    
## treatmentdrought T3 -0.46244    0.23033  -2.008 0.059103 .  
## treatmentdrought T4 -1.16413    0.24878  -4.679 0.000163 ***
## treatmentdrought T5 -3.73081    0.23033 -16.198 1.41e-12 ***
## treatmentrewetting  -0.41410    0.23033  -1.798 0.088107 .  
## ---
## Signif. codes:  0 '***' 0.001 '**' 0.01 '*' 0.05 '.' 0.1 ' ' 1
## 
## Residual standard error: 0.3257 on 19 degrees of freedom
## Multiple R-squared:  0.953,  Adjusted R-squared:  0.9382 
## F-statistic: 64.23 on 6 and 19 DF,  p-value: 1.339e-11
```

```
summary(mod1sq)
```

```
## 
## Call:
## lm(formula = sqrt(CO2) ~ treatment, data = sample)
## 
## Residuals:
##      Min       1Q   Median       3Q      Max 
## -1.71003 -0.11052  0.05804  0.14752  0.80139 
## 
## Coefficients:
##                     Estimate Std. Error t value Pr(>|t|)    
## (Intercept)           5.3071     0.2579  20.581 1.89e-14 ***
## treatmentdrought T1  -0.2569     0.3939  -0.652  0.52201    
## treatmentdrought T2  -0.5926     0.3647  -1.625  0.12066    
## treatmentdrought T3  -1.0874     0.3647  -2.982  0.00766 ** 
## treatmentdrought T4  -2.3133     0.3939  -5.873 1.18e-05 ***
## treatmentdrought T5  -4.4718     0.3647 -12.262 1.80e-10 ***
## treatmentrewetting   -0.8548     0.3647  -2.344  0.03009 *  
## ---
## Signif. codes:  0 '***' 0.001 '**' 0.01 '*' 0.05 '.' 0.1 ' ' 1
## 
## Residual standard error: 0.5157 on 19 degrees of freedom
## Multiple R-squared:  0.9175, Adjusted R-squared:  0.8914 
## F-statistic:  35.2 on 6 and 19 DF,  p-value: 2.639e-09
```

```
HSD.test(mod1,"treatment",group=T,unbalanced = T)$groups
```

```
mod1 <- lm(CH4 ~ treatment , data = sample) 
any(sample$CH4==0)
```

```
## [1] FALSE
```

```
mod1l <- lm(log(CH4 + abs(min(CH4))+psc) ~ treatment, data = sample) 
mod1sq <- lm(cbrt(CH4) ~ treatment, data = sample) 
plot(mod1)# best
plot(mod1l)
plot(mod1sq)
```

```
summary(mod1)
```

```
## 
## Call:
## lm(formula = CH4 ~ treatment, data = sample)
## 
## Residuals:
##     Min      1Q  Median      3Q     Max 
## -526.74 -270.43  -33.59  179.83  801.66 
## 
## Coefficients:
##                     Estimate Std. Error t value Pr(>|t|)    
## (Intercept)         -1986.53     178.41 -11.135 9.07e-10 ***
## treatmentdrought T1  1314.15     272.53   4.822 0.000118 ***
## treatmentdrought T2   -92.44     252.31  -0.366 0.718141    
## treatmentdrought T3  -470.56     252.31  -1.865 0.077704 .  
## treatmentdrought T4  -437.50     272.53  -1.605 0.124913    
## treatmentdrought T5  2129.12     252.31   8.439 7.52e-08 ***
## treatmentrewetting   1283.72     252.31   5.088 6.54e-05 ***
## ---
## Signif. codes:  0 '***' 0.001 '**' 0.01 '*' 0.05 '.' 0.1 ' ' 1
## 
## Residual standard error: 356.8 on 19 degrees of freedom
## Multiple R-squared:  0.9078, Adjusted R-squared:  0.8787 
## F-statistic: 31.19 on 6 and 19 DF,  p-value: 7.387e-09
```

```
summary(mod1l)
```

```
## 
## Call:
## lm(formula = log(CH4 + abs(min(CH4)) + psc) ~ treatment, data = sample)
## 
## Residuals:
##      Min       1Q   Median       3Q      Max 
## -22.6641  -0.1165   0.0181   0.3548   9.0777 
## 
## Coefficients:
##                     Estimate Std. Error t value Pr(>|t|)  
## (Intercept)           6.5879     3.0434   2.165   0.0434 *
## treatmentdrought T1   1.0342     4.6488   0.222   0.8263  
## treatmentdrought T2  -0.2578     4.3040  -0.060   0.9529  
## treatmentdrought T3  -9.2522     4.3040  -2.150   0.0447 *
## treatmentdrought T4  -1.3642     4.6488  -0.293   0.7724  
## treatmentdrought T5   1.3775     4.3040   0.320   0.7524  
## treatmentrewetting    1.0017     4.3040   0.233   0.8185  
## ---
## Signif. codes:  0 '***' 0.001 '**' 0.01 '*' 0.05 '.' 0.1 ' ' 1
## 
## Residual standard error: 6.087 on 19 degrees of freedom
## Multiple R-squared:  0.3189, Adjusted R-squared:  0.1038 
## F-statistic: 1.483 on 6 and 19 DF,  p-value: 0.2369
```

```
summary(mod1sq)
```

```
## 
## Call:
## lm(formula = cbrt(CH4) ~ treatment, data = sample)
## 
## Residuals:
##     Min      1Q  Median      3Q     Max 
## -4.4934 -0.7047 -0.1914  0.3951 10.8435 
## 
## Coefficients:
##                     Estimate Std. Error t value Pr(>|t|)    
## (Intercept)         -12.5579     1.5141  -8.294 9.76e-08 ***
## treatmentdrought T1   4.0981     2.3128   1.772  0.09244 .  
## treatmentdrought T2  -0.1611     2.1412  -0.075  0.94083    
## treatmentdrought T3  -0.9165     2.1412  -0.428  0.67346    
## treatmentdrought T4  -0.8648     2.3128  -0.374  0.71260    
## treatmentdrought T5  17.6401     2.1412   8.238 1.08e-07 ***
## treatmentrewetting    6.3383     2.1412   2.960  0.00804 ** 
## ---
## Signif. codes:  0 '***' 0.001 '**' 0.01 '*' 0.05 '.' 0.1 ' ' 1
## 
## Residual standard error: 3.028 on 19 degrees of freedom
## Multiple R-squared:  0.8596, Adjusted R-squared:  0.8153 
## F-statistic: 19.39 on 6 and 19 DF,  p-value: 3.653e-07
```

```
HSD.test(mod1,"treatment",group=T,unbalanced = T)$groups
```

```
mod1 <- lm(CO2 ~ treatment , data = sample) 
any(sample$CO2==0)
```

```
## [1] FALSE
```

```
mod1l <- lm(log(CO2) ~ treatment, data = sample) 
mod1sq <- lm(sqrt(CO2) ~ treatment, data = sample) 
plot(mod1)# best
plot(mod1l)
plot(mod1sq)
```

```
summary(mod1)
```

```
## 
## Call:
## lm(formula = CO2 ~ treatment, data = sample)
## 
## Residuals:
##      Min       1Q   Median       3Q      Max 
## -13.3133  -0.7906   0.4691   1.2858   6.7675 
## 
## Coefficients:
##                     Estimate Std. Error t value Pr(>|t|)    
## (Intercept)           28.171      1.991  14.149 1.53e-11 ***
## treatmentdrought T1   -2.653      3.041  -0.872  0.39388    
## treatmentdrought T2   -5.922      2.816  -2.103  0.04900 *  
## treatmentdrought T3  -10.296      2.816  -3.656  0.00168 ** 
## treatmentdrought T4  -19.045      3.041  -6.262 5.17e-06 ***
## treatmentdrought T5  -27.454      2.816  -9.750 7.90e-09 ***
## treatmentrewetting    -7.339      2.816  -2.606  0.01735 *  
## ---
## Signif. codes:  0 '***' 0.001 '**' 0.01 '*' 0.05 '.' 0.1 ' ' 1
## 
## Residual standard error: 3.982 on 19 degrees of freedom
## Multiple R-squared:  0.8755, Adjusted R-squared:  0.8361 
## F-statistic: 22.26 on 6 and 19 DF,  p-value: 1.21e-07
```

```
summary(mod1l)
```

```
## 
## Call:
## lm(formula = log(CO2) ~ treatment, data = sample)
## 
## Residuals:
##      Min       1Q   Median       3Q      Max 
## -0.90624 -0.04284  0.03931  0.13043  0.39409 
## 
## Coefficients:
##                     Estimate Std. Error t value Pr(>|t|)    
## (Intercept)          3.33784    0.16287  20.494 2.04e-14 ***
## treatmentdrought T1 -0.09959    0.24878  -0.400 0.693387    
## treatmentdrought T2 -0.23757    0.23033  -1.031 0.315275    
## treatmentdrought T3 -0.46244    0.23033  -2.008 0.059103 .  
## treatmentdrought T4 -1.16413    0.24878  -4.679 0.000163 ***
## treatmentdrought T5 -3.73081    0.23033 -16.198 1.41e-12 ***
## treatmentrewetting  -0.41410    0.23033  -1.798 0.088107 .  
## ---
## Signif. codes:  0 '***' 0.001 '**' 0.01 '*' 0.05 '.' 0.1 ' ' 1
## 
## Residual standard error: 0.3257 on 19 degrees of freedom
## Multiple R-squared:  0.953,  Adjusted R-squared:  0.9382 
## F-statistic: 64.23 on 6 and 19 DF,  p-value: 1.339e-11
```

```
summary(mod1sq)
```

```
## 
## Call:
## lm(formula = sqrt(CO2) ~ treatment, data = sample)
## 
## Residuals:
##      Min       1Q   Median       3Q      Max 
## -1.71003 -0.11052  0.05804  0.14752  0.80139 
## 
## Coefficients:
##                     Estimate Std. Error t value Pr(>|t|)    
## (Intercept)           5.3071     0.2579  20.581 1.89e-14 ***
## treatmentdrought T1  -0.2569     0.3939  -0.652  0.52201    
## treatmentdrought T2  -0.5926     0.3647  -1.625  0.12066    
## treatmentdrought T3  -1.0874     0.3647  -2.982  0.00766 ** 
## treatmentdrought T4  -2.3133     0.3939  -5.873 1.18e-05 ***
## treatmentdrought T5  -4.4718     0.3647 -12.262 1.80e-10 ***
## treatmentrewetting   -0.8548     0.3647  -2.344  0.03009 *  
## ---
## Signif. codes:  0 '***' 0.001 '**' 0.01 '*' 0.05 '.' 0.1 ' ' 1
## 
## Residual standard error: 0.5157 on 19 degrees of freedom
## Multiple R-squared:  0.9175, Adjusted R-squared:  0.8914 
## F-statistic:  35.2 on 6 and 19 DF,  p-value: 2.639e-09
```

```
HSD.test(mod1,"treatment",group=T,unbalanced = T)$groups
```

```
mod1 <- lm(N2O ~ treatment , data = sample) 
any(sample$N2O==0)
```

```
## [1] FALSE
```

```
mod1l <- lm(log(N2O + abs(min(N2O))+psc) ~ treatment, data = sample) 
mod1sq <- lm(cbrt(N2O) ~ treatment, data = sample) 
plot(mod1)
plot(mod1l)
plot(mod1sq) # best
```

```
summary(mod1)
```

```
## 
## Call:
## lm(formula = N2O ~ treatment, data = sample)
## 
## Residuals:
##      Min       1Q   Median       3Q      Max 
## -1252.86  -114.33   -15.94    72.17  2946.11 
## 
## Coefficients:
##                     Estimate Std. Error t value Pr(>|t|)  
## (Intercept)           223.99     404.13   0.554   0.5859  
## treatmentdrought T1  -170.30     617.31  -0.276   0.7856  
## treatmentdrought T2   -50.06     571.52  -0.088   0.9311  
## treatmentdrought T3   -92.69     571.52  -0.162   0.8729  
## treatmentdrought T4  -176.80     617.31  -0.286   0.7777  
## treatmentdrought T5  -186.75     571.52  -0.327   0.7474  
## treatmentrewetting   1080.07     571.52   1.890   0.0741 .
## ---
## Signif. codes:  0 '***' 0.001 '**' 0.01 '*' 0.05 '.' 0.1 ' ' 1
## 
## Residual standard error: 808.3 on 19 degrees of freedom
## Multiple R-squared:  0.2823, Adjusted R-squared:  0.0557 
## F-statistic: 1.246 on 6 and 19 DF,  p-value: 0.3276
```

```
summary(mod1l)
```

```
## 
## Call:
## lm(formula = log(N2O + abs(min(N2O)) + psc) ~ treatment, data = sample)
## 
## Residuals:
##      Min       1Q   Median       3Q      Max 
## -23.4085  -0.3531   0.2028   0.5527   8.5464 
## 
## Coefficients:
##                     Estimate Std. Error t value Pr(>|t|)  
## (Intercept)           -1.920      3.132  -0.613   0.5471  
## treatmentdrought T1    7.054      4.784   1.475   0.1567  
## treatmentdrought T2    7.583      4.429   1.712   0.1032  
## treatmentdrought T3    7.220      4.429   1.630   0.1195  
## treatmentdrought T4    6.872      4.784   1.436   0.1671  
## treatmentdrought T5    6.631      4.429   1.497   0.1508  
## treatmentrewetting     8.392      4.429   1.895   0.0734 .
## ---
## Signif. codes:  0 '***' 0.001 '**' 0.01 '*' 0.05 '.' 0.1 ' ' 1
## 
## Residual standard error: 6.264 on 19 degrees of freedom
## Multiple R-squared:  0.2023, Adjusted R-squared:  -0.04957 
## F-statistic: 0.8032 on 6 and 19 DF,  p-value: 0.5796
```

```
summary(mod1sq)
```

```
## 
## Call:
## lm(formula = cbrt(N2O) ~ treatment, data = sample)
## 
## Residuals:
##     Min      1Q  Median      3Q     Max 
## -8.0071 -2.9376  0.5233  2.3465  7.6219 
## 
## Coefficients:
##                     Estimate Std. Error t value Pr(>|t|)
## (Intercept)           3.0740     2.3280   1.320    0.202
## treatmentdrought T1   0.4289     3.5560   0.121    0.905
## treatmentdrought T2   2.4331     3.2922   0.739    0.469
## treatmentdrought T3   0.2468     3.2922   0.075    0.941
## treatmentdrought T4  -1.3238     3.5560  -0.372    0.714
## treatmentdrought T5  -2.9107     3.2922  -0.884    0.388
## treatmentrewetting    5.5024     3.2922   1.671    0.111
## 
## Residual standard error: 4.656 on 19 degrees of freedom
## Multiple R-squared:  0.2942, Adjusted R-squared:  0.07134 
## F-statistic:  1.32 on 6 and 19 DF,  p-value: 0.2962
```

```
HSD.test(mod1sq,"treatment",group=T,unbalanced = T)$groups
```

```
mod1 <- lm(resp_RNA ~ treatment , data = sample) 
any(sample$resp_RNA==0)
```

```
## [1] FALSE
```

```
mod1l <- lm(log(resp_RNA) ~ treatment, data = sample) 
mod1sq <- lm(sqrt(resp_RNA) ~ treatment, data = sample) 
plot(mod1)
plot(mod1l)# best
plot(mod1sq)
```

```
summary(mod1)
```

```
## 
## Call:
## lm(formula = resp_RNA ~ treatment, data = sample)
## 
## Residuals:
##        Min         1Q     Median         3Q        Max 
## -0.0059745 -0.0008795 -0.0000569  0.0009021  0.0117371 
## 
## Coefficients:
##                      Estimate Std. Error t value Pr(>|t|)   
## (Intercept)          0.001568   0.001874   0.837   0.4132   
## treatmentdrought T1  0.001920   0.002862   0.671   0.5105   
## treatmentdrought T2  0.005994   0.002650   2.262   0.0356 * 
## treatmentdrought T3  0.008733   0.002650   3.296   0.0038 **
## treatmentdrought T4  0.003943   0.002862   1.378   0.1844   
## treatmentdrought T5 -0.001359   0.002650  -0.513   0.6140   
## treatmentrewetting   0.002687   0.002650   1.014   0.3232   
## ---
## Signif. codes:  0 '***' 0.001 '**' 0.01 '*' 0.05 '.' 0.1 ' ' 1
## 
## Residual standard error: 0.003747 on 19 degrees of freedom
## Multiple R-squared:  0.5168, Adjusted R-squared:  0.3642 
## F-statistic: 3.387 on 6 and 19 DF,  p-value: 0.0192
```

```
summary(mod1l)
```

```
## 
## Call:
## lm(formula = log(resp_RNA) ~ treatment, data = sample)
## 
## Residuals:
##      Min       1Q   Median       3Q      Max 
## -1.18094 -0.25592  0.06218  0.42335  0.95135 
## 
## Coefficients:
##                     Estimate Std. Error t value Pr(>|t|)    
## (Intercept)          -6.5080     0.3195 -20.370 2.28e-14 ***
## treatmentdrought T1   0.8163     0.4880   1.673 0.110792    
## treatmentdrought T2   1.5518     0.4518   3.434 0.002779 ** 
## treatmentdrought T3   1.7417     0.4518   3.855 0.001067 ** 
## treatmentdrought T4   1.0732     0.4880   2.199 0.040462 *  
## treatmentdrought T5  -2.1358     0.4518  -4.727 0.000147 ***
## treatmentrewetting    0.8688     0.4518   1.923 0.069603 .  
## ---
## Signif. codes:  0 '***' 0.001 '**' 0.01 '*' 0.05 '.' 0.1 ' ' 1
## 
## Residual standard error: 0.639 on 19 degrees of freedom
## Multiple R-squared:  0.8415, Adjusted R-squared:  0.7915 
## F-statistic: 16.81 on 6 and 19 DF,  p-value: 1.113e-06
```

```
summary(mod1sq)
```

```
## 
## Call:
## lm(formula = sqrt(resp_RNA) ~ treatment, data = sample)
## 
## Residuals:
##       Min        1Q    Median        3Q       Max 
## -0.030850 -0.008481  0.000362  0.007870  0.051827 
## 
## Coefficients:
##                     Estimate Std. Error t value Pr(>|t|)    
## (Intercept)          0.03911    0.01036   3.776 0.001277 ** 
## treatmentdrought T1  0.01945    0.01582   1.229 0.233985    
## treatmentdrought T2  0.04638    0.01465   3.166 0.005087 ** 
## treatmentdrought T3  0.05751    0.01465   3.926 0.000907 ***
## treatmentdrought T4  0.03130    0.01582   1.978 0.062555 .  
## treatmentdrought T5 -0.02522    0.01465  -1.721 0.101410    
## treatmentrewetting   0.02370    0.01465   1.618 0.122194    
## ---
## Signif. codes:  0 '***' 0.001 '**' 0.01 '*' 0.05 '.' 0.1 ' ' 1
## 
## Residual standard error: 0.02072 on 19 degrees of freedom
## Multiple R-squared:  0.6948, Adjusted R-squared:  0.5984 
## F-statistic: 7.209 on 6 and 19 DF,  p-value: 0.0004022
```

```
HSD.test(mod1l,"treatment",group=T,unbalanced = T)$groups
```

```
mod1 <- lm(DOC_DON_rat ~ treatment , data = sample) 
any(sample$DOC_DON_rat==0)
```

```
## [1] FALSE
```

```
mod1l <- lm(log(DOC_DON_rat) ~ treatment, data = sample) 
mod1sq <- lm(sqrt(DOC_DON_rat) ~ treatment, data = sample) 
plot(mod1)# best
plot(mod1l)
plot(mod1sq)
```

```
summary(mod1)
```

```
## 
## Call:
## lm(formula = DOC_DON_rat ~ treatment, data = sample)
## 
## Residuals:
##      Min       1Q   Median       3Q      Max 
## -0.81440 -0.13816  0.02504  0.16046  0.52329 
## 
## Coefficients:
##                     Estimate Std. Error t value Pr(>|t|)    
## (Intercept)           3.4022     0.1733  19.629 4.48e-14 ***
## treatmentdrought T1   0.4238     0.2648   1.601   0.1260    
## treatmentdrought T2   0.3823     0.2451   1.560   0.1354    
## treatmentdrought T3   0.1881     0.2451   0.767   0.4523    
## treatmentdrought T4   0.1343     0.2648   0.507   0.6179    
## treatmentdrought T5   0.1746     0.2451   0.712   0.4849    
## treatmentrewetting   -0.5252     0.2451  -2.143   0.0453 *  
## ---
## Signif. codes:  0 '***' 0.001 '**' 0.01 '*' 0.05 '.' 0.1 ' ' 1
## 
## Residual standard error: 0.3467 on 19 degrees of freedom
## Multiple R-squared:  0.5011, Adjusted R-squared:  0.3436 
## F-statistic: 3.181 on 6 and 19 DF,  p-value: 0.02472
```

```
summary(mod1l)
```

```
## 
## Call:
## lm(formula = log(DOC_DON_rat) ~ treatment, data = sample)
## 
## Residuals:
##       Min        1Q    Median        3Q       Max 
## -0.316117 -0.038561  0.006564  0.045288  0.171484 
## 
## Coefficients:
##                     Estimate Std. Error t value Pr(>|t|)    
## (Intercept)          1.21920    0.05691  21.423 9.09e-15 ***
## treatmentdrought T1  0.12258    0.08693   1.410   0.1747    
## treatmentdrought T2  0.10762    0.08048   1.337   0.1969    
## treatmentdrought T3  0.05833    0.08048   0.725   0.4775    
## treatmentdrought T4  0.04123    0.08693   0.474   0.6407    
## treatmentdrought T5  0.05441    0.08048   0.676   0.5072    
## treatmentrewetting  -0.17912    0.08048  -2.226   0.0384 *  
## ---
## Signif. codes:  0 '***' 0.001 '**' 0.01 '*' 0.05 '.' 0.1 ' ' 1
## 
## Residual standard error: 0.1138 on 19 degrees of freedom
## Multiple R-squared:  0.487,  Adjusted R-squared:  0.3251 
## F-statistic: 3.007 on 6 and 19 DF,  p-value: 0.03074
```

```
summary(mod1sq)
```

```
## 
## Call:
## lm(formula = sqrt(DOC_DON_rat) ~ treatment, data = sample)
## 
## Residuals:
##      Min       1Q   Median       3Q      Max 
## -0.25318 -0.03649  0.00641  0.04262  0.14334 
## 
## Coefficients:
##                     Estimate Std. Error t value Pr(>|t|)    
## (Intercept)          1.84209    0.04929  37.372   <2e-16 ***
## treatmentdrought T1  0.11391    0.07529   1.513   0.1468    
## treatmentdrought T2  0.10133    0.06971   1.454   0.1624    
## treatmentdrought T3  0.05239    0.06971   0.752   0.4615    
## treatmentdrought T4  0.03719    0.07529   0.494   0.6270    
## treatmentdrought T5  0.04875    0.06971   0.699   0.4928    
## treatmentrewetting  -0.15274    0.06971  -2.191   0.0411 *  
## ---
## Signif. codes:  0 '***' 0.001 '**' 0.01 '*' 0.05 '.' 0.1 ' ' 1
## 
## Residual standard error: 0.09858 on 19 degrees of freedom
## Multiple R-squared:  0.4958, Adjusted R-squared:  0.3366 
## F-statistic: 3.114 on 6 and 19 DF,  p-value: 0.02686
```

```
HSD.test(mod1,"treatment",group=T,unbalanced = T)$groups
```

```
mod1 <- lm(DOC_DON_DIN_rat ~ treatment , data = sample) 
any(sample$DOC_DON_DIN_rat==0)
```

```
## [1] FALSE
```

```
mod1l <- lm(log(DOC_DON_DIN_rat) ~ treatment, data = sample) 
mod1sq <- lm(sqrt(DOC_DON_DIN_rat) ~ treatment, data = sample) 
plot(mod1)# best
plot(mod1l)
plot(mod1sq)
```

```
summary(mod1)
```

```
## 
## Call:
## lm(formula = DOC_DON_DIN_rat ~ treatment, data = sample)
## 
## Residuals:
##      Min       1Q   Median       3Q      Max 
## -0.22992 -0.09647 -0.01733  0.08762  0.29652 
## 
## Coefficients:
##                     Estimate Std. Error t value Pr(>|t|)    
## (Intercept)          0.71263    0.07767   9.175 2.07e-08 ***
## treatmentdrought T1  0.14711    0.11864   1.240   0.2301    
## treatmentdrought T2  0.14599    0.10984   1.329   0.1995    
## treatmentdrought T3  0.15304    0.10984   1.393   0.1796    
## treatmentdrought T4 -0.24233    0.11864  -2.043   0.0552 .  
## treatmentdrought T5 -0.09646    0.10984  -0.878   0.3908    
## treatmentrewetting  -0.25529    0.10984  -2.324   0.0314 *  
## ---
## Signif. codes:  0 '***' 0.001 '**' 0.01 '*' 0.05 '.' 0.1 ' ' 1
## 
## Residual standard error: 0.1553 on 19 degrees of freedom
## Multiple R-squared:  0.6071, Adjusted R-squared:  0.4831 
## F-statistic: 4.894 on 6 and 19 DF,  p-value: 0.003515
```

```
summary(mod1l)
```

```
## 
## Call:
## lm(formula = log(DOC_DON_DIN_rat) ~ treatment, data = sample)
## 
## Residuals:
##      Min       1Q   Median       3Q      Max 
## -0.56863 -0.11607 -0.00098  0.17465  0.37507 
## 
## Coefficients:
##                     Estimate Std. Error t value Pr(>|t|)  
## (Intercept)          -0.3520     0.1239  -2.842   0.0104 *
## treatmentdrought T1   0.1943     0.1892   1.027   0.3173  
## treatmentdrought T2   0.1820     0.1752   1.039   0.3119  
## treatmentdrought T3   0.1867     0.1752   1.066   0.2998  
## treatmentdrought T4  -0.4175     0.1892  -2.207   0.0398 *
## treatmentdrought T5  -0.1465     0.1752  -0.836   0.4134  
## treatmentrewetting   -0.4877     0.1752  -2.784   0.0118 *
## ---
## Signif. codes:  0 '***' 0.001 '**' 0.01 '*' 0.05 '.' 0.1 ' ' 1
## 
## Residual standard error: 0.2477 on 19 degrees of freedom
## Multiple R-squared:  0.6108, Adjusted R-squared:  0.488 
## F-statistic: 4.971 on 6 and 19 DF,  p-value: 0.003245
```

```
summary(mod1sq)
```

```
## 
## Call:
## lm(formula = sqrt(DOC_DON_DIN_rat) ~ treatment, data = sample)
## 
## Residuals:
##       Min        1Q    Median        3Q       Max 
## -0.172602 -0.052801 -0.004901  0.061342  0.152588 
## 
## Coefficients:
##                     Estimate Std. Error t value Pr(>|t|)    
## (Intercept)          0.84140    0.04742  17.743 2.77e-13 ***
## treatmentdrought T1  0.08429    0.07244   1.164   0.2590    
## treatmentdrought T2  0.08121    0.06706   1.211   0.2408    
## treatmentdrought T3  0.08406    0.06706   1.253   0.2252    
## treatmentdrought T4 -0.15817    0.07244  -2.184   0.0417 *  
## treatmentdrought T5 -0.05924    0.06706  -0.883   0.3881    
## treatmentrewetting  -0.17429    0.06706  -2.599   0.0176 *  
## ---
## Signif. codes:  0 '***' 0.001 '**' 0.01 '*' 0.05 '.' 0.1 ' ' 1
## 
## Residual standard error: 0.09484 on 19 degrees of freedom
## Multiple R-squared:  0.6186, Adjusted R-squared:  0.4981 
## F-statistic: 5.135 on 6 and 19 DF,  p-value: 0.00274
```

```
HSD.test(mod1,"treatment",group=T,unbalanced = T)$groups
```
