# Supplementary material for: Arctic Soil C and N Cycling Are Linked With Microbial Adaptations During Drought
Source: Glob Chang Biol. 2025 Sep 18;31(9):e70502. doi: 10.1111/gcb.70502 (PMC12445406; doi:10.1111/gcb.70502)
Supplement: Supplementary file 4 — Data S2: gcb70502‐sup‐0004‐Supinfo2.html. [file GCB-31-e70502-s004.html]

Data analysis for the study Arctic soil C and N cycling are linked with microbial adaptations during drought


# Data analysis for the study Arctic soil C and N cycling are linked with microbial adaptations during drought

# Data analysis for the study Arctic soil C and N cycling are linked with microbial adaptations during drought - regression between gene expression and microbial activity /soil chemistry

Relationship between gene expression and activity/soil chemistry with
dream (can use random factor to take into account replicates) ##
Setup

```
library(variancePartition)
library(edgeR)
library(BiocParallel)
library(tidyverse)
```

```
## ── Attaching core tidyverse packages ──────────────────────── tidyverse 2.0.0 ──
## ✔ dplyr     1.1.4     ✔ readr     2.1.5
## ✔ forcats   1.0.0     ✔ stringr   1.5.1
## ✔ lubridate 1.9.3     ✔ tibble    3.2.1
## ✔ purrr     1.0.2     ✔ tidyr     1.3.1
## ── Conflicts ────────────────────────────────────────── tidyverse_conflicts() ──
## ✖ dplyr::filter() masks stats::filter()
## ✖ dplyr::lag()    masks stats::lag()
## ℹ Use the conflicted package (<http://conflicted.r-lib.org/>) to force all conflicts to become errors
```

```
sessionInfo()
```

```
## R version 4.3.3 (2024-02-29 ucrt)
## Platform: x86_64-w64-mingw32/x64 (64-bit)
## Running under: Windows 11 x64 (build 26100)
## 
## Matrix products: default
## 
## 
## locale:
## [1] LC_COLLATE=English_United States.utf8 
## [2] LC_CTYPE=English_United States.utf8   
## [3] LC_MONETARY=English_United States.utf8
## [4] LC_NUMERIC=C                          
## [5] LC_TIME=English_United States.utf8    
## 
## time zone: Europe/Copenhagen
## tzcode source: internal
## 
## attached base packages:
## [1] stats     graphics  grDevices utils     datasets  methods   base     
## 
## other attached packages:
##  [1] lubridate_1.9.3          forcats_1.0.0            stringr_1.5.1           
##  [4] dplyr_1.1.4              purrr_1.0.2              readr_2.1.5             
##  [7] tidyr_1.3.1              tibble_3.2.1             tidyverse_2.0.0         
## [10] edgeR_4.0.16             variancePartition_1.32.5 BiocParallel_1.36.0     
## [13] limma_3.58.1             ggplot2_3.5.0           
## 
## loaded via a namespace (and not attached):
##  [1] tidyselect_1.2.1    farver_2.1.1        bitops_1.0-7       
##  [4] fastmap_1.2.0       digest_0.6.35       timechange_0.3.0   
##  [7] lifecycle_1.0.4     statmod_1.5.0       magrittr_2.0.3     
## [10] compiler_4.3.3      rlang_1.1.3         sass_0.4.10        
## [13] tools_4.3.3         yaml_2.3.8          knitr_1.50         
## [16] plyr_1.8.9          RColorBrewer_1.1-3  KernSmooth_2.23-26 
## [19] withr_3.0.2         numDeriv_2016.8-1.1 BiocGenerics_0.48.1
## [22] grid_4.3.3          aod_1.3.3           caTools_1.18.3     
## [25] colorspace_2.1-0    scales_1.4.0        gtools_3.9.5       
## [28] iterators_1.0.14    MASS_7.3-60.0.1     cli_3.6.2          
## [31] mvtnorm_1.2-4       rmarkdown_2.29      reformulas_0.4.1   
## [34] generics_0.1.4      rstudioapi_0.17.1   tzdb_0.4.0         
## [37] reshape2_1.4.4      minqa_1.2.6         cachem_1.1.0       
## [40] splines_4.3.3       parallel_4.3.3      matrixStats_1.2.0  
## [43] vctrs_0.6.5         boot_1.3-31         Matrix_1.6-5       
## [46] jsonlite_1.8.8      hms_1.1.3           pbkrtest_0.5.4     
## [49] locfit_1.5-9.10     jquerylib_0.1.4     glue_1.7.0         
## [52] nloptr_2.0.3        codetools_0.2-20    stringi_1.8.3      
## [55] gtable_0.3.6        EnvStats_3.1.0      lme4_1.1-37        
## [58] lmerTest_3.1-3      remaCor_0.0.18      pillar_1.10.2      
## [61] htmltools_0.5.8.1   gplots_3.2.0        R6_2.6.1           
## [64] Rdpack_2.6.4        evaluate_1.0.3      lattice_0.22-5     
## [67] Biobase_2.62.0      rbibutils_2.3       backports_1.5.0    
## [70] RhpcBLASctl_0.23-42 broom_1.0.8         fANCOVA_0.6-1      
## [73] corpcor_1.6.10      bslib_0.9.0         Rcpp_1.0.12        
## [76] nlme_3.1-164        xfun_0.52           pkgconfig_2.0.3
```

## function for aggregating gene counts

```
#countab: counttable only numeric part,taxa are rows
#taxo=taxonomy table
#col2matchcount vector of rownames or column in counttab to which order of taxonomy table should be matched
#col2matchtax:vector of rownames or column in taxo to match
#Taxlevel: taxonomic level on which should be aggregated
#Samp: sample data
#fac: factor in Sample of which the mean should be made
#Summarize: should rare taxa be summarized and represented as others
#sumlevel: abundance threshold below which taxa are summarkued as others

AbuTableInteger=function(countab,taxo,col2matchcount,col2matchtax,Taxlevel,Samp,fac,Summarize=F,sumlevel=NULL){
  #replace NA with unclassified
  taxo=as.data.frame(apply(taxo,2,function(x){
    sapply(x,function(y){ifelse(is.na(y),"unclassified",y)})
  }))
  
  
  tax=taxo[match(col2matchcount,col2matchtax),]
  
  
  
  ## Abundance at chosen level
  taxabu=aggregate(countab,list(tax[,Taxlevel]),sum)
  rownames(taxabu)=taxabu[,1]
  taxabu=taxabu[,-1]
  
  taxabu <- as.data.frame(t(taxabu))
  
  for(i in 1:ncol(Samp)){
    Samp[,i]=as.character(Samp[,i])
  }
  
  # reorder rows of aggregated count table according to sample if not in same order
  if (!(all(rownames(taxabu)==rownames(Samp)))){
    taxabu <- taxab[rownames(Samp),]
  }
  
  taxabu.mean=aggregate(taxabu,list(Samp[,fac]),mean)
  colnames(taxabu.mean)[1]=fac
  rownames(taxabu.mean)=taxabu.mean[,1]
  
  a=c()
  for (i in colnames(taxabu.mean)){
    a[i]=is.numeric(taxabu.mean[,i])
  }
  
  taxabu.mean=taxabu.mean[,a]
  
  
  if(Summarize==T){
    num=taxabu.mean
    num.l=as.list(as.data.frame(t(num)))
    
    num.l=lapply(num.l,function(x){
      names(x)=colnames(num)
      Others=sum(x[which(x<sumlevel)])
      x=x[-which(x<sumlevel)]
      names(Others)="Others"
      x=c(x,Others)
    })
    taxabu.mean <-as.data.frame(do.call(rbind, lapply(num.l, "[", unique(unlist(sapply(num.l,names))))))
    
    colnames(taxabu.mean)=unique(unlist(sapply(num.l,names)))
    
    taxabu.mean=apply(taxabu.rel.mean,2,function(x){
      sapply(x,function(y){ifelse(is.na(y),0,y)})
    })
    
  }
  
  
  a=as.data.frame(Samp[!duplicated(Samp[,fac]),])
  
  if(all(rownames(taxabu.mean)==a[,fac])){
    taxabu.mean=cbind(taxabu.mean,a)
  }else{
    taxabu.mean=taxabu.mean[match(a[,fac],rownames(taxabu.mean)),]
    taxabu.mean=cbind(taxabu.mean,a)
  }
  
  
  return(taxabu.mean)
}
```

## Import sample file

```
# Samples sheet with design and metadata (microbial activity + soil chemistry)
sample <- read.csv("../input/sample.csv", sep = ",") 
rownames(sample) <- sample[,1]
# remove samples with too few reads (sample 6: no reads mapped; sample 19: about 300 reads mapped)
sample <- sample[-c(6,19),]

# RNA contents per g dry weight
```

## SEED

### Import count table

```
#### Import count table
count <- read.csv("../input/contigAbundanceAll.tsv", sep = "\t",row.names = 1) 
colnames(count) <- gsub("^X","",colnames(count))
# remove samples with too few reads (sample 6: no reads mapped; sample 19: about 300 reads mapped) and reorder count data according to sample sheet
count <- count[,rownames(sample)]

#### Import annotation tables
# seed
seed <- read.csv("../input/SEED.csv", sep = ",",row.names = 1)
#replace NA with "unclassified"
for (i in 2:6){
  seed[,i][is.na(seed[,i])] <- "unclassified"
}


# duplicate contigs with different level 4 annotations in count table 
seedLvl4Unique <- seed
# get combinations of contig and level 4
seedLvl4Unique[,"contigLvl4"] <- paste(seedLvl4Unique$contig,seedLvl4Unique$lvl4,sep = ";")
seedLvl4Unique <- seedLvl4Unique[!duplicated(seedLvl4Unique$contigLvl4),]

# duplicate contigs in count table if there are multiple annotations

#remove suffix .1 for multiple annotations to make name rownames equal to those in count table for first occurrence
rownames(seedLvl4Unique) <- gsub("\\.1$","",rownames(seedLvl4Unique))


for (i in rownames(seedLvl4Unique)[!(rownames(seedLvl4Unique)%in%rownames(count))]){
  count[i,1:26] <- count[rownames(count)==seedLvl4Unique[i,"contig"],1:26]
}

# add column with combinations of all levels to (full) annotation table => to add all combinations of possible annotations after running DESEq
seed$lvl1.lvl2.lvl3 <- paste(seed$lvl1, seed$lvl2, seed$lvl3, sep = ";;")
# aggregate at lvl4 (gene)
countLvl4 <- AbuTableInteger(countab = count, taxo = seedLvl4Unique, col2matchcount = rownames(count),
                             col2matchtax = rownames(seedLvl4Unique), Taxlevel = "lvl4", Samp = sample, fac = "sampleID")

#remove sample data
countLvl4 <- countLvl4[,-(1359:1372)]

#transpose
countLvl4 <- as.data.frame(t(countLvl4))

## use only a subset of genes in level 1 categories of interest to reduce multiple testing burden 
# categories of interest
cat <- c("Respiration", "Protein Metabolism", "RNA Metabolism", "DNA Metabolism", "Stress Response", "Membrane Transport", "Amino Acids and Derivatives", "Motility and Chemotaxis",                   
         "Regulation and Cell signaling","Nucleosides and Nucleotides", "Cell Wall and Capsule", "Phosphorus Metabolism", "Cell Division and Cell Cycle", "Dormancy and Sporulation")                          

#subset aggregated count table
countLvl4 <- countLvl4[rownames(countLvl4)%in%seedLvl4Unique$lvl4[seedLvl4Unique$lvl1%in%cat],]
```

### Dream analysis

```
# filter genes by number of counts
isexpr = rowSums(cpm(countLvl4)>0.1) >= 5 # at least 5 samples where cpm > 0.1

# create DGEList 
geneExpr = DGEList( countLvl4[isexpr,] )

# calcNormFactors (edgeR): Calculate scaling factors to convert raw library sizes into effective library sizes
# normalization done to account for differences in library sizes, cpm values based on normalized count data
geneExpr = calcNormFactors( geneExpr )

# scale variables where model cannot be fit otherwise (CH4)
sample_sc <- sample
sample_sc$CH4 <- scale(sample_sc$CH4, center = F)

res <- list()
for (i in colnames(sample_sc)[3:14]){
  form <- as.formula(paste("~",i, "+ (1|treatment)")) 
  vobjDream = voomWithDreamWeights( geneExpr, form, sample_sc )
  fitmm = dream( vobjDream, form, sample_sc )
  res[[i]] <- topTable( fitmm, coef=i, number=nrow(fitmm$coefficients) )
  res[[i]][,"variable"] <- rep(i, nrow( res[[i]]))
}


# subset to significant padj.
res0.05 <- list()
for (i in names(res)){
  res0.05[[i]] <- res[[i]][res[[i]][,'adj.P.Val']<0.05,]
}

# create table only with lvl4 as name
# vector with all genes that have a sign. association with any of the variables
genes <- unique(unlist(lapply(res0.05, function(x)rownames(x))))
resAll_0.05 <- data.frame()
for (i in genes){
  for (j in names(res0.05)){
    resAll_0.05[i,j] <- ifelse(i%in%rownames(res0.05[[j]]),res0.05[[j]][i,"adj.P.Val"],NA)
  }
}


# reorder according to annotation hierarchy
#vector with annotations in output table
ord <- seed[seed$lvl4%in%rownames(resAll_0.05),]
ord <- ord[order(ord$lvl1,ord$lvl2,ord$lvl3,ord$lvl4),] #reorder according to all categories
ord <- ord[!duplicated(paste(ord$lvl4)),] # keep only one entry for each level 4 category
a <- resAll_0.05[match(ord$lvl4,rownames(resAll_0.05)),]
rm(ord)

#add annotation
a <- list()
for(i in rownames(resAll_0.05)){
  a[[i]] <- data.frame()
  for (j in 1:length(unique(paste(seed[seed$lvl4==i,"lvl1.lvl2.lvl3"],i)))){
    a[[i]][j,colnames(resAll_0.05)] <- resAll_0.05[i,colnames(resAll_0.05)]
    a[[i]][j,"annotation"] <- unique(paste(seed[seed$lvl4==i,"lvl1.lvl2.lvl3"],i,sep=";;"))[j]
  }
}
#combine
resAll_0.05 <- do.call(rbind,a)
# separate annotation
resAll_0.05 <- separate(data=resAll_0.05,col=annotation, into = c("lvl1","lvl2","lvl3","lvl4"),sep = ";;",remove = F)
rownames(resAll_0.05) <- make.unique(resAll_0.05[,"lvl4"], sep=".")

resAll_0.05
```

## Cazy

### Import count table

```
#### Import count table
count <- read.csv("../input/contigAbundanceAll.tsv", sep = "\t",row.names = 1) 
colnames(count) <- gsub("^X","",colnames(count))
# remove samples with too few reads (sample 6: no reads mapped; sample 19: about 300 reads mapped) and reorder count data according to sample sheet
count <- count[,rownames(sample)]

#### Import annotation tables
# Cazy
Cazy <- read.csv("../input/CAZY.csv", sep = ",",row.names = 1)
#replace NA with "unclassified"
for (i in 2:6){
  Cazy[,i][is.na(Cazy[,i])] <- "unclassified"
}

# aggreagate at family level
countFam <- AbuTableInteger(countab = count, taxo = Cazy, col2matchcount = rownames(count),
                             col2matchtax = rownames(Cazy), Taxlevel = "Family", Samp = sample, fac = "sampleID")

#remove sample data
countFam <- countFam[,-(315:328)]

#transpose
countFam <- as.data.frame(t(countFam))
```

### Dream analysis

```
isexpr = rowSums(cpm(countFam)>0.1) >= 5 # at least 5 samples where cpm > 0.1

# create DGEList 
geneExpr = DGEList( countFam[isexpr,] )

# calcNormFactors (edgeR): Calculate scaling factors to convert raw library sizes into effective library sizes
# normalization done to account for differences in library sizes, cpm values based on normalized count data
geneExpr = calcNormFactors( geneExpr )

res <- list()
for (i in colnames(sample_sc)[3:14]){
  form <- as.formula(paste("~",i, "+ (1|treatment)")) 
  vobjDream = voomWithDreamWeights( geneExpr, form, sample_sc )
  fitmm = dream( vobjDream, form, sample_sc )
  res[[i]] <- topTable( fitmm, coef=i, number=nrow(fitmm$coefficients) )
  res[[i]][,"variable"] <- rep(i, nrow( res[[i]]))
}


# subset to significant padj.
res0.05 <- list()
for (i in names(res)){
  res0.05[[i]] <- res[[i]][res[[i]][,'adj.P.Val']<0.05,]
}

# vector with all genes that have a sign. association with any of the variables
genes <- unique(unlist(lapply(res0.05, function(x)rownames(x))))

resAll_0.05 <- data.frame()
for (i in genes){
  for (j in names(res0.05)){
    resAll_0.05[i,j] <- ifelse(i%in%rownames(res0.05[[j]]),res0.05[[j]][i,"adj.P.Val"],NA)
  }
}

resAll_0.05
```

## Ncyc

### Import count table

```
count <- read.csv("../input/contigAbundanceAll.tsv", sep = "\t",row.names = 1) 
colnames(count) <- gsub("^X","",colnames(count))
# remove samples with too few reads (sample 6: no reads mapped; sample 19: about 300 reads mapped) and reorder count data according to sample sheet
count <- count[,rownames(sample)]

#### Import annotation tables
# Ncyc
Ncyc <- read.csv("../input/NcycFinal.csv", sep = ",",row.names = 1)
#replace NA with "unclassified"
for (i in 2:7){
  Ncyc[,i][is.na(Ncyc[,i])] <- "unclassified"
}
# remove spaces in beginning of lvl1and lvl2
Ncyc$lvl1 <- gsub("^ ","",Ncyc$lvl1)
Ncyc$lvl2 <- gsub("^ ","",Ncyc$lvl2)

# duplicate contigs in count table if there are multiple annotations such that rownames of count table match annotation table
for (i in rownames(Ncyc)[!(rownames(Ncyc)%in%rownames(count))]){
  count[i,1:26] <- count[rownames(count)==Ncyc[i,"contig"],1:26]
}

countLvl2 <- AbuTableInteger(countab = count, taxo = Ncyc, col2matchcount = rownames(count),
                             col2matchtax = rownames(Ncyc), Taxlevel = "lvl2", Samp = sample, fac = "sampleID")

#remove sample data
countLvl2 <- countLvl2[,-(49:62)]

#transpose
countLvl2 <- as.data.frame(t(countLvl2))
```

### Dream analysis

```
# filter genes by number of counts
isexpr = rowSums(cpm(countLvl2)>0.1) >= 5 # at least 5 samples where cpm > 0.1

# create DGEList 
geneExpr = DGEList( countLvl2[isexpr,] )

# calcNormFactors (edgeR): Calculate scaling factors to convert raw library sizes into effective library sizes
# normalization done to account for differences in library sizes, cpm values based on normalized count data
geneExpr = calcNormFactors( geneExpr )

res <- list()
for (i in colnames(sample_sc)[3:14]){
  form <- as.formula(paste("~",i, "+ (1|treatment)")) 
  vobjDream = voomWithDreamWeights( geneExpr, form, sample_sc )
  fitmm = dream( vobjDream, form, sample_sc )
  res[[i]] <- topTable( fitmm, coef=i, number=nrow(fitmm$coefficients) )
  res[[i]][,"variable"] <- rep(i, nrow( res[[i]]))
}


# subset to significant padj.
res0.05 <- list()
for (i in names(res)){
  res0.05[[i]] <- res[[i]][res[[i]][,'adj.P.Val']<0.05,]
}

# create table only with lvl2 as name
# vector with all genes that have a sign. association with any of the variables
genes <- unique(unlist(lapply(res0.05, function(x)rownames(x))))
resAll_0.05 <- data.frame()
for (i in genes){
  for (j in names(res0.05)){
    resAll_0.05[i,j] <- ifelse(i%in%rownames(res0.05[[j]]),res0.05[[j]][i,"adj.P.Val"],NA)
  }
}

# reorder according to annotation hierarchy
#vector with annotations in output table
ord <- Ncyc[Ncyc$lvl2%in%rownames(resAll_0.05),]
ord <- ord[order(ord$lvl1,ord$lvl2),]
ord <- ord[!duplicated(paste(ord$lvl1,ord$lvl2)),]
a <- resAll_0.05[match(ord$lvl2,rownames(resAll_0.05)),]
rm(ord)

#add annotation
a <- list()
for(i in rownames(resAll_0.05)){
  a[[i]] <- data.frame()
  for (j in 1:length(unique(paste(Ncyc[Ncyc$lvl2==i,"lvl1"],i)))){
    a[[i]][j,colnames(resAll_0.05)] <- resAll_0.05[i,colnames(resAll_0.05)]
    a[[i]][j,"annotation"] <- unique(paste(Ncyc[Ncyc$lvl2==i,"lvl1"],i,sep=";"))[j]
  }
}
#combine
resAll_0.05 <- do.call(rbind,a)
# separate annotation
library(tidyr)
resAll_0.05 <- separate(data=resAll_0.05,col=annotation, into = c("lvl1","lvl2"),sep = ";",remove = F)
rownames(resAll_0.05) <- make.unique(resAll_0.05[,"lvl2"], sep=".")
resAll_0.05
```
